# A multi-faceted hospital-based intervention for intrapartum care in sub-Saharan Africa: a stepped-wedge cluster-randomized trial

**DOI:** 10.1038/s41591-026-04599-w

**Published:** 2026-08-26

**Authors:** Claudia Hanson, Joseph Akuze, Kristi Sidney Annerstedt, Helle Mölsted Alvesson, Sunjuri Sun, Manuela Straneo, Nicola Orsini, Lenka Benova, Mechthild M. Gross, Bruno Marchal, Rian Snijders, Effie Chipeta, Peter Waiswa, Hussein Kidanto, Jean-Paul Dossou, Andrea Barnabas Pembe, the ALERT Consortium, Kristi Sidney Annerstedt, Helle Mölsted Alvesson, Sunjuri Sun, Manuela Straneo, Nicola Orsini, Lenka Benova, Mechthild M. Gross, Bruno Marchal, Rian Snijders, Effie Chipeta, Jean-Paul Dossou, Claudia Hanson, Regine Unkels, Soha El Halabi, Ann-Beth Nygaard Moller, Veronika Tirado, Anastasia Månsson, Maria del Rosario Alsina, Sibylle Herzig van Wees, Joseph Akuze, Peter Waiswa, Elizabeth O. Ayebare, Gorrette Nalwadda, Josephine Babirye, Philip Wanduru, Gertrude Namazzi, Virginia Castellano Pleguezuelo, Therese Delvaux, Jil Molenaar, Joanne Welsh, Bianca Kandeya, Samuel Meja, William Stones, Yesaya Z. Nyirenda, Hussein L. Kidanto, Muzdalifat Abeid, Tumbwene Mwansisya, Zamoyoni Julius, Antoinette Sognonvi, Armelle Vigan, Christelle Boyi Metogni, Gisele Houngbo, Gottfried Agballa, Christian Agossou, Andrea B. Pembe, Fadhlun M. Alwy Al-Beity, Dickson Mkoka, Lilian T. Mselle, Beatrice Mwilike

**Affiliations:** 1 Department of Global Public Health, Karolinska Institutet, Stockholm, Sweden; 2 Department of Disease Control, London School of Hygiene and Tropical Medicine, London, UK; 3 Department of Infectious Disease Epidemiology and International Health, London School of Hygiene and Tropical Medicine, London, UK; 4 Centre of Excellence for Maternal, Newborn and Child Health, Makerere University School of Public Health, Kampala, Uganda; 5 Department of Public Health, Institute of Tropical Medicine, Antwerp, Belgium; 6 Midwifery Research and Education Unit, Hannover Medical School, Hannover, Germany; 7 Centre for Reproductive Health, Kamuzu University of Health Science, Blantyre, Malawi; 8 Aga Khan University, Dar es Salaam, Tanzania; 9 Centre de Recherche en Reproduction Humaine et en Démographie (CERRHUD), Cotonou, Benin; 10 Department of Obstetrics and Gynaecology, Muhimbili University of Health and Allied Sciences, Dar es Salaam, Tanzania

**Keywords:** Health services, Medical research

## Abstract

Around three million babies are stillborn or die shortly after birth each year worldwide, underscoring a persistent and unacceptable burden on families and health systems. To help address this challenge, we developed the facility-based, multi-faceted Action Leveraging Evidence to reduce perinatal Mortality and Morbidity (ALERT) intervention. ALERT promoted essential practices of intrapartum childbirth using (i) co-design, (ii) training, (iii) quality improvement and (iv) leadership mentoring. We evaluated ALERT using a stepped-wedge cluster-randomized trial with five 6-month periods across 16 hospitals in Benin, Malawi, Tanzania and Uganda. Among 134,630 women (including 139,300 neonates), the intervention was associated with 22% lower odds of the composite co-primary outcome, early perinatal mortality, defined as fresh stillbirth and 24 h neonatal mortality (adjusted odds ratio 0.78, 95% confidence interval 0.65–0.94), but not with the co-primary outcome, fresh stillbirth (adjusted odds ratio 0.88, 95% confidence interval 0.68–1.13). Despite the limitation of only including 16 hospitals, the diversity of settings across four sub-Saharan African countries supports the finding that early perinatal mortality and morbidity could be reduced through implementation of multi-faceted, context-specific strategies to build maternity provider capacities. Pan African Clinical Trial Registry: 202006793783148.

## Abstract (web summary)

A stepped-wedge cluster-randomized trial showed that implementation of a multi-faceted ALERT intervention that promoted essential practices of intrapartum childbirth reduced the odds of early perinatal mortality but not stillbirth across 16 hospitals in Benin, Malawi, Tanzania and Uganda.

## Main

High maternal and perinatal mortality and morbidity rates prevail in sub-Saharan African countries (SSA)^1–3^. Nearly half of the global 1.9 million stillbirths and 2.3 million neonatal deaths annually occur in SSA, where both stillbirth and neonatal mortality rates remain above 20 per 1,000 births^2^. In addition, poorly managed labor and childbirth care leads to a large burden of morbidity, including cerebral palsy and neurological disorders caused by oxygen deprivation^4–6^.

Following the substantial increases in facility-based birth since the early 2000s, now exceeding 80% globally and over 60% in SSA today^7,8^, attention has shifted in recent years to the quality of pregnancy and childbirth care^9,10^. It is within this context that the World Health Organization (WHO) published a framework defining the quality of pregnancy and childbirth care in 2015^11^ and recommendations for routine intrapartum care in 2018^12^. This document complemented earlier guidance that focused on the management of the main maternal complications such as postpartum hemorrhage^13^, pre-eclampsia/eclampsia^14^, infection^15^ and preterm birth^16^. The later focus on the management of complications stimulated the development and evaluation of a large number of training programs^17–19^. For example, the Preterm Birth Initiative focused on an intervention package addressing specifically prematurity-related intra- and immediate postnatal care, including administration of corticosteroids, resuscitation and kangaroo care^20^. The Nepal quality improvement package equally focused on resuscitation as an important component of immediate postpartum care^21^. In contrast, no training program operationalizing the WHO 2018 intrapartum care guideline has been developed, beyond trials assessing distinct tools, such as the Safe Childbirth Checklist^22^ or the new WHO partograph^23^. Intrapartum care, however, is complex and demands a multitude of skills and decisions within multi-professional maternity care teams including readiness to apply the packages to address complications. The lack of attention to routine care, however, hinders programming for the Every Woman Every Newborn Everywhere (EWENE) global initiative, midwifery care models as well as more efficient management when complications occur^24^.

Guidelines and trainings are important steps towards improved care; however, achieving high-quality care requires a wider perspective. A variety of strategies have been proposed, spanning from certification and accreditation to quality assurance, collaborative quality improvement, and audit and feedback^25–27^. Reviews on what works to improve healthcare providers’ adherence to evidence-based guidelines have indicated that combinations of strategies including quality improvement strategies to build problem-solving competences show the greatest effects^28–30^. However, strategies with too many components are challenging to implement. The optimal combinations of implementation strategies addressing the complexity of intrapartum care considering implementability and scalability are yet to be defined. Furthermore, while co-design has been identified as an important strategy to shape contextually relevant solutions, the operationalization and value within quality improvement programs are unknown^31^.

**Fig. 1:**
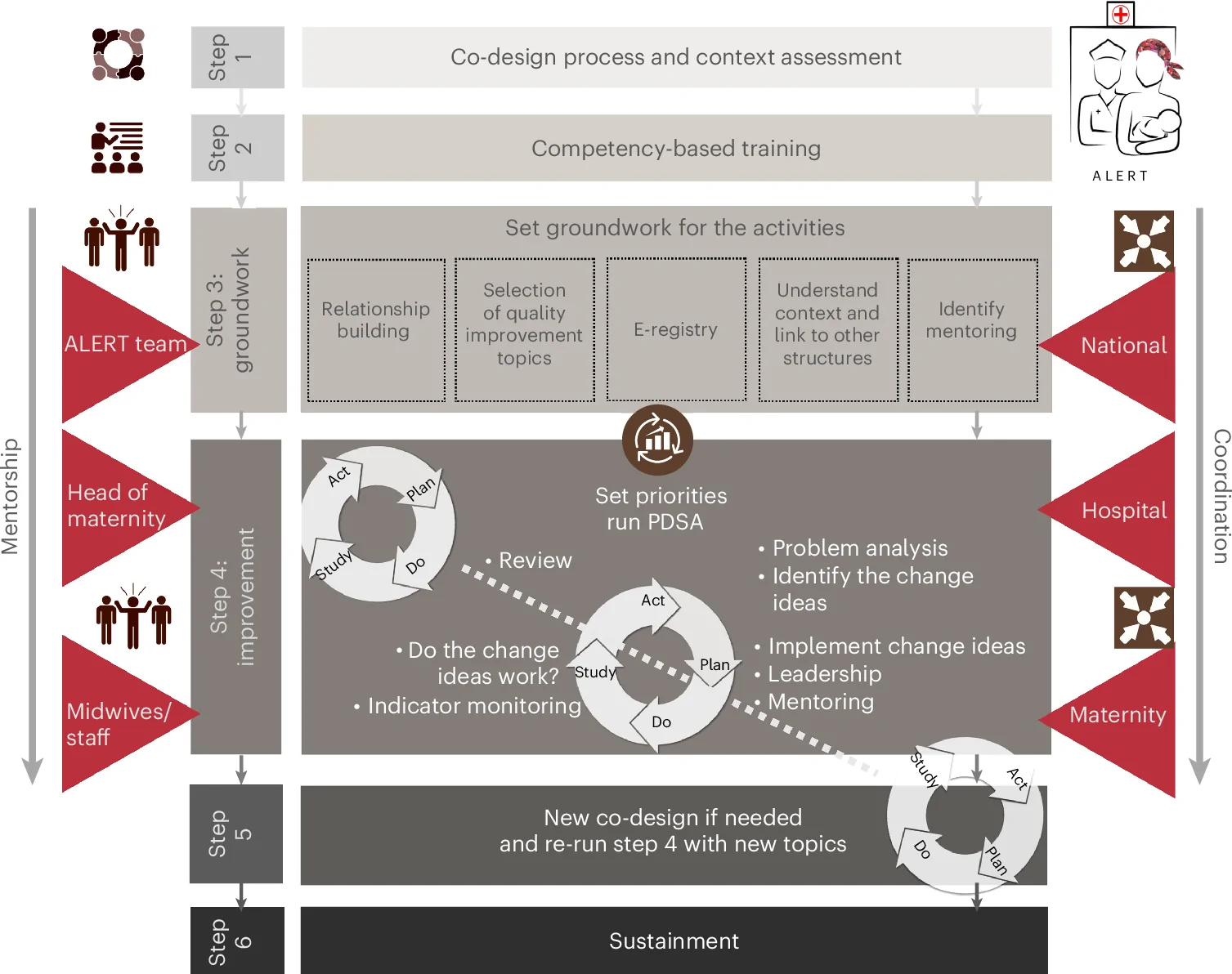
The ALERT intervention and conceptual framework. The intervention started with a co-design process (January and September 2021) (Step 1). Subsequently, training materials were developed addressing the knowledge deficits and implementation challenges. The final training modules covered: (a) admission standards, (b) intrapartum monitoring, (c) emergency preparedness, (d) respectful care, (e) labor and birth positioning and (f) communication and teamwork. Three-day competency-based in-facility trainings were carried out supported by Laerdal Global Health MamaBirthie low-cost models (Step 2). In parallel to the training, the ALERT in-country implementation team was to understand the specific hospital setting, build relationships and prepare for the quality improvement (Step 3). Quality improvement used the Plan-Do-Study-Act (PDSA) cycles and promoted the documentation using quality improvement journals. Quarterly on-site mentoring and frequent phone calls supported the quality improvement. Change ideas were developed around the six training modules. The quality improvement was supported by continuous data analysis from the ALERT perinatal e-registry (Step 4). New co-design if needed (Step 5) and sustained improvement work (Step 6). Credit: Figure created by Noon Pakavaleetorn, noon.pakavaleetorn@gmail.com, using Canva Pro.

The Action Leveraging Evidence to reduce perinatal Mortality and Morbidity in sub-Saharan Africa (ALERT) project addresses these two knowledge gaps and developed, implemented and evaluated a multi-faceted intervention to improve intrapartum care through a stepped-wedge cluster-randomized trial in 16 hospitals across 4 sub-Saharan African countries: Benin, Malawi, Tanzania and Uganda. The intervention was composed of (i) end-user participation of women, families and midwifery providers to co-design the intervention, (ii) in-service midwifery competency-based training, (iii) quality improvement, and (iv) empowerment and leadership mentoring of maternity unit leaders^32^ (Fig. 1). ALERT focused on proven evidence-based clinical interventions as well as the experience of care to improve the quality of care in a holistic manner and adapted to context. The intervention started with a co-design process which informed six training modules. The 3-day training sessions were supported by Laerdal Global Health MamaBirthie low-cost models^33^. Quality improvement used the Plan-Do-Study-Act cycles and promoted the documentation using quality improvement journals. Quarterly on-site mentoring and frequent phone calls with the maternity unit’s head supported the improvement work. The ALERT intervention targeted midwifery and clinical staff in the maternity wards of high-caseload hospitals^34^.

The main aim of this stepped-wedge cluster-randomized trial was to assess the effects of the ALERT intervention on the primary outcome of early perinatal mortality. We hypothesized that the intervention would reduce early perinatal mortality (stillbirths and neonatal deaths within 24 h) by 25% through synergistic action^35^. We assessed the intervention against standard care informed by national policies and characterized by resource constraints.

## Results

### Patient disposition

**Fig. 2:**
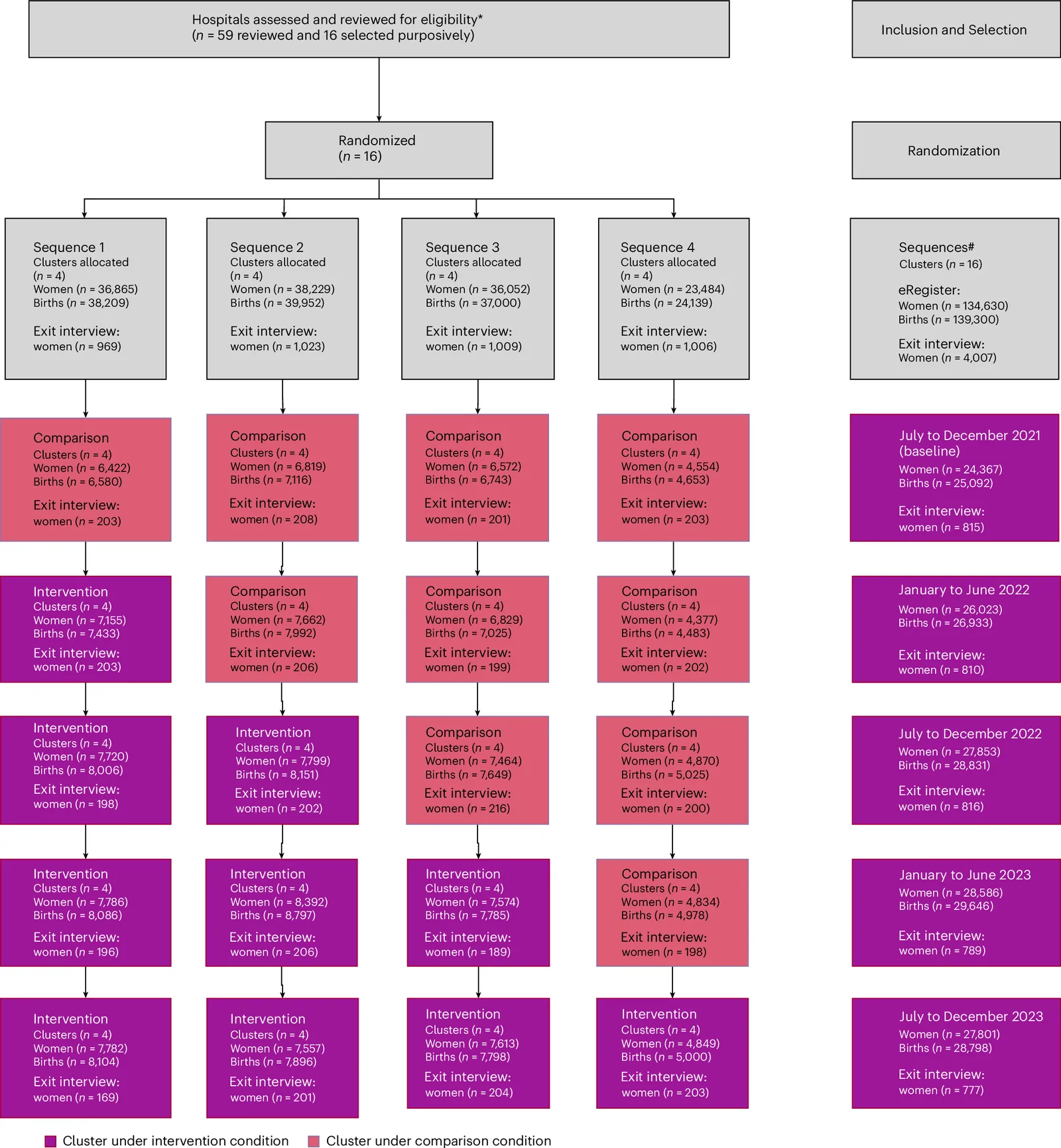
Trial diagram showing the women selected by hospital and trial period. *Trial hospitals were selected purposively in consultation with Ministries of Health to reflect public and private-not-for-profit hospitals in each country, with inclusion criteria notably: 2,500 births per year, availability of cesarean sections and rural location. ^#^Sequence periods represent: period 1 (1 Jul–31 Dec 2021), period 2 (1 Jan–30 Jun 2022), period 3 (1 Jul–31 Dec 2022), period 4 (1 Jan–30 Jun 2023) and period 5 (1 Jul–31 Dec 2023).

**Fig. 3:**
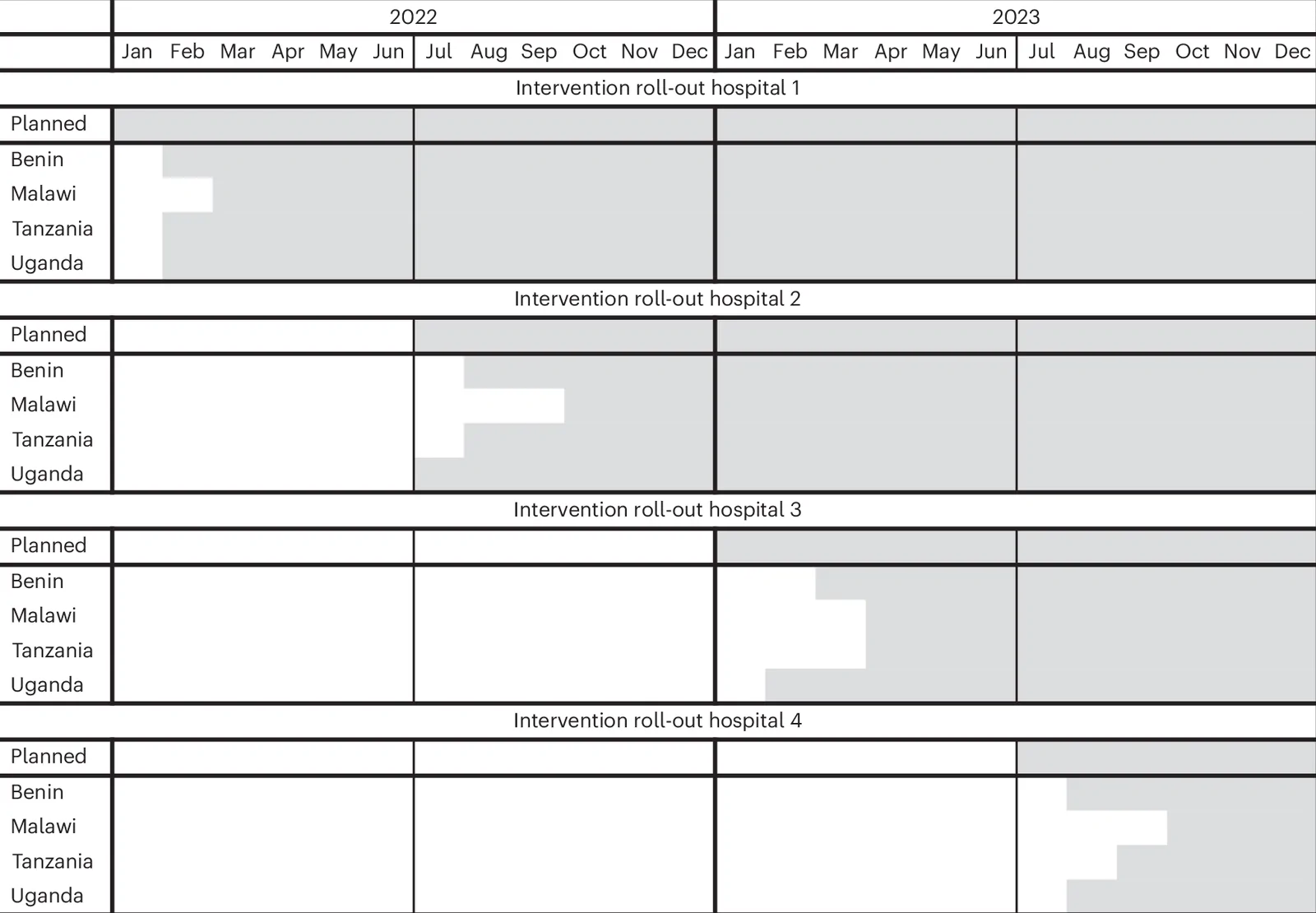
Intervention roll-out. The intervention was supposed to kickstart in January 2022 with the training in all hospital 1 facilities; however, training took place only in early February owing to operational delays, except Malawi, which was delayed to early March. In Malawi, the ethics renewal in July 2022 was delayed, which is why the training was further delayed for the second hospitals and then all subsequent hospitals.

**Extended Data Fig. 1:**
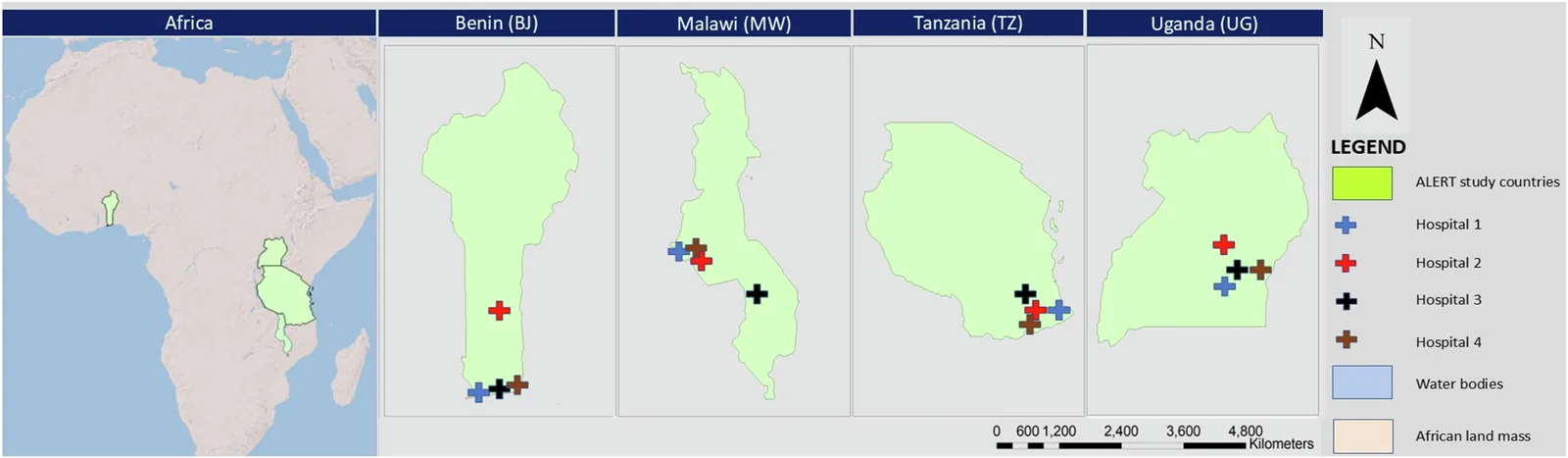
Map showing the ALERT trial countries and study hospitals. Country boundaries and longitude graticules are from the UIA Latitude/Longitude Graticules and World Countries Boundaries dataset (ArcGIS Hub, https://hub.arcgis.com/datasets/a21fdb46d23e4ef896f31475217cbb08); the map was produced in ArcGIS. Hospital locations are our own data, and the north arrow and scale bar are standard ArcGIS elements.

All 16 identified and randomized hospitals participated in the trial in line with the allocation produced and published on 17 June 2020 (Figs. 2 and 3, Extended Data Fig. 1 and Supplementary Table 1). We implemented five periods: 1 July to 31 December 2021 (baseline), 1 January to 30 June 2022, 1 July to 31 December 2022, 1 January to 30 June 2023 and 1 July to 31 December 2023. We included 134,630 women who gave birth to 139,300 neonates (July 2021 to December 2023) and 4,007 exit interviews to assess respectfulness and responsiveness. The stepped-wedge randomization design resulted in 60,403 births (45%) in comparison and 74,227 (55%) in intervention periods, showing a good balance across study arms and periods (Table 1 and Supplementary Table 2). Thirty percent of women were between 20 and 24 years old. Just under 40% were primipara. About a quarter (27.5% in the comparison and 22.8% in the intervention period) of the women arrived as referrals, 90% of whom were in active labor.

**Table 1:** Characteristics of the study population by comparison and intervention period (134,630 births, 16 hospitals, 1 July 2021 to 31 December 2023)

	Comparison period	Intervention period
	*N* = 60,403 (45%)	*N* = 74,227 (55%)
Maternal age in years		
<20	13,423 (22.2%)	15,689 (21.2%)
20–24	18,159 (30.1%)	22,397 (30.2%)
25–29	12,957 (21.5%)	16,300 (22.0%)
30–34	9,025 (14.9%)	11,280 (15.2%)
35+	6,777 (11.2%)	8,501 (11.4%)
Women referred into hospital	16,565 (27.5%)	16,895 (22.8%)
Stage of referral		
Pregnancy, not in labor	1,720 (10.4%)	1,671 (9.9%)
Labor	14,796 (89.3%)	15,199 (90.0%)
Referring facility		
Home/community	5,118 (30.9%)	2,949 (17.5%)
Lower-level facility	10,258 (61.9%)	12,880 (76.2%)
Other hospital	1,166 (7.0%)	1,058 (6.3%)
Gravidity, median (interquartile range)	2.0 (1.0, 4.0)	2.0 (1.0, 4.0)
Parity category		
Nullipara	24,252 (40.2%)	28,912 (39.0%)
1–2	22,333 (37.0%)	28,461 (38.3%)
3–4	10,314 (17.1%)	12,527 (16.9%)
5+	3,504 (5.8%)	4,327 (5.8%)
Previous cesarean section	7,652 (20.3%)	8,972 (18.9%)
Number of ANC visits, current pregnancy		
<4	23,231 (38.5%)	25,370 (34.2%)
4+	36,347 (60.2%)	48,575 (65.4%)
Multiple pregnancy	2,672 (4.4%)	2,737 (3.7%)
Maternal comorbidities		
Hypertensive disorders	3,678 (6.1%)	4,124 (5.6%)
Preterm labor	3,402 (5.6%)	3,743 (5.0%)
Augmentation of labor	1,181 (2.2%)	859 (1.3%)

Missing values: maternal age, 122 obs (<0.1%); referral, 74 obs (<0.1%); gravidity, 95 obs (<0.1%); number of antenatal care (ANC) visits, 1,107 observations (obs; 0.7%).

### Intervention and reach

The intervention was initiated with competency-based in-facility training delivered to each hospital per country in accordance with the trial design and randomization, with slight delays (Figs. 2 and 3). Training was delivered as short, flexible sessions timed around shift changes with reinforcement sessions on subsequent days. A total of 484 maternity providers, quality improvement focal persons and administrative staff received the initial training, exceeding the 414 staff members originally registered as working in the trial hospitals owing to staff rotation demanding re-training^36^. A detailed fidelity assessment carried out in the final year of the trial showed that the ALERT team conducted 7, 12, 1 and 9 mentoring visits to hospitals in Benin, Malawi, Tanzania and Uganda, respectively. With the exception of Tanzania, the number of visits exceeded the six quarterly on-site mentoring visits specified in the protocol. A total of 112 h, 284h , 175h and 83 h of mentoring were delivered on-site and via WhatsApp in Benin, Malawi, Tanzania and Uganda, respectively, corresponding to an average of 56 h of mentoring per quarter per hospital.

#### Primary outcomes

**Fig. 4:**
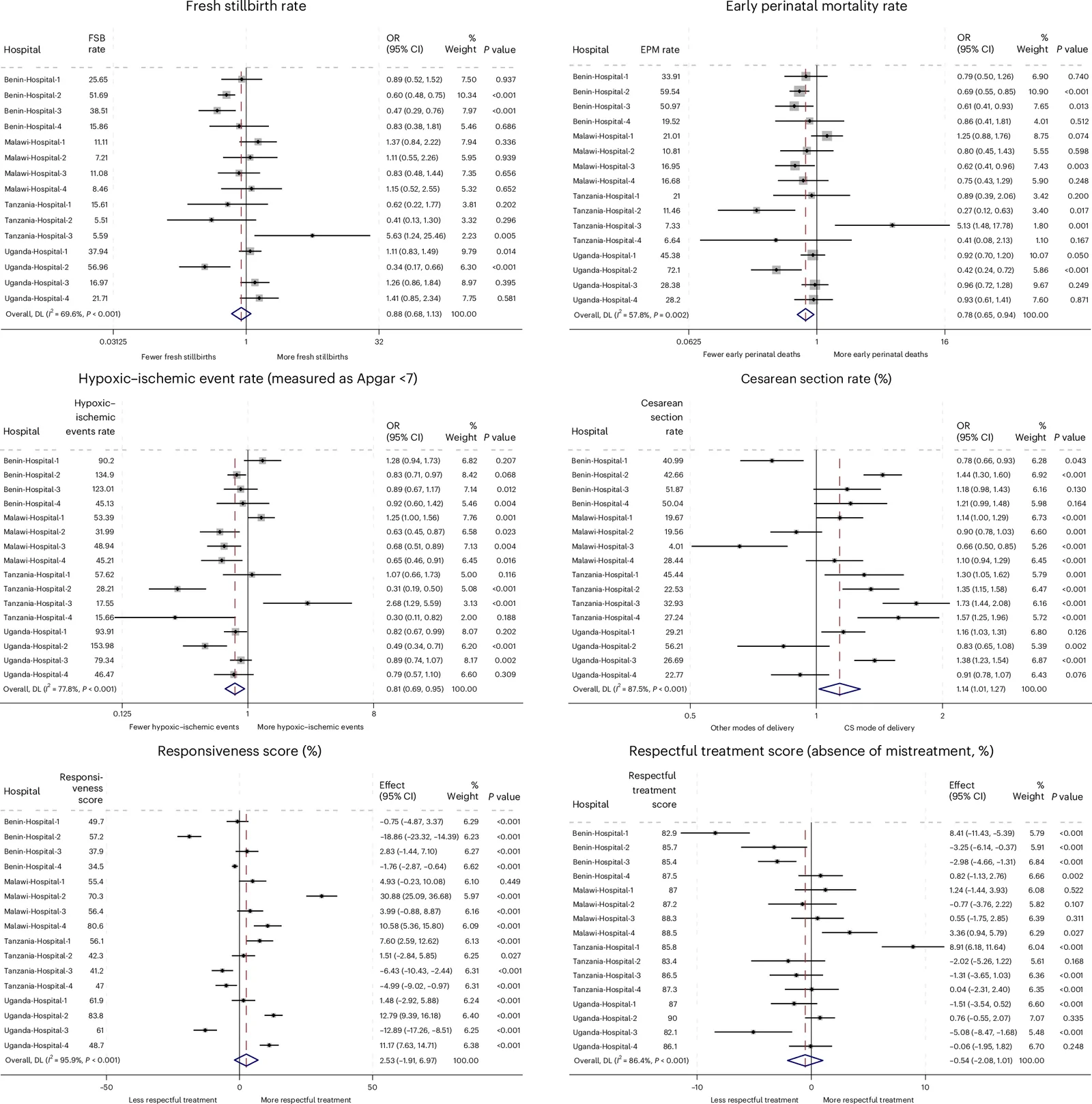
Effects of the intervention on primary and secondary outcomes. Each square is a hospital’s estimate of the intervention effect; its area is proportional to that hospital’s weight in the pooled analysis: an OR for fresh stillbirth, early perinatal mortality, hypoxic–ischemic events (Apgar <7) and cesarean section, and a mean difference for the responsiveness and respectful treatment scores. Horizontal lines are 95% CIs and the diamond is the pooled estimate with its 95% CI from a DerSimonian–Laird random-effects meta-analysis. The hospital is the unit of analysis; it is the unit of randomization and an independent cluster, not a technical replicate, and the women and neonates are observations nested within it. There were 16 hospitals (15 for fresh stillbirth, as Tanzania Hospital 4 recorded no fresh stillbirths and gave no estimable OR); the clinical outcomes are based on 139,300 neonates from 134,630 women and the two scores on 4,007 postpartum interviews. *I*^2^ and Cochran’s *Q* describe heterogeneity between hospitals. All tests were two-sided and no adjustment was made for multiple comparisons. Pooled results (two-sided *P*): fresh stillbirth (FSB) OR 0.88 (95% CI 0.68 to 1.13), *P* = 0.32; early perinatal mortality (EPM) 0.78 (0.65 to 0.94), *P* = 0.008; hypoxic–ischemic events 0.81 (0.69 to 0.95), *P* = 0.008; cesarean section (CS) 1.14 (1.01 to 1.27), *P* = 0.027; responsiveness 2.53 (−1.91 to 6.97), *P* = 0.26; respectful treatment −0.54 (−2.08 to 1.01), *P* = 0.49. DL, DerSimonian-Laird.

Early (24 h) perinatal mortality decreased by 22% (adjusted odds ratio (OR) 0.78; 95% confidence interval (CI) 0.65–0.94) (Table 2 and Fig. 4). Fresh stillbirths showed a non-significant effect (adjusted OR 0.88; 95% CI 0.68–1.13).

**Table 2:** Effect of the intervention on primary and secondary outcomes and process indicators (134,630 women, 139,300 neonates, 4,007 interviews with women postpartum, 16 hospitals in Benin, Malawi, Tanzania and Uganda)

Indicator	Level of analysis	*N*	Missing values	Adjusted OR (95% CI)/Coef (95% CI)*	Source
Primary outcomes					
Fresh stillbirth rate	Neonates	139,300	0	0.88 (0.68–1.13)	e-registry
Early perinatal mortality rate	Neonates	139,300	0	0.78 (0.65–0.94)	e-registry
Secondary outcomes					
Hypoxic–ischemic event	Neonates	139,300	0	0.81 (0.69–0.95)	e-registry
Neonatal seizures	Neonates	139,300	0	0.79 (0.55–1.15)	e-registry
Cesarean section	Neonates	139,300	0	1.14 (1.01–1.27)	e-registry
Severe maternal morbidity	Woman	134,630	0	0.83 (0.61–1.13)	e-registry
Responsiveness (% score)	Woman	4,007	0	2.53 (−1.91 to 6.97)*	Exit interview
Respectful treatment (% score)	Woman	4,007	0	−0.54 (−2.08 to 1.01)*	Exit interview
Process indicators					
Admission standards and procedures					
Fetal heart check on admission	Woman	134,630	0	25.05 (9.20–68.23)	e-registry
Intrapartum monitoring					
Fetal monitoring first stage of labor	Woman	134,630	0	2.05 (1.11–3.79)	e-registry
Detection of fetal distress	Neonates	139,300	98	0.54 (0.38–0.76)	e-registry
Emergency preparedness					
Resuscitation for neonates with Apgar <7	Neonates	9,371	0	1.13 (0.82–1.55)	e-registry
Responsiveness-focused					
Companion present during labor/birth	Woman	4,006	1	1.55 (0.57–4.19)	Exit interview
Early newborn care					
Breastfeeding within 1 h	Woman	3,836	171	1.22 (0.72–2.05)	Exit interview

*Coefficient based on linear mixed-effects model.

### Secondary outcomes

Hypoxic–ischemic events decreased by 19% in the intervention compared with the comparison period (adjusted OR 0.81; 95% CI 0.69–0.95). Further, we observed no significant effect on neonatal seizures (adjusted OR 0.79; 95% CI 0.55–1.15).

We observed a 14% increase in the odds of cesarean sections (adjusted OR 1.14; 95% CI 1.01–1.27), from 27.4% to 29.4% between comparison and intervention periods. We observed some associations with severe maternal morbidity (adjusted OR 0.83; 95% CI 0.61–1.13). Neither responsiveness nor respectful treatment (absence of mistreatment) scores changed significantly (coefficients 2.53 and −0.54, respectively) (Table 2 and Fig. 4).

### Process outcomes

Process indicators, reflecting the intervention priorities, revealed that fetal heart monitoring on admission typically done using the Pinard stethoscope increased from 89% to 95% coverage (OR 25.05; 95% CI 9.20–68.23) (Table 2 and Supplementary Table 3). Documentation of ongoing fetal monitoring during labor improved (OR 2.05; 95% CI 1.11–3.79), while detection of fetal distress did not improve (OR 0.54; 95% CI 0.38–0.76) and neonatal resuscitation practices increased but not statistically significantly (OR 1.13; 95% CI 0.82–1.55).

We observed substantial heterogeneity between hospitals with *I*^2^ values exceeding 50% for most outcomes, supporting our decision to estimate hospital-specific effects before pooling results. Despite this variability, the direction of effect was broadly consistent: 13 of 16 hospitals showed reductions in early perinatal mortality, although individual estimates were often not statistically significant, reflecting limited power at the hospital level. However, distinct country-level patterns were seen. In Tanzania, early perinatal mortality rate declined from 15 to 9 per 1,000 births (hospitals 1 and 2), while hospital 3 showed an increase. In Uganda, hospital 2 saw a strong decline, while hospitals 1 and 4 had smaller gains while hospital 3 remained largely unchanged. Benin and Malawi showed more modest and variable improvements (Table 2 and Fig. 4).

#### Safety

Our adverse event monitoring as part of the process evaluation indicated no safety concerns in regard to the training and quality improvement. The 14% increase in the odds of cesarean sections was within expected ranges, supporting the safety of the intervention.

### Post hoc analysis

#### Sensitivity, per-protocol analysis and temporal trends

**Extended Data Fig. 2:**
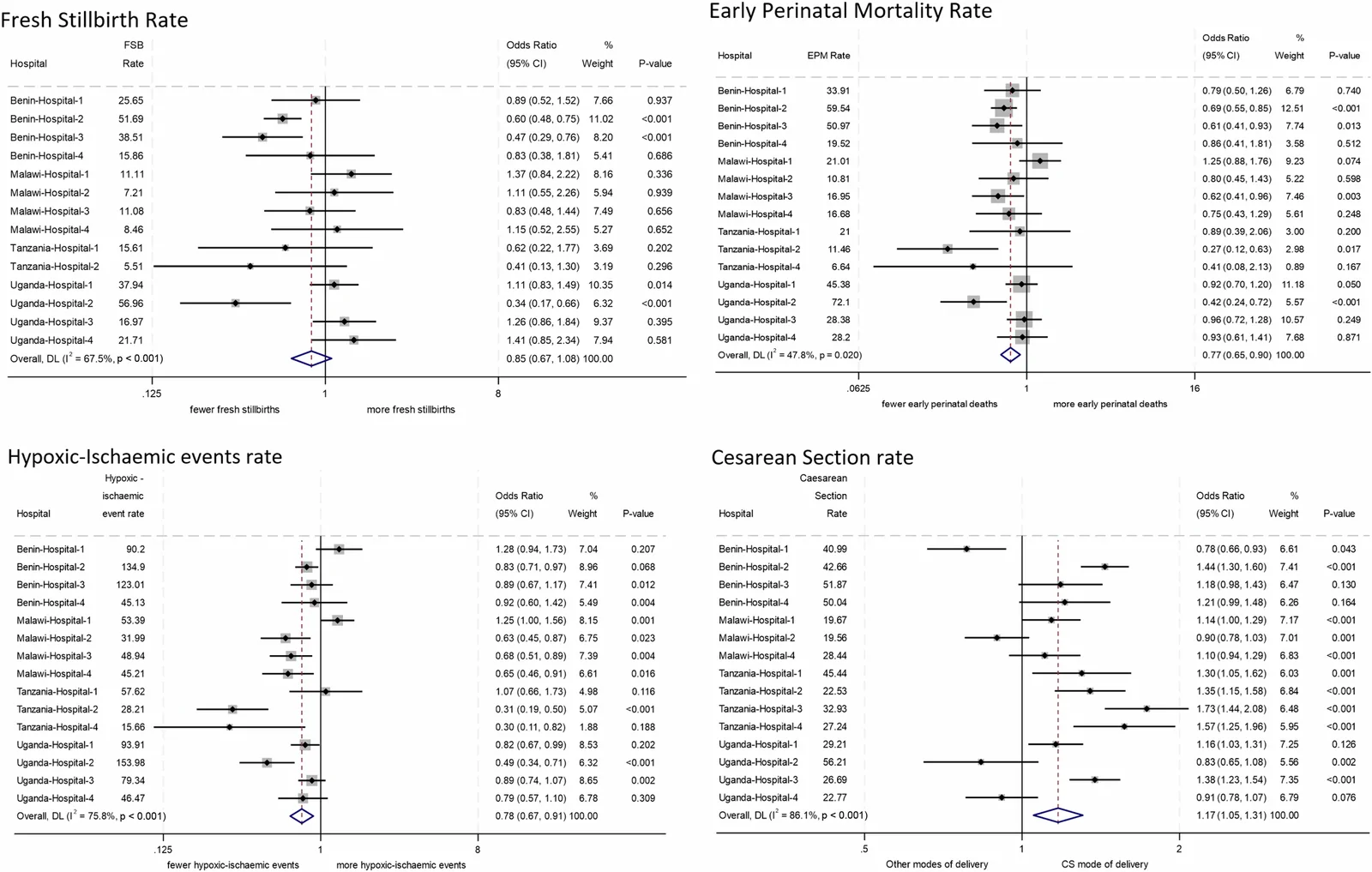
Sensitivity analysis indicator (exclusion of hospitals with data quality concerns). This repeats the Fig. 4 analysis but excludes, for each outcome, the hospital identified as a data-quality outlier: Tanzania Hospital 3 for fresh stillbirth, early perinatal mortality and hypoxic–ischemic events, and Malawi Hospital 3 for cesarean section. Squares are the hospital ORs (area proportional to weight), horizontal lines are 95% CIs, and the diamond is the pooled DerSimonian–Laird random-effects OR with its 95% CI. The hospital is the unit of analysis, an independent cluster and the unit of randomization, not a technical replicate. *n* = 14 hospitals for fresh stillbirth and *n* = 15 for each of the other three outcomes. *I*^2^ and Cochran’s *Q* describe heterogeneity; all tests were two-sided and no adjustment was made for multiple comparisons. Pooled results: fresh stillbirth OR 0.85 (95% CI 0.67 to 1.08), *P* = 0.18; early perinatal mortality 0.77 (0.65 to 0.90), *P* = 0.001; hypoxic–ischemic events 0.78 (0.67 to 0.91), *P* = 0.001; cesarean section 1.17 (1.05 to 1.31), *P* = 0.004.

**Extended Data Fig. 3:**
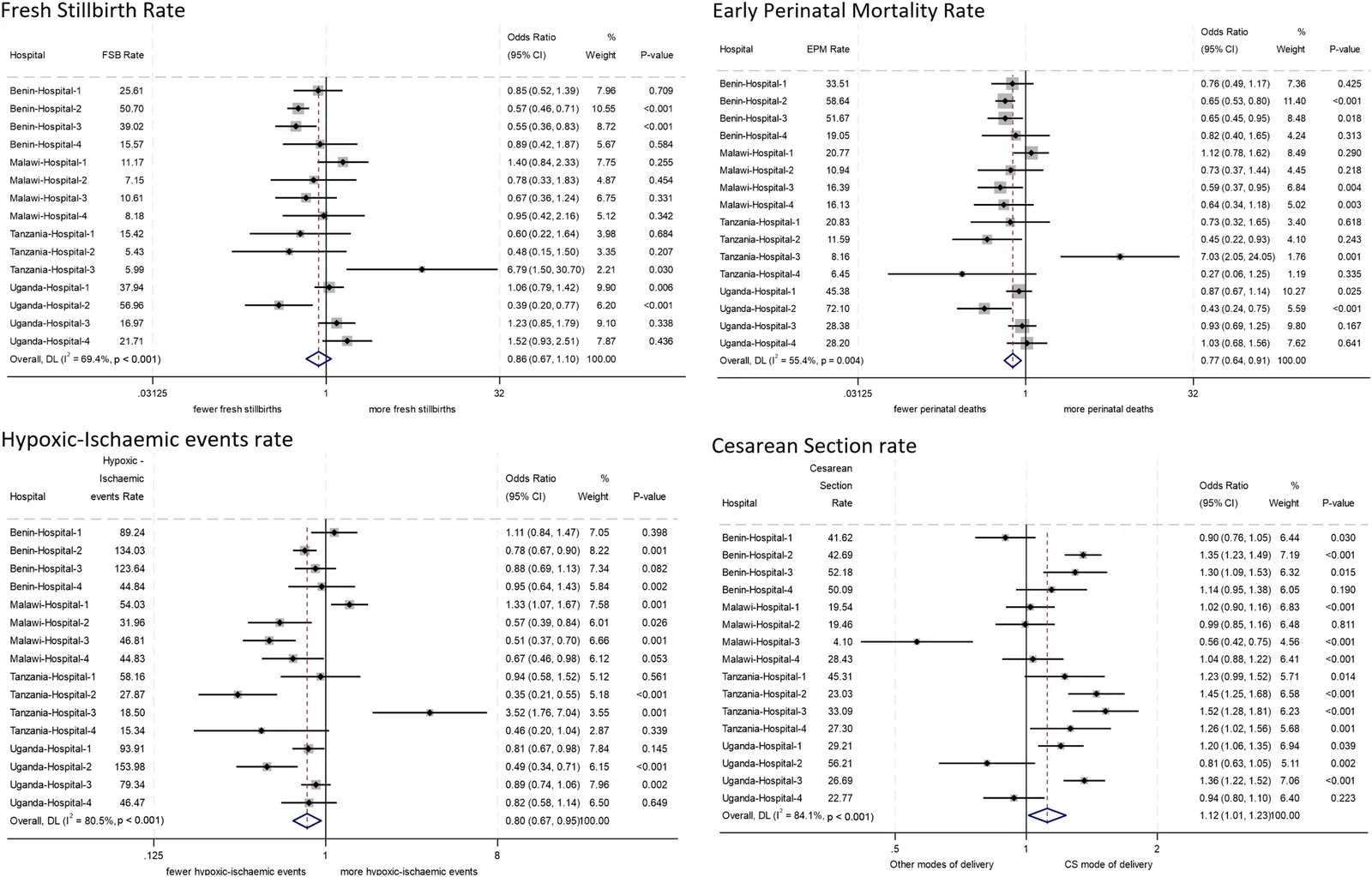
Per-protocol analysis using effective implementation periods. This repeats the Fig. 4 analysis under a per-protocol definition of exposure, in which a hospital counts as intervention only once the intervention was fully implemented and the transition period is excluded. Squares are the hospital ORs (area proportional to weight), horizontal lines are 95% CIs, and the diamond is the pooled DerSimonian–Laird random-effects OR with its 95% CI. The hospital is the unit of analysis, an independent cluster and the unit of randomization, not a technical replicate. *n* = 15 hospitals for fresh stillbirth and *n* = 16 for each of the other three outcomes. *I*^2^ and Cochran’s *Q* describe heterogeneity; all tests were two-sided and no adjustment was made for multiple comparisons. Pooled results: fresh stillbirth OR 0.86 (95% CI 0.67 to 1.10), *P* = 0.23; early perinatal mortality 0.77 (0.64 to 0.91), *P* = 0.004; hypoxic–ischemic events 0.80 (0.67 to 0.95), *P* = 0.012; cesarean section 1.12 (1.01 to 1.23), *P* = 0.024.

We performed a sensitivity analysis excluding three hospitals following discussions of preliminary results with country teams in December 2024, which identified three hospitals (Tanzania Hospitals 3 and 4 and Malawi Hospital 3) as outliers owing to implementation barriers including limited staff engagement and data quality concerns. For example, hospital 3 in Malawi was a community hospital with severe resource constraints. Hospital 3 in Tanzania was part of another project where teams had to report during birth on process of labor, which we believe led to underreporting during the pre-intervention period and limited engagement in the trial because of fear of blame and disciplinary consequences. Details are found in the Supplementary Information (Extended Data Fig. 2 and Supplementary Table 4). The analysis excluding these hospitals indicated results consistent with the main analysis. Per-protocol analysis was also consistent with the main analysis. The OR for fresh stillbirth was 0.86 (95% CI 0.67–1.10); for early perinatal mortality 0.77 (95% CI 0.64–0.91); hypoxic–ischemic events (OR 0.80; 95% CI 0.67–0.95) and cesarean section (OR 1.12; 95% CI 1.01–1.23) (Extended Data Fig. 3).

Three pre-specified sensitivity analyses requested during peer review (Hartung–Knapp–Sidik–Jonkman (HKSJ) pooling, per-protocol restricted to hospital-periods with documented full implementation, and country-stratified random-effects meta-analysis) gave results consistent with the primary analysis. For the primary outcome of early perinatal mortality, there was no evidence of between-country heterogeneity of effect (*Q*ᵇ(3) = 1.61, *P* = 0.66). Full descriptions and outputs are provided in Supplementary Tables 5–7.

The primary intention-to-treat analysis retained all 16 hospitals. The three-hospital exclusion was post hoc, triggered by pre-specified data-quality monitoring indicators combined with structured contextual review with country teams in December 2024 (criteria in Supplementary Table 4).

## Discussion

In this stepped-wedge cluster-randomized trial in 16 high-caseload hospitals in Benin, Malawi, Tanzania and Uganda, we developed, implemented and evaluated a multi-faceted intervention of co-design, training, quality improvement and mentorship. The intervention was associated with 22% lower odds of the composite outcome in-facility early perinatal mortality, 19% lower odds of hypoxic–ischemic events and a 14% increase in cesarean sections. Mortality reductions were likely underpinned by improved processes such as increased fetal heart rate monitoring and cesarean sections. We did not observe any effects on responsiveness or respectful care.

Our results suggesting 22% lower odds of in-facility early perinatal mortality concur with larger evaluations done in similar settings. The Preterm Birth Initiative in Kenya and Uganda reported 34% reduced odds of mortality in preterm neonates in response to a comprehensive quality improvement strategy^20^. Positive effects of interventions combining training, quality improvement and mentorship to improve intrapartum and early neonatal care are described by the Nepal Perinatal Quality Improvement study^21,37,38^, and the hospital initiative in the Democratic Republic of Congo^39^. Together, these findings reinforce the importance of multi-component strategies that address skills, systems and leadership simultaneously to achieve meaningful and sustained improvements in evidence-based practice and mortality, even if they are potentially resource intensive, as raised by others^40^. However, our economic analysis suggested limited costs of our approach^41^. For example, our co-design approach was not implemented in all hospitals and the mentoring approach relied largely on digital communication. In summary, our results support the importance of combining strategies to obtain greater changes in evidence-based practices and mortality^28,42^, while initiatives relying on quality improvement or training alone are increasingly recognized as insufficient to produce comparable mortality gains in resource-constrained settings^20,43,44^.

Our results also highlight the potential of routine intrapartum care as opposed to addressing emergency obstetric care as previous training and improvement packages have done^17–19^. Furthermore, our reduction in perinatal mortality and morbidity was achieved without technological innovations^45^. This highlights the potential effects of strengthening routine intrapartum care, that is, preventive and promotive midwifery care—an important finding towards the programming of the Every Woman Every Newborn Everywhere (EWENE) initiative^24^.

Our intervention adopted a health system strengthening lens targeting recurrent, cross-cutting challenges in intrapartum care^46^. Through co-design, the ALERT team and maternity care providers jointly identified key quality-of-care deficits, their underlying causes and how these were shaped by local healthcare constraints including providers’ competences and attitudes^47^. For example, co-design interviews with women postpartum highlighted that women missed important information on labor progress and why decisions were made^48^. Furthermore, hierarchical structures and power imbalances between women and providers led to breakdowns in communications^49^. These findings led to developing specific training components on communication and teamwork. We believe the co-design process created an empowering working relationship and traction for change (M.S., manuscript in preparation). The competency-based training reinforced essential intrapartum practices and translated co-designed solutions into routine clinical behaviors^36^.

We recognize that the positive effect on mortality was accompanied by an increase in cesarean section, which we interpret as a positive health systems strengthening effect. Population-based cesarean section rates in the included countries are at or below 10%, thus at levels where we would not expect an overuse of cesarean section^50,51^. Still, in low-resource hospital settings such as the ones in this study, cesarean section alone does not guarantee improved survival, particularly when delays in recognition or referral persist^52,53^. However, when considered alongside reductions in early perinatal mortality and hypoxic–ischemic events, the increase in cesarean section likely reflects more timely identification of complications and appropriate escalation of care. We therefore interpret this pattern as consistent with improved quality of intrapartum care rather than harmful overuse^52^.

Our intervention showed no effect on responsiveness or on respectful care, although the teams worked intensively to improve responsiveness, including companionship. Several possible reasons may explain this. First, we cannot exclude the possibility that our tools were not sensitive enough to capture change. Several studies have indicated that exit interviews before discharge are likely to underreport abuse and mistreatment, and this could have led to the fact that we could not report an effect^54,55^. Second, interpersonal skills, attitudes and behaviors might need more time before sustained changes can be expected. Third, policy and structural constraints made it difficult to introduce companionship^56^. Together, these factors may explain the absence of measurable effects on responsiveness and respectful care within the trial period.

The strength of this multi-country trial is the use of the stepped-wedge intervention design, a large number of births, and the inclusion of public and faith-based hospitals in four countries, improving generalizability. The use of the stepped-wedge trial design meant that all hospitals received the intervention, which enhanced engagement. Another strength was that while we adhered to structured processes, the outlined implementation strategy and a standardized curriculum, co-design, mentoring and quality improvement addressed local constraints in a flexible manner, thus arriving at a context-adapted, co-designed intervention with relevance to local context. We kickstarted our intervention using co-design methodologies, which allowed providers to influence the intervention and targeted routine intrapartum care, underscoring the potential of improving basic childbirth and preventive midwifery care as opposed to specifically addressing selected emergency obstetric conditions^45,57^.

Our analytical approach had several strengths. First, we used hospital-specific mixed-effects models, rather than a single pooled analysis, acknowledging heterogeneity, confirmed by *I*^2^ values exceeding 50%. This avoided the ecological fallacy of assuming uniform intervention effects across settings. Second, the random-effects meta-analysis preserved this heterogeneity while providing overall estimates. Forest plots transparently displayed both individual hospital effects and uncertainty. Third, the intention-to-treat framework maintained the benefits of randomization, while sensitivity analyses (excluding sites with data quality concerns and per-protocol analysis) tested the robustness of our findings. Fourth, we had minimal missing data, reducing selection bias.

Our study faces limitations, one of which was the start of our trial in 2020 during the COVID-19 pandemic, which precluded face-to-face meetings during the critical co-design and start-up period. The trial started with a 6-month delay, limiting the financial space. One consequence was that we were unable to introduce the lactate testing in a sub-sample.

Our two primary and four of six secondary outcomes were assessed using an innovative e-registry data collection tool supported by rigorous quality assurance. Still, we cannot fully exclude underreporting of perinatal events, which is why we included a sensitivity analysis. National accountability measures, as applied in Tanzania, disincentivized maternity providers from reporting deaths for fear of repercussions^58^. Furthermore, we were not able to follow up neonates for longer than 24 h after childbirth, thus beyond discharge, to establish neonatal mortality. While we achieved extremely low missing data (<1% for primary outcomes), we acknowledge that in high-volume settings, emphasis on completeness could potentially affect accuracy. We note that the 4-month pre-intervention data collection phase supported the attained completeness rates of the primary and secondary outcomes^59^. However, we were unable to ensure the consistent documentation of some processes during labor, although planned. In consequence, we had over 90% missing data for the process measure on decision-to-birth time for cesarean section. Our data quality assurance focused on the primary and secondary outcomes.

We used a composite indicator to account for misclassification between fresh stillbirth and very early neonatal deaths. Our study was not powered to indicate a 20%, but only a 30% reduction of the fresh stillbirth rate, which led to imprecise estimates. Similarly, other estimates had large CIs, pointing to the fact that the trial had limited power as adverse perinatal events are fortunately rare. We analyzed the cesarean section rate at two time points together with our data safety and monitoring board as a strong increase would have pointed to a negative effect, but had no other pre-defined stopping rules in view of the type of intervention. No alpha-spending procedures were used, and no trial decisions were based on interim statistical testing.

The DerSimonian–Laird method, while standard, can underestimate uncertainty when combining results from relatively few clusters. We made no adjustment for multiple testing despite examining numerous secondary outcomes and process indicators, increasing the risk of Type I errors. The stepped-wedge design with its inherent temporal trends poses analytical challenges; while our models included time period as a fixed effect, residual confounding from unmeasured time-varying factors remains possible. Finally, hospital-level randomization, while pragmatic, limited our ability to adjust for potential imbalances in women-level characteristics that may have occurred despite randomization. We pre-specified only two primary outcomes and did not apply a formal multiplicity correction; secondary and process outcomes should therefore be interpreted on consistency of direction rather than on individual *P* values. Neonates delivered as multiples (5,409 women, 4.0% of deliveries) were treated as independent observations in the hospital-specific models; given the low multiple-birth prevalence and the consistent direction of effect shown in the pre-specified sensitivity analyses (Supplementary Tables 5–7), we expect any residual Type I error inflation from unmodelled within-mother clustering to be small.

Our multi-centered and multi-country stepped-wedge, cluster-randomized trial suggests that the multi-faceted ALERT intervention reduced perinatal mortality and morbidity. The effects were strongest for early perinatal mortality, but consistent across the indicators of morbidity and mortality. Our economic analysis estimated incremental costs per perinatal death averted of US$805, US$2,651, US$3,188 and US$760 in Benin, Malawi, Tanzania and Uganda, respectively, based on post-implementation perinatal mortality rates, with travel costs representing the largest cost component^41^.

The process evaluation confirmed the synergistic effects across the four intervention components. Co-design was reported to have an empowering effect, to enhance the relevance of the training, to reveal previously untapped resources and to improve coordination among the different actors involved in hospital quality improvement activities. Competency-based training was considered valuable both for refreshing clinical skills and for prompting reflection on team interaction and communication. Quality improvement and leadership mentoring supported the identification of local solutions to a broader range of barriers to implementing evidence-based interventions^60^. Taken together, these findings suggest that the intervention has potential for scalability. It was perceived as relevant and contextually aligned, demonstrated effectiveness and incurred limited costs^61^. Our approach to using co-design only in selected facilities and providing a mentoring follow-up using WhatsApp further increased feasibility and cost-effectiveness.

In summary, the findings highlight the potential of strengthening routine intrapartum care, thus preventive and promotive midwifery care. Our model, including co-design and strengthening midwifery competences and leadership, may serve as a model to complement the ongoing large-scale work on neonatal care within EWENE. Our trial confirms the relevance of combining interventions with a synergistic effect.

## Methods

### Trial design and oversight

ALERT was a stepped-wedge cluster-randomized trial with five 6-month periods, implemented by the ALERT Consortium in four hospitals in each of Benin, Malawi, Tanzania and Uganda^32^. The cluster (hospital) design was chosen as the intervention was delivered at the hospital level, targeting providers in the maternity ward. A stepped-wedge design was intended to mirror scale-up for policy buy-in and for statistical efficiency as we expected larger cluster-level differences^62^. ALERT is registered (17 June 2020) in the Pan African Clinical Trials Registry at 202006793783148. The first and second authors, the principal investigators and the trial statistician, respectively, attest to the accuracy and completeness of the data and the adherence to the study protocol. Trial oversight was provided by an independent data monitoring and safety board.

### Ethics statement

The ALERT study was approved by the Swedish Ethical Authority and in each participating country: Sweden (Etikprövningsmyndigheten—Dnr 2020-01587), Benin (Comité National d’Ethique pour la Recherche en Santé 068/MS/DRFMT/CNERS/SA), Malawi (College of Medicine Research and Ethics Committee P.04/20/3038), Tanzania (National Institute of Medical Research Tanzania/HQ/R.8a/Vol.IX/3493 & Muhimbili University of Health and Allied Sciences REC-4-2020-118 & Aga Khan University Ethical Review Committee ERC, EA/2019/044/fb), Uganda (Uganda National Council for Science and Technology HS1324ES) and Belgium (Institute of Tropical Medicine Antwerp and The Ethics Committee at the University Hospital Antwerp 1375/20. B3002020000116).

The perinatal e-registry was exempted from individual consent from women and their fetuses, as only routinely documented and abstracted data were used in the ALERT perinatal e-registry, and data were de-identified. To assess respectful and responsive care, we performed exit interviews and received individual informed consent from each participating woman in the postpartum period.

### Ethics and inclusion statement

This project was conceived and designed through long-standing partnerships between Makerere University (Uganda), Kamuzu University of Health Sciences (Malawi), London School of Hygiene and Tropical Medicine (UK), Karolinska Institutet (Sweden), Centre de Recherche en Reproduction Humaine et en Démographie (Benin), Institute of Tropical Medicine (Belgium), Aga Khan University (Tanzania) and Muhimbili University of Health and Allied Sciences (Tanzania). Country teams in Benin, Malawi, Tanzania and Uganda actively drove the grant application, study design, study implementation, data ownership, intellectual property and authorship of publications to ensure that this project was locally relevant. Furthermore, the ALERT intervention was developed using a co-design process with end-users including women, families and midwifery providers from each country.

Roles and responsibilities were agreed upon among collaborators ahead of the research, and capacity-building plans for local researchers were implemented, including training of PhD students. As outlined in the Ethics statement, the ALERT study was approved by a local ethics review committee in each participating country. We have cited several local and regional research relevant to the ALERT study.

### Settings, participating hospitals and participants

The four countries were purposively selected as they reflect different health system contexts and leveraged pre-existing research collaborations (Extended Data Fig. 1 and Supplementary Table 2). Details on recruitment and setting are published elsewhere^32,63,64^. The intervention was implemented in 16 hospitals (3 public and 1 faith-based per country)^32,47^. Aspects of variations in midwifery education^65^ and knowledge and skills to provide quality intrapartum and neonatal care are presented elsewhere^36^. The hospitals faced human resource limitations and insufficient equipment and supplies.

We included all women who gave birth to a live or stillborn infant >1,000 g between 1 July 2021 and 31 December 2023 in an intention-to-treat analysis^66^. We expanded the inclusion period in Benin, Malawi and Tanzania to February 2024 because of implementation delays in our per-protocol analysis (Fig. 2 and Supplementary Information 9, Statistical Analysis Plan). We excluded mother-baby pairs that arrived at the hospitals after giving birth, unless one of multiples was born in the participating hospital.

### Intervention

The ALERT intervention focused on the quality of intrapartum care targeting maternity providers in the included hospitals. We developed the intervention using a co-design process complemented by facility and knowledge assessments^48,49,67^ (January and September 2021) (Fig. 1). Training materials were developed addressing knowledge deficits and implementation challenges^17,68^. The implementation started with a 3-day training^33^, during which locally prioritized improvement topics were selected. Subsequently, maternity teams implemented and evaluated improvements under mentorship from ALERT researchers. The focus on mortality and experience of care was thought to be complementary. Responsiveness offers an opportunity to adapt care to changing women’s needs, promote women’s access to effective interventions and improve the quality of services^34^.

### Outcomes

The trial’s primary outcomes were in-facility (fresh) stillbirths defined as the number of fresh stillbirths of at least 1,000 g per 1,000 live- and stillbirths and a composite early perinatal mortality indicator defined as in-facility (fresh) stillbirths and 24 h neonatal mortality defined as death of a neonate within 24 h after birth per 1,000 live- and stillbirths, both assessed using our perinatal e-registry^32^. Secondary outcomes were hypoxic–ischemic events defined as a neonate with Apgar <7 at 5 min, neonatal seizures, cesarean sections, severe maternal morbidities defined as women with hysterectomy, laparotomy, blood transfusion, admission to an intensive care unit or referral to a higher-level facility, all expressed per 1,000 live- and stillbirths using the perinatal e-registry. Our respectful-care-focused secondary outcomes were responsiveness and respectful treatment (1 minus mistreatment). They were assessed using exit interviews with women after having given birth. Process measures were not pre-specified owing to the co-design of the intervention leading to adapted training and intervention components in the development phase. We report on fetal heart rate check at admission, fetal monitoring during the first stage of labor, detection of fetal distress, resuscitation of babies, companionship and breastfeeding within 1 h of birth. Details of the process indicators are listed in Supplementary Table 8. Cost-effectiveness and implementation process findings will be reported elsewhere^35^.

### Sample size

We used a sample size formula for stepped-wedge trials^32,69^. The study was sufficiently powered (75–80%) to detect a 25% reduction in the outcome perinatal mortality with baseline rates between 1.4% and 2.0% and a 30% reduction in intrapartum stillbirth assuming a baseline rate of 1% and 95% CIs. Details are available in the supplementary file of the protocol^32^.

### Data collection

Data from patient files, birth registers and case notes were entered in a preprogrammed e-registry application in the Research Electronic Data Capture (REDCap) platform^59^. Data collectors underwent a 2-day training in March 2021, after which a 4-month pilot data collection phase started. Each tablet had a personalized token and password; data were uploaded daily. Data quality assurance included (1) automated data validation and logical checks in REDCap, (2) bi-weekly audits by running pre-prepared Stata do-files, and (3) cross-checking of entries against paper records.

To assess experience of care, we conducted exit interviews using a questionnaire adapted from established responsiveness and mistreatment questionnaires^70,71^. We performed an exploratory and confirmatory factor analysis, which identified six factors: ‘Communication & supportive care’, ‘Hospital environment’, ‘Maintained respect & dignity’, ‘Social support’, ‘Maintained privacy & confidentiality’ and ‘Lack of physical & verbal abuse’. Findings were summarized in two summative scores to construct a responsiveness and respectful care (1 minus mistreatment) score^72^. In line with the stepped-wedge trial, we included a sample of 50 women per hospital recruited into the perinatal e-registry at five time periods. Data collectors applied a randomization factor to recruit the target number in each hospital based on the number of eligible women being discharged from the study hospitals^73^.

### Selection of hospitals and randomization

Hospitals were purposively selected in March 2020, drawing from a national facility list to identify high-caseload hospitals. Criteria included (i) minimum caseload of 2,500 births per year; (ii) availability of cesarean section and blood transfusion; (iii) preferably located in rural districts; (iv) consisting of a mix of typical public and (faith-based) hospitals; and (v) interest in collaboration and no other ongoing initiative focusing on intrapartum care^32^. The final selection was done in consultation with the Ministry of Health and took into account feasibility concerns in terms of travel time between the hospitals to ensure operational efficiency.

Randomization using Stata and a pre-prepared code was carried out on 17 June 2020 by an independent statistician after obtaining consent from the participating hospitals (Supplementary Information Statistical Analysis Plan). Stratification by country was used to enable random allocation of the four hospitals. Maternity providers were not blinded to the intervention, and women and their families receiving care at these hospitals were aware of the quality improvement intervention.

### Safety monitoring

Safety oversight was provided by the independent Data Safety Monitoring Board. The preliminary Statistical Analysis Plan stated the development of clear stopping rules at approximately 50% and 75% of the planned sample size using pre-specified stopping criteria. However, in consultation with the Data Safety Monitoring Board, we agreed that clear stopping rules are difficult to apply for a non-clinical intervention such as training and quality improvement, which is universally perceived as valuable.

We monitored the safety of our approach by analyzing and discussing changes in the cesarean section rates during meetings with our Data and Safety Monitoring Board. Our main outcomes were maternal and neonatal events constituting safety measures. In addition, we monitored, as part of our process evaluation, any severe events happening in the hospitals such as disruptions owing to climate or political events, staff rotation, interruptions in supplies, allegations owing to severe events in the maternity and similar. Our team followed a debriefing protocol where such events were discussed within the ALERT team to support the mentors in acting appropriately.

### Statistical analysis

We used an intention-to-treat approach, analyzing all data at woman- and baby-level within the 16 hospitals according to their assigned intervention status. The 30-month study period was divided into 5 discrete 6-month intervals (Fig. 2 and Supplementary Information 9, Statistical Analysis Plan).

Descriptive analyses using frequencies and percentages for categorical variables, and median and interquartile ranges for continuous variables were conducted. Hospital-specific mixed-effects regression models were fitted to account for heterogeneity. We first considered generalized estimating equations adjusting for clusters and for the small sample^32^. However, in view of the heterogeneity, this statistical approach was not implementable.

Two primary outcomes were pre-specified. No formal adjustment for multiplicity was planned or applied because both outcomes were considered clinically important and the analyses followed the pre-specified statistical analysis plan. The two outcomes are closely related, with fresh stillbirth forming part of the composite perinatal mortality outcome.

For binary outcomes (primary and composite outcome) as well as the binary secondary outcomes, we used logistic mixed-effects models with random intercepts for intervention periods and presented ORs (95% CI). For continuous outcomes (responsiveness and respectful treatment scores), we used linear mixed-effects models and presented mean differences (95% CI). To account for time period effects and the stepped-wedge design structure, all models included the treatment status (intervention versus comparison) and time period (6-month intervals). Random intercepts were used for sequential randomization groups to account for clustering.

Hospital-specific effects were subsequently pooled using random-effects meta-analysis with the DerSimonian–Laird method and displayed as forest plots. Heterogeneity was assessed using *I*^2^ statistics, with values above 50% indicating substantial heterogeneity. We conducted sensitivity analyses by excluding hospitals identified as outliers based on both statistical and contextual considerations. Following discussions of preliminary results with country teams in December 2024, three hospitals (Tanzania Hospitals 3 and 4 and Malawi Hospital 3) were identified as outliers owing to fundamental implementation barriers, including lack of fetal monitoring capacity, data quality and limited staff engagement (Supplementary Table 4).

### Per-protocol analysis

We also conducted a per-protocol analysis using an alternative definition of the intervention period to ensure full implementation fidelity (Extended Data Fig. 3). Secondary outcomes and process indicators are presented without adjustment for multiple testing, as pre-specified in the Statistical Analysis Plan; they should be interpreted on consistency of direction and magnitude rather than on individual *P* values.

### Temporal trends

**Extended Data Fig. 4:**
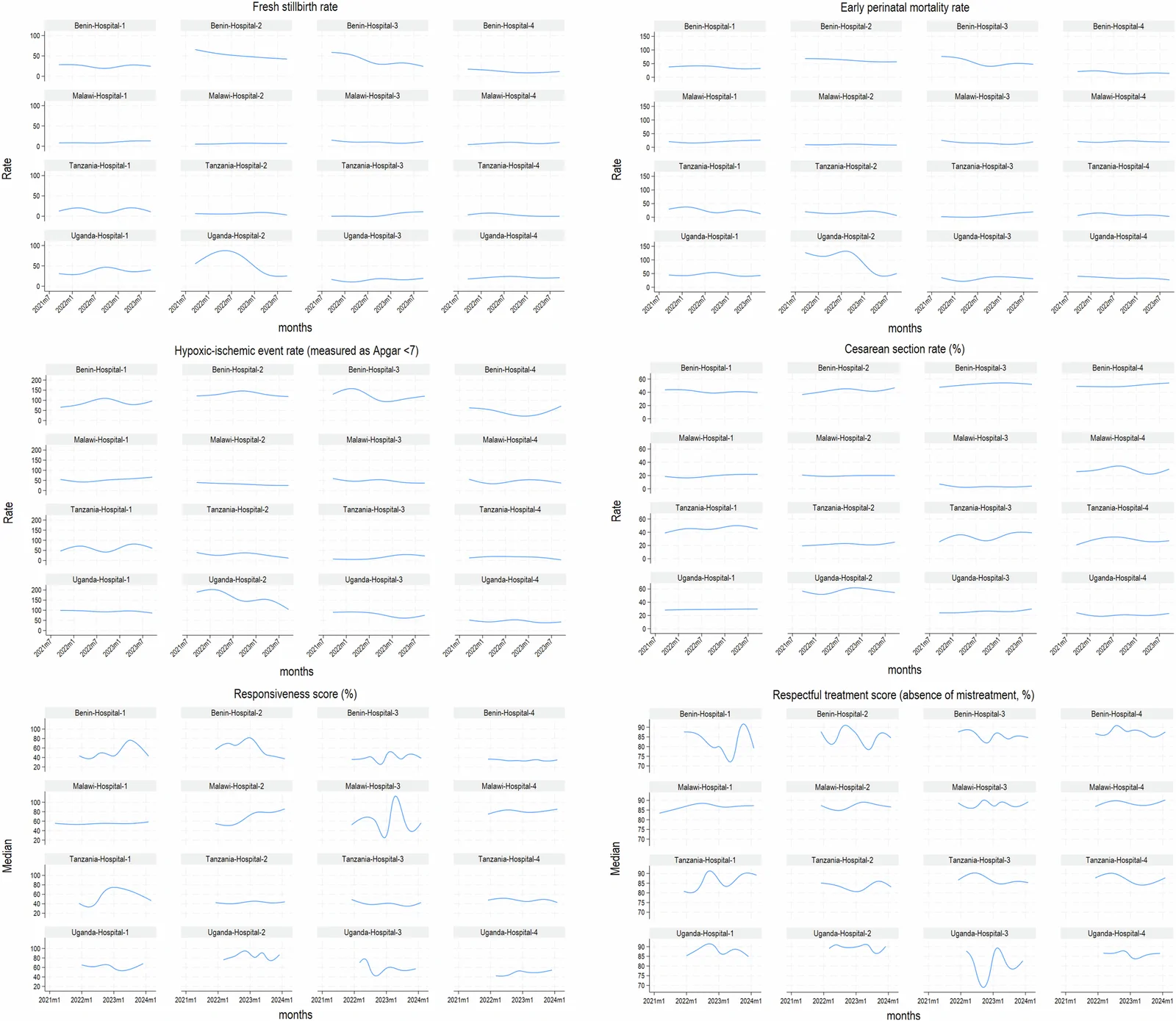
Comparison of outcomes, changes over time by hospital. In this analysis we provide a per-protocol analysis using an alternative definition of the intervention period to ensure full implementation fidelity.

Temporal trends over time across all facilities were examined for primary and select secondary outcomes (Extended Data Fig. 4). As missing data for the primary outcomes were minimal (<1%), complete case analysis was done. Statistical analyses were performed using Stata Standard Edition 18.

### Sensitivity analyses

In response to peer review, three pre-specified sensitivity analyses were additionally performed (HKSJ pooling, per-protocol re-analysis and country-stratified random-effects pool); full methodological detail and results are provided in Supplementary Methods and Supplementary Tables 5–7.

#### Hartung–Knapp–Sidik–Jonkman pooling

Because the number of clusters is small (*k* = 15–16), we re-pooled the hospital-specific estimates using the HKSJ method, which combines a restricted-maximum-likelihood estimate of between-hospital variance with a *t*-distribution on *k* − 1 degrees of freedom. Point estimates were essentially unchanged from the DerSimonian–Laird pool used in the main analysis, and CIs were slightly wider (Supplementary Table 6).

#### Per-protocol analysis considering quality concerns

Three hospitals (Tanzania Hospitals 3 and 4 and Malawi Hospital 3) were prospectively identified as unable to deliver the intervention package, following country-team review meetings in December 2024 and before the analytical code was locked. Three criteria, agreed in advance, were applied: (i) documented absence of core intervention components (for example, Malawi Hospital 3 had no functional fetal monitoring equipment); (ii) implementation barriers recorded in site-level process-evaluation notes, such as low staff engagement and concerns about data fidelity at Tanzania Hospital 3 following an external audit, and low case volume with incomplete outcome data at Tanzania Hospital 4; and (iii) hospital-period cells in which the full package was not delivered, captured by the trial’s ‘perprotocol’ indicator. The hospitals meeting these criteria are listed in Supplementary Table 4. The pool was restricted to hospital-periods with ‘perprotocol = 1’. Point estimates attenuated for most outcomes, but their direction was unchanged; between-hospital heterogeneity (*I*^2^) fell substantially (Supplementary Table 7).

#### Country-stratified pooling

The 16 hospital-specific estimates were pooled within each country, and a between-country *Q*-test with 3 degrees of freedom was used to test for heterogeneity of treatment effect across Benin, Malawi, Tanzania and Uganda. For the primary outcome of early perinatal mortality, there was no evidence of between-country heterogeneity (*Q*ᵇ(3) = 1.61, *P* = 0.66). For fresh stillbirth, cesarean section, severe maternal morbidity, fetal distress and breastfeeding within 1 h, between-country heterogeneity was significant (*P* < 0.05) (Supplementary Table 7).

### Protocol deviations

We were not able to implement lactate testing as a measure independent of clinical judgment to indicate hypoxic–ischemic events for operational and financial reasons. However, we included lactate testing using the Nova lactate point-of-care meter for 437 cases of Apgar <7 in 2 hospitals in Uganda. A total of 420 of 437 (96%) neonates born with an Apgar <7 had a lactate level above 5.5 mmol l^−1^, confirming earlier work indicating reasonable sensitivity and specificity of the Apgar score in this setting^52,74^. We were unable to construct the process indicator decision-to-birth time for cesarean section because the team did not document this consistently.

### Reporting summary

Further information on research design is available in the Nature Portfolio Reporting Summary linked to this article.

## Online content

Any methods, additional references, Nature Portfolio reporting summaries, source data, extended data, supplementary information, acknowledgements, peer review information; details of author contributions and competing interests; and statements of data and code availability are available at https://doi.org/10.1038/s41591-026-04599-w.

## Supplementary information

Supplementary Information Table 1: Implementation context. Table 2: Baseline data (maternal and child/birth). Table 3: Maternal outcome and process indicators data from e-registry and exit interviews. Table 4: Hospitals excluded in sensitivity analysis. Table 5: Comparison of DerSimonian–Laird and Hartung–Knapp–Sidik–Jonkman random-effects pooling methods. Table 6: Per-protocol sensitivity analysis restricted to hospital-periods with documented full intervention uptake. Table 7: Country-stratified random-effects pool with test for between-country heterogeneity. Table 8: Definitions of ALERT trial outcomes. Statistical Analysis Plan.

Reporting Summary

Peer Review File

## Data Availability

Data from the perinatal e-registry will be made available from 31 December 2027, which is 3 years following the finalization of the trial, in accordance with the ALERT data management plan and publication policy. This restriction was instituted to allow country researchers from Benin, Malawi, Tanzania and Uganda, particularly early-career researchers and PhD students, time to conduct analyses and publish findings. Before this date, data can be made available after consideration by the ALERT steering committee, which is led by the corresponding author, C. H. The ALERT data dictionary and supporting documents are available at https://cordis.europa.eu/project/id/847824/results. Researchers from any country with an affiliation to a recognized university or research institution are eligible to apply for access to the ALERT dataset, and applicants should submit a detailed project proposal (no more than five pages) to the corresponding author, outlining the project aims, methodology, requested variables, planned statistical analyses, evidence of ethics approval and benefit to local countries. The criteria used to evaluate the requests are (i) scientific merit, (ii) feasibility, (iii) benefit for local countries and (iv) ethical considerations. The ALERT steering committee will respond to all data requests within 1 month and, if approved, the data will be released shortly after approval. Access to data may be restricted if members of the ALERT team are planning or conducting similar analyses.

## Code availability

The code is available via Zenodo at https://doi.org/10.5281/zenodo.20671445 (ref. ^75^).
