# Supplementary material for: A multi-faceted hospital-based intervention for intrapartum care in sub-Saharan Africa: a stepped-wedge cluster-randomized trial
Source: Nat Med. 2026 Aug 26;32(10):3832–41. doi: 10.1038/s41591-026-04599-w (PMC13668680; doi:10.1038/s41591-026-04599-w)
Supplement: Supplementary file 1 [file 41591_2026_4599_MOESM1_ESM.pdf]

nature medicine

Supplementary information https://doi.org/10.1038/s41591-026-04599-w

A multi-faceted hospital-based intervention for intrapartum care in sub-Saharan Africa: a stepped-wedge cluster-randomized trial

In the format provided by the authors and unedited

Supplementary Information

Table of contents

Table S1. Implementation context ... 2

Table S2: Baseline data (maternal and child/birth) ... 3

Table S3: Maternal outcome and process indicators data from e-registry and exit interviews ... 4

Table S4: Hospitals excluded in sensitivity analysis ... 5

Table S5: Comparison of DerSimonian-Laird and Hartung-Knapp-Sidik-Jonkman random-effects pooling methods ... 6

Table S6: Per-protocol sensitivity analysis restricted to hospital-periods with documented full intervention uptake ... 7

Table S7: Country-stratified random-effects pool with test for between-country heterogeneity ... 8

Table S8: Definitions of ALERT trial outcomes ... 9

Statistical analysis plan SAP S9: 1st draft and final draft of the statistical plan ... 10

Table S1. Implementation context

Indicator	Benin	Malawi	Tanzania	Uganda
**National Indicators**				
Region in Africa	West	East	East	East
Real Gross Domestic Product per capita (US$) (2023) [1]	3,700	1,600	3,600	2,800
Population (million) (2023) [1]	14.2	21.3	65.6	47.7
**Neonatal Health Indicators**				
Neonatal Mortality Rate (per 1,000 live births) (2021) [2]	29	19	20	19
Stillbirth Rate (per 1,000 live births) (2021) [2]	20	16	18	15
**Maternal Health Indicators**				
Maternal Mortality Ratio (per 100,000 live births) (2020) [3]	523	381	238	284
Antenatal Care[2]				
≥1 Visits (%) (2022)[2]	78.1%	96.8% (2020)	89.7%	95% (2018)
≥4 Visits (%) (2022)[2]	52.6%	51% (2020)	65.1%	57% (2018)
Deliveries by Skilled Birth Attendant (%) (2022)[2]	80.8%	96.4% (2020)	85%	91%
Institutional Deliveries (%) (2022)[2]	90.6%	96.7% (2020)	81.2%	83.9% (2018)
Cesarean Section Deliveries (%) (2022)[2]	5.7%	7.7% (2020)	10.8%	6.2% (2016)
Official User Fees for Childbirth	Yes	No	No	No
Total Fertility (2022)[2]	4.5	3.6	4.6	4.3
Unmet Need for Family Planning (2022)[4]	16.9%	64.7% (2020)	31.1%	38%
**Health System Indicators**[5]				
Medical Doctors (per 10,000 population) (2022)	2.16	0.54	1.34	1.91
Nursing and Midwifery (per 10,000 population) (2023)	6.11	5.1 (2022)	6.93	22.65 (2022)
Current Health Expenditure per Capita (USD) (2022)	33.88	39.66	37.56	51.03
Current Health Expenditure / % of Gross Domestic Product (2022)	2.7%	6.5%	3.1%	4.3%
Out of Pocket Expenditure / % of Current Health Expenditure (2020)	42.5%	12.1%	28.7%	34.1%
**Hospital characteristics**				
Public hospital / private-not-for-non-profit hospitals	3 / 1	3 / 1	3 / 1	3 / 1
Hospital with dedicated Cesarean section theatre	4	1	4	0
Providers of Cesarean section services ^	MD / specialists	Clinician	Clinician* / MD	MD
Midwifery providers	Midwives	Nurse/Midwives	Nurse/Midwives	Midwives

1. CIA. The Global Factbook. 2024. https://www.cia.gov/the-world-factbook/africa/ (accessed 22 Dec 2024).

2. UN Inter-agency Group for Child Mortality Estimation. Levels and Trends in Child Mortality. 2024. https://childmortality.org/ (accessed 26 Nov 2024).

3. World Health Organization. Trends in maternal mortality 2000 to 2020: estimates by WHO, UNICEF, UNFPA, World Bank Group and UNDESA/Population Division. Geneva, 2023.

4. United Nations Population Fund. Population Data Portal [Internet]. 2022. Available from: https://www.unfpa.org/data

5. World Health Organization. Global Health Observatory: Data Repository [Internet]. WHO. World Health Organization; Available from: https://apps.who.int/gho/data/node.main

^MD Medical Doctor, Clinic related to non-physician clinicians

Table S2: Baseline data (maternal and child/birth)

MATERNAL CHARACTERISTICS				
	**Benin**	**Malawi**	**Tanzania**	**Uganda**
	**N=27867**	**N=47176**	**N=21579**	**N=38008**
Maternal Age *(122 missing)*				
<20	2852 (10.2%)	15149 (32.1%)	4319 (20.0%)	6792 (17.8%)
20-24	7243 (26.0%)	14499 (30.7%)	5965 (27.6%)	12849 (33.8%)
25-29	7672 (27.5%)	8084 (17.1%)	4165 (19.3%)	9336 (24.6%)
30-34	5914 (21.2%)	5141 (10.9%)	3677 (17.0%)	5573 (14.7%)
35+	4171 (15.0%)	4205 (8.9%)	3453 (16.0%)	3449 (9.0%)
Women referred into facility	**14770 (53.0%)**	**10595 (22.5%)**	**1259 (5.8%)**	**6836 (18.1%)**
Stage of labor when referred in *(74 missing)*	***n=14770***	***n=10595***	***n=1259***	***n=6836***
Pregnancy-not in labor	*2015 (13.6%)*	*826 (7.8%)*	*235 (18.7%)*	*315 (4.6%)*
Labor	*12725 (86.2%)*	*9755 (92.1%)*	*1021 (81.1%)*	*6494 (95.0%)*
Referring facility *(31 missing)*	***n=14770***	***n=10595***	***n=1259***	***n=6836***
Home / community	*97 (0.7%)*	*6290 (59.4%)*	*207 (16.4%)*	*1473 (21.6%)*
Lower-level facility	*13261 (89.8%)*	*4276 (40.4%)*	*966 (76.7%)*	*4635 (67.8%)*
Other hospital	*1399 (9.5%)*	*25 (0.2%)*	*79 (6.3%)*	*721 (10.6%)*
Gravidity, median (IQR) *(95 missing)*	**3.0 (1.0, 4.0)**	**2.0 (1.0, 3.0)**	**2.0 (1.0, 3.0)**	**2.0 (1.0, 4.0)**
Parity – categorized				
Nullipara	8922 (32.0%)	22202 (47.1%)	8177 (37.9%)	13863 (36.5%)
1-2	10160 (36.5%)	16786 (35.6%)	9280 (43.0%)	14568 (38.3%)
3-4	6194 (22.2%)	6470 (13.7%)	3481 (16.1%)	6696 (17.6%)
5+	2591 (9.3%)	1718 (3.6%)	641 (3.0%)	2881 (7.6%)
Previous Cesarean section	**6526 (32.7%)**	**3207 (12.3%)**	**2266 (16.3%)**	**4625 (18.3%)**
Number of ANC visits, current pregnancy *(1107 missing)*				
<4	7581 (27.2%)	22863 (48.5%)	1860 (8.6%)	16297 (42.9%)
4+	20051 (72.0%)	23968 (50.8%)	19717 (91.4%)	21186 (55.7%)
Multiple pregnancy	**2549 (9.2%)**	**1494 (3.2%)**	**371 (1.7%)**	**995 (2.6%)**
Maternal comorbidities				
Hypertensive disorders	4763 (17.1%)	522 (1.1%)	875 (4.1%)	1642 (4.3%)
Preterm labor	3223 (11.6%)	1678 (3.6%)	585 (2.7%)	1659 (4.4%)
Augmentation of labor	931 (4.5%)	164 (0.4%)	544 (2.9%)	401 (1.2%)
CHILD CHARACTERISTICS				
	**Benin**	**Malawi**	**Tanzania**	**Uganda**
	**N=30160**	**N=47998**	**N=21999**	**N=39143**
Sex at birth				
Female	14374 (47.7%)	23247 (48.4%)	10894 (49.5%)	19628 (50.1%)
Male	15768 (52.3%)	24735 (51.5%)	11098 (50.5%)	19469 (49.7%)

**Missing data distribution: Maternal age 15 (0.1%) in Benin; 98 (0.2%) in Malawi; 0 (0.0%) in Tanzania and 9 (<1%) in Uganda. Also by study arm 62 (0.1%) missing in the comparison arm compared to 60 (0.1%) in the intervention arm; Sex at birth 87 (0.1%), 18 (0.1%); 16 (0.0%) and 46 (0.1%)*

Table S3: Maternal outcome and process indicators data from e-registry and exit interviews

	Benin	Malawi	Tanzania	Uganda	Comparison Arm	Intervention Arm
**Primary and secondary outcomes**						
Primary outcomes *(from e-registry; child attributes)*						
Number of births recorded	**N=30160**	**N=47998**	**N=21999**	**N=39143**	**N=62244**	**N=77056**
Fresh stillbirths	1192 (4.0%)	458 (1.0%)	149 (0.7%)	1069 (2.7%)	1223 (2.0%)	1645 (2.1%)
Early perinatal mortality	1517 (5.0%)	855 (1.8%)	323 (1.5%)	1582 (4.0%)	1905 (3.1%)	2372 (3.1%)
Secondary outcomes (from e-registry; child attributes)						
Hypoxic-ischemic event measured as low Apgar score (<7)	3379 (11.2%)	2157 (4.5%)	616 (2.8%)	3219 (8.2%)	4063 (6.5%)	5308 (6.9%)
Neonatal seizures	108 (0.4%)	65 (0.1%)	118 (0.5%)	86 (0.2%)	142 (0.2%)	235 (0.3%)
Cesarean section	13552 (44.9%)	8499 (17.7%)	6502 (29.6%)	11150 (28.5%)	17075 (27.4%)	22628 (29.4%)
Secondary outcomes (from e-registry; maternal attributes)						
Number of women recorded	**N=27867**	**N=47176**	**N=21579**	**N=38008**	**N=60403**	**N=74227**
Severe maternal morbidity	1486 (5.3%)	591 (1.3%)	428 (2.0%)	768 (2.0%)	1544 (2.6%)	1729 (2.3%)
Maternal Attributes: Secondary outcomes from exit interviews						
Number of women recorded	**N=1003**	**N=1004**	**N=996**	**N=1004**	**N=2036**	**N=1971**
Responsiveness score, median (IQR)	39.2 (33.8, 51.5)	61.3 (52.0, 81.6)	45.0 (37.7, 51.9)	62.7 (50.5, 76.6)	49.4 (38.6, 63.9)	54.5 (42.3, 71.6)
Respectful treatment score, median (IQR)	86.1 (83.7, 90.1)	90.9 (86.9, 91.7)	87.7 (84.5, 90.1)	86.9 (84.5, 90.9)	88.5 (85.3, 90.9)	87.7 (84.5, 90.9)
Process indicators						
Process indicators (from e-registry, maternal attributes)						
Number of women recorded	**N=27867**	**N=47176**	**N=21579**	**N=38008**	**N=60403**	**N=74227**
Fetal heart check at admission done	24107 (86.5%)	44996 (95.4%)	19911 (92.3%)	35126 (92.4%)	53635 (88.8%)	70505 (95.0%)
Fetal monitoring done and documented in 1st stage of labor	20313 (72.9%)	32486 (68.9%)	20259 (93.9%)	22377 (58.9%)	40488 (67.0%)	54947 (74.0%)
Resuscitation for babies with Apgar < 7	1216 (36.0%)	1386 (64.3%)	223 (36.2%)	1089 (33.8%)	1565 (38.5%)	2349 (44.3%)
Maternal Attributes: Process indicators from exit interviews						
	**N=1003**	**N=1004**	**N=996**	**N=1004**	**N=2036**	**N=1971**
*Companion of choice first or second stage**	90 (9.0%)	419 (41.7%)	43 (4.3%)	955 (95.2%)	743 (36.5%)	764 (38.8%)
*Companion of choice in 2nd stage**	48 (4.8%)	337 (33.6%)	20 (2.0%)	688 (68.5%)	527 (25.9%)	566 (28.7%)
*Early ambulation /Woman encouraged to walk during labor**	142 (14.2%)	682 (67.9%)	362 (36.4)	719 (71.6%)	958 (47.1%)	947 (48.1%)
*Initiated breastfeeding within 1 hour**	41 (4.1%)	813 (81.0%)	529 (53.1%)	701 (69.8%)	1090 (53.5%)	994 (50.4%)

**Missing data distribution: Companion of choice first or second stage in Uganda 1(0.1%) and comparison arm 1 (0.1%); Companion of choice in 2nd stage in Benin 185 (18.5%), Malawi 21 (2.1%), Tanzania 137 (13.8%) and Uganda 100 (10.0%) and overall in comparison arm 168 (8.3%) and intervention arm 275 (13.9%); Early ambulation /Woman encouraged to walk during labor in Benin 122 (12.2%), Malawi 121 (12.1%), Tanzania 459 (46.1%), and Uganda 102 (10.2%) and overall in comparison arm 382 (18.8%) and intervention arm 422 (21.4%); Initiated breastfeeding within 1 hour in Benin 46 (4.6%), Malawi 41 (4.1%), Tanzania 39 (3.9%), and Uganda 45 (4.5%) and overall in comparison arm 95 (4.7%) and intervention arm 76 (3.9%)*

Table S4: Hospitals excluded in sensitivity analysis

Country	Hospitals excluded	Primary Reason for Exclusion	Implementation Barriers	Impact on Outcomes
**Benin**	*(None excluded)*	N/A	• Variable engagement across hospitals but good data quality<br>• Parallel improvements at Hospital 3 due to external investments (infrastructure and equipment)	• Variability reflected true contextual differences; no exclusion applied
**Malawi**	**Hospital 3**	Lack of fetal monitoring equipment	• No functional fetal monitoring equipment<br>• Community hospital with severe resource constraints<br>• Governance issues and weak supervision<br>• Referred complicated cases elsewhere	• "Complete outlier" for fetal monitoring indicators<br>• Process indicators "not applicable"<br>• Core intervention components unimplementable
**Tanzania**	**Hospital 3**	Lack of staff engagement and weak data quality	• Staff poorly accountable and not committed to change<br>• Fetal monitoring severely limited<br>• Least engaged facility in the intervention<br>• Frequent power shortages	• Fresh stillbirth rate increased ~5-fold<br>• Very small numbers and wide confidence intervals<br>• Removal increased overall intervention effect
	**Hospital 4**	Very low case volume and missing outcome data	• Small hospital with few births and neonatal events<br>• Weak documentation and incomplete reporting<br>• Minimal staff participation in QI activities	• Contributed negligible cases to outcome estimates<br>• Removal improved model stability for neonatal outcomes
**Uganda**	*(None excluded)*	N/A	1. All differences attributed to contextual and staffing variation<br>2. Hospital 1 constrained by space for companions.<br>3. Hospital 4 had short intervention duration	4. No hospitals identified as statistical or qualitative outliers, therefore, all retained in analysis

Table S5: Comparison of DerSimonian-Laird and Hartung-Knapp-Sidik-Jonkman random-effects pooling methods

Indicator	k (hospitals)	DerSimonian-Laird OR (95% CI)	p-value	Hartung-Knapp-Sidik-Jonkman OR (95% CI)	p-value	I² (%)
Primary outcomes						
Fresh stillbirth rate	15	0.88 (0.69-1.13)	0.324	0.88 (0.66-1.18)	0.367	69.6
Early perinatal mortality rate	16	0.78 (0.66-0.94)	0.008	0.79 (0.63-0.98)	0.034	57.8
Secondary outcomes						
24-hour neonatal mortality rate	16	0.68 (0.46-1.00)	0.051	0.68 (0.43-1.08)	0.097	75.1
Hypoxic-ischemic event (Apgar <7)	16	0.81 (0.69-0.95)	0.008	0.80 (0.63-1.02)	0.070	77.8
Neonatal seizures	14	0.79 (0.55-1.15)	0.223	0.79 (0.56-1.11)	0.164	0.0
Cesarean section	16	1.14 (1.01-1.27)	0.027	1.14 (0.99-1.30)	0.070	87.5
Severe maternal morbidity	16	0.72 (0.53-0.97)	0.032	0.71 (0.48-1.05)	0.080	82.8
Fetal distress (diagnosed)	16	0.54 (0.38-0.76)	<0.001	0.54 (0.35-0.82)	0.007	87.7
Breastfeeding within 1 hour	16	0.79 (0.58-1.06)	0.113	0.79 (0.53-1.17)	0.215	97.8

DerSimonian-Laird (DL) is the estimator used in the main analysis. Hartung-Knapp-Sidik-Jonkman (HKSJ) combines a restricted-maximum-likelihood estimate of between-hospital variance with a t-distribution on k-1 degrees of freedom, and is recommended when the number of clusters is small. Both pools use the same hospital-specific log-odds ratios, so point estimates agree to ~0.01 but HKSJ confidence intervals are systematically wider. k = number of hospitals contributing an estimable log-odds ratio; I² is reported from the DL pool.

Table S6: Per-protocol sensitivity analysis restricted to hospital-periods with documented full intervention uptake

Indicator	Main analysis OR (95% CI)	Per-protocol OR (95% CI)	p-value (per-protocol)	k main / k PP	I² (%) main / PP
Primary outcomes					
Fresh stillbirth rate	0.88 (0.69-1.13)	0.89 (0.66-1.18)	0.417	15 / 15	69.6 / 40.3
Early perinatal mortality rate	0.78 (0.66-0.94)	0.96 (0.76-1.21)	0.746	16 / 16	57.8 / 34.5
Secondary outcomes					
24-hour neonatal mortality rate	0.68 (0.46-1.00)	0.87 (0.65-1.15)	0.319	16 / 13	75.1 / 0.0
Hypoxic-ischemic event (Apgar <7)	0.81 (0.69-0.95)	1.01 (0.84-1.21)	0.948	16 / 15	77.8 / 54.9
Neonatal seizures	0.79 (0.55-1.15)	0.72 (0.40-1.31)	0.284	14 / 8	0.0 / 21.3
Cesarean section	1.14 (1.01-1.27)	0.98 (0.87-1.09)	0.675	16 / 16	87.5 / 64.5
Severe maternal morbidity	0.72 (0.53-0.97)	1.03 (0.74-1.44)	0.858	16 / 16	82.8 / 62.1
Fetal distress (diagnosed)	0.54 (0.38-0.76)	0.83 (0.63-1.08)	0.170	16 / 16	87.7 / 61.2
Breastfeeding within 1 hour	0.79 (0.58-1.06)	1.10 (0.86-1.41)	0.428	16 / 15	97.8 / 90.3

The per-protocol subset is defined by the binary ‘perprotocol’ indicator in the trial dataset, which flags hospital-period cells where the intervention was delivered in full. Hospital-specific effects were re-estimated from the restricted dataset using the same mixed-effects logistic regression and pooled with the DerSimonian-Laird random-effects estimator. Some outcomes contribute fewer than 16 hospitals because a site had no events during per-protocol periods (seizures, 24-h neonatal mortality).

Table S7: Country-stratified random-effects pool with test for between-country heterogeneity

Indicator	Benin OR (95% CI)	Malawi OR (95% CI)	Tanzania OR (95% CI)	Uganda OR (95% CI)	Overall OR (95% CI)	Between-country test $Q^b(3)$, p
Primary outcomes						
Fresh stillbirth rate	0.63 (0.50-0.79)	1.11 (0.82-1.50)	1.03 (0.25-4.22)	0.96 (0.61-1.52)	0.88 (0.69-1.13)	Q^b = 9.43, p = 0.024
Early perinatal mortality rate	0.69 (0.58-0.82)	0.85 (0.60-1.20)	0.83 (0.25-2.83)	0.82 (0.61-1.09)	0.78 (0.66-0.94)	Q^b = 1.61, p = 0.657
Secondary outcomes						
24-hour neonatal mortality rate	1.21 (0.46-3.18)	0.64 (0.40-1.02)	1.11 (0.25-4.98)	0.42 (0.27-0.66)	0.68 (0.46-1.00)	Q^b = 5.04, p = 0.169
Hypoxic-ischemic event (Apgar <7)	0.94 (0.78-1.14)	0.78 (0.54-1.12)	0.73 (0.27-1.94)	0.76 (0.62-0.94)	0.81 (0.69-0.95)	Q^b = 2.54, p = 0.468
Neonatal seizures	1.08 (0.57-2.04)	1.05 (0.35-3.13)	0.59 (0.28-1.25)	0.53 (0.24-1.17)	0.79 (0.55-1.15)	Q^b = 2.72, p = 0.436
Cesarean section	1.13 (0.86-1.49)	0.95 (0.78-1.16)	1.48 (1.29-1.69)	1.07 (0.87-1.32)	1.14 (1.01-1.27)	Q^b = 15.87, p = 0.001
Severe maternal morbidity	1.17 (0.98-1.39)	0.51 (0.19-1.36)	0.78 (0.50-1.22)	0.49 (0.26-0.90)	0.72 (0.53-0.97)	Q^b = 11.00, p = 0.012
Fetal distress (diagnosed)	0.40 (0.15-1.10)	0.36 (0.17-0.78)	1.13 (0.70-1.84)	0.51 (0.30-0.86)	0.54 (0.38-0.76)	Q^b = 8.98, p = 0.030
Breastfeeding within 1 hour	0.40 (0.17-0.93)	1.64 (1.14-2.37)	0.56 (0.30-1.05)	1.01 (0.78-1.32)	0.79 (0.58-1.06)	Q^b = 14.69, p = 0.002

Country-stratified DerSimonian-Laird random-effects meta-analysis of the 16 hospital-specific adjusted odds ratios, with four hospitals per country (Benin, Malawi, Tanzania, Uganda). The overall estimate is the pooled effect across all contributing hospitals and is identical to the main-analysis pool. Q^b tests the null hypothesis of no between-country difference in the pooled treatment effect (3 degrees of freedom). Note that fresh stillbirth, Cesarean section, severe maternal morbidity, fetal distress and breastfeeding show statistically detectable between-country variation ($p < 0.05$), which motivates the primary two-stage analytical approach.

Method section

Table S8: Definitions of ALERT trial outcomes

Indicators	***Definitions***	***Methods***
Primary outcome		
Fresh stillbirth rate	Number of fresh stillbirths of at least 1000g per 1000 live- and stillbirth	e-registry
Early perinatal mortality rate	Number of fresh stillbirths and 24-hour neonatal deaths per 1000 live- and stillbirths (composite indicator)	e-registry
Secondary outcomes		
Hypoxic-ischemic event rate	No of neonates with Apgar < 7 at 5 min per 1000 live- and stillbirths	e-registry
Neonatal seizures	No of neonates diagnosed with seizures per 1000 live- and stillbirths	e-registry
Cesarean section (%)	No of Cesarean section per 100 live- and stillbirths	e-registry
Severe maternal morbidity (%)	No of women with morbidities # per 100 live- and stillbirths (women with hysterectomy, laparotomy, blood transfusion, admission to intensive care unit or referral to higher level facility)	e-registry
Responsiveness score (%)	Validated score (%)	Exit interviews
Respectful treatment score (%)	Validated score (%)	Exit interviews
Process indicators		
Admission standards and procedures		e-registry
Fetal heart check on admission (%)	Percentage of babies with fetal check at admission done	
Intrapartum monitoring		
Fetal monitoring in 1st stage of labor	Percentage of babies with fetal monitoring done and documented in 1st stage	e-registry
Emergency preparedness		
Resuscitation for babies with Apgar < 7	Percentage of babies with Apgar < 7 who were resuscitated	e-registry
Responsiveness-focused		
Companion present during labor or birth	Percentage of women who reported having a companion during labor or birth	Exit interviews
Early newborn care		
Breastfeeding within 1 hour	Breastfeeding within 1 hour after birth	Exit interviews

Statistical analysis plan SAP9: 1st draft and final draft of the statistical plan

STATISTICAL ANALYSIS PLAN

ALERT

ACTION LEVERAGING EVIDENCE TO REDUCE PERINATAL MORTALITY AND MORBIDITY IN SUB-SAHARAN AFRICA (ALERT) TRIAL

FINAL VERSION APPROVED BY:

Name	Signature	Date
Professor Claudia Hansen		**30 March 2023**
Professor Nicola Orsini		

DRAFT DEVELOPED BY:

Name	Signature	Date
Dr. Joseph Akuze		**23 March 2023**
Dr. Kristi Sidney Annerstedt		**23 March 2023**

**VISION HISTORY**<br><br>**CHRONOLOGY:**		
**VERSION NUMBER**	**EFFECTIVE DATE**	**REVIEWED BY (NAME)**
V1	2023/03/23	Joseph Akuze

Table of Contents

1. STUDY BACKGROUND 1

2. STUDY OBJECTIVES 1

2.1 Specific Objective 1

2.2 Assessment of Objectives 1

3. STUDY DESIGN 4

3.1 Overview 4

3.2 Sample Size 4

3.3 Randomization and Blinding 4

Figure 3a: Randomisation syntax file 5

3.4 Study Assessments 6

4. STUDY POPULATIONS 6

4.1 Subject Disposition 6

4.2 Definition of Populations for Analysis 7

4.3 Definition of Sub-Group Population in Different Analyses 7

4.4 Efficacy Evaluable in the ALERT trial 8

4.5 Intent-to-treat (ITT) 8

4.6. Study Sites and Populations 8

4.7 Major Protocol Deviations 10

5. STATISTICAL ANALYSIS 10

5.1. Outcome measures 10

5.2. Analytical approaches 10

Descriptive statistics 10

Multivariable analysis 14

5.3 Pooling of Sites 16

5.4 Interim Analyses 16

5.5 Time-Points For Analysis 17

5.6 Methods for Handling Missing Data 17

1. STUDY BACKGROUND

The Action Leveraging Evidence to reduce perinatal Mortality and morbidity in sub-Saharan Africa (ALERT) trial was set to develop and evaluate a comprehensive and multilevel intervention covering intrapartum care and midwifery. The ALERT trial and interventions targeted hospital maternity units and included i) end-user participation of women, families, and midwifery providers to co-design the intervention; ii) in-service midwifery competency-based training; iii) empowerment and leadership mentoring of maternity unit leaders, and iv) quality improvement in the maternity ward, supported by district-based bi-annual coordination and accountability meetings (Fig. 1).

Figure 1: ALERT Conceptual Framework

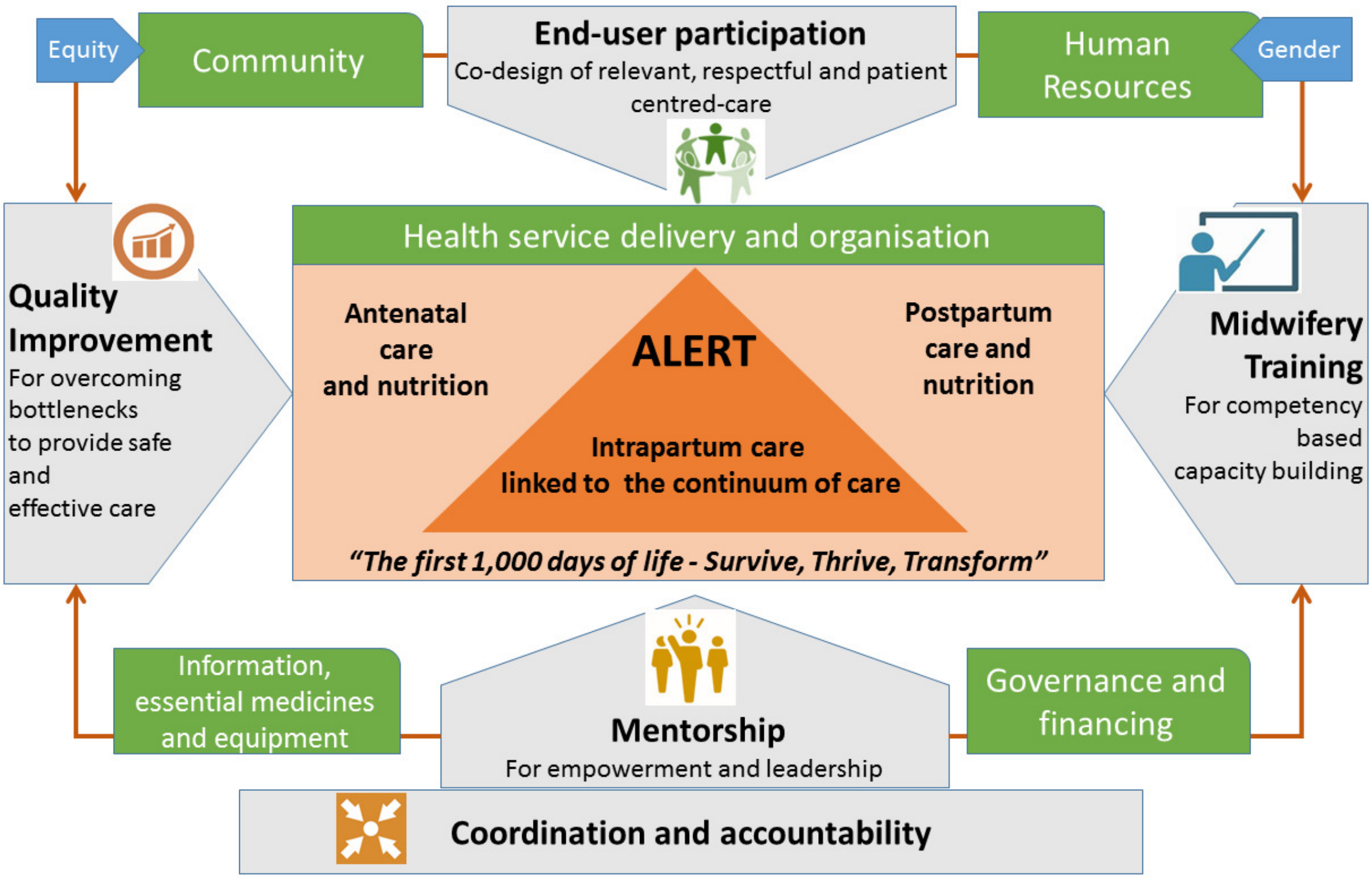

2. STUDY OBJECTIVES

2.1 Specific Objective

The ALERT trial had three specific study objectives

1. To assess the ALERT intervention's impact in hospital maternity units on perinatal and maternal health outcomes, including women's experience of care.
2. To evaluate the process of implementation of the intervention to understand what works for whom and under what situation.
3. To conduct a cost-effectiveness analysis of the ALERT intervention.

2.2 Assessment of Objectives

A summary of our approach to answering the objectives is given in Table 1. In this Analysis Plan, we give details for only objective one (details about other study objectives are given elsewhere).

Table 1:Methods and data sources by study objective

**Objective**	**Methods**	**Data Source**	**Notes and links**
Obj 1. To assess the ALERT intervention's impact in hospital maternity units on perinatal and maternal health outcomes, including women's experience of care.	Trial profile<br><br>Assessment of recruitment rate, loss to follow-up and records completeness/incompleteness<br><br>- Number of records entered the REDCap database in comparison to expected number of records<br>- Number of incomplete records<br><br>Descriptive statistics (counts, proportions, mean, median, SD, max, min)<br><br>– Study outcomes split by intervention arm<br>– Additionally, we will summarise the primary outcome split by both site and by intervention condition.<br><br>Regression models fitted for the primary outcome<br><br>– Generalised Estimation Equations models will be used to adjust for the effect of clustering and repeated measures over time.<br>– Intra cluster correlation (ICC) coefficients will be re-evaluated using the study data.<br>– Unidirectional crossover as well as any potential secular trends may be incorporated with a time effect<br>– Sensitivity analysis	Trial data<br><br>Exit interviews	Our primary outcome is in-facility early perinatal mortality defined as in-facility (fresh) stillbirth and 24-hour neonatal mortality

**Objective**	**Methods**	**Data Source**	**Notes and links**
	Women's experiences of care<br>- Descriptive statistics for each hospital, country (means and proportions)<br>- Distribution of the of optimal care and number of interventions received.<br>- Regression models to predict factors that determine optimal women's care<br>- Spatial analysis? – Health seeking behaviours vis-a-viz care seeking		
Obj 2. To evaluate the process of implementation of the intervention to understand what works for whom and under what situation.	Trial profile<br><br>Descriptive statistics (counts, proportions, mean, median, SD, max, min)	WP7 – [WP7 should have a protocol]	
Obj 3. To conduct a cost-effectiveness analysis of the ALERT intervention.	Cost effectiveness analysis<br><br>- Provider's perspectives<br>- Effects per outcomes<br>- Incremental cost-effectiveness ratio (ICER)<br>- Sensitivity analysis	Costing data	

3. STUDY DESIGN

3.1 Overview

A stepped-wedge cluster-randomized design with a nested process evaluation based on realist evaluation was employed in the ALERT trial. This design was selected as the interventions were administered at the hospital level, and a stepped-wedge design was chosen to mirror scale-up for policy buy-in and for statistical efficiency as larger cluster-level differences are anticipated.

3.2 Sample Size

The ALERT trial was carried out in 16 hospitals, with four hospitals selected in each of the four countries: Benin, Malawi, Tanzania, and Uganda. The selection criteria for the hospitals required them to have a minimum of 2500 births per year, which provided sufficient statistical power (75%-80%) to detect a 25% reduction in the in-facility early perinatal mortality rates with a 95% confidence interval. This also allowed for the assessment of other secondary outcomes such as maternal morbidity, hypoxic-ischemic insults, neonatal seizures, and more (see Figure 2).

Figure 2: Sample size and Power calculations for ALERT trial outcomes

	**Baseline estimates**	**expected change**	**expected value**	**ICC**	**Power**
**Perinatal e-registry (Cluster size 1500 births)**					
Stillbirth	1.0%	30%	0.7%	0.0024	83%
Stillbirth	1.0%	30%	0.7%	0.024	82%
Perinatal mortality	2.0%	25%	1.5%	0.0024	93%
Perinatal mortality	2.0%	25%	1.5%	0.024	92%
Perinatal mortality	1.4%	25%	1.1%	0.0024	70%
Hypoxic-ischaemic insults	4.0%	30%	2.8%	0.0024	100%
Hypoxic-ischaemic insults (sub-group)	4.0%	30%	2.8%	0.0024	79%
Neonatal seizures	0.5%	50%	0.3%	0.0024	78%
Ceasarean section	15.0%	20%	18.0%	0.01	100%
Maternal morbidity	3.0%	30%	2.1%	0.5	99%
**Interviews post-partum (Cluster size 50 interviews)**					
Responsiveness	70.0%	15%	80.0%	0.1	97%
Mistreatment	4.0%	75%	1.0%	0.1	77%
Breastfeeding	60.0%	20%	71.0%	0.1	98%

3.3 Randomization and Blinding

The ALERT trial employed randomization, which was carried out by an independent statistician after obtaining consent from the participating hospitals. Stratification by country was used to enable random selection of four hospitals in each country (refer to randomization syntax in Figure 3a). Participants (hospitals) will not be blinded to the intervention, as with all training and quality improvement interventions. However, the women and their families receiving care at these hospitals will not be aware of the exact implementation stage of ALERT. The final sequence of ALERT intervention and data collection is illustrated in Figure 3b.

Figure 3a: Randomisation syntax file

```
/*

Objective: This do-file is showing how to create a file with the randomized sequence
Date: 20200617
Author: Orsini N
*/

* cd "/Users/nicors/OneDrive - KI.SE/Mac/Research/claudia/"

/*
* Import into Stata all these data contained in the Excel file

local list = "Benin Malawi Tanzania Uganda"
foreach s of local list {
    import excel "Hospitals for randomization .xlsx", sheet("`s'") clear
    gen state = "`s'"
    save `s', replace
}

* I append all these 4 stata dataset into a single dataset

use Benin, clear
append using Malawi Tanzania Uganda

rename B name
drop A
bysort state: gen code = _n
order state code
list , sepby(state) clean noobs

*/

* Another approach. Input the data manually. As shown below

clear
input    str10 state  code str50  name
       Benin      1          "Centre National Hospitalier Hubert Maga"
       Benin      2   "Hopital de Zone Come-Grand Popo Houeyogbe Bopa"
       Benin      3                      "Policlinique Bon Samaritan"
       Benin      4          "Centre Hospitalier et Dépt Zou-Collines"
      Malawi      1                                "Mchinji Hospital"
      Malawi      2                           "Mitundu Rural Hospital"
      Malawi      3                               "Ntcheu D Hospital"
      Malawi      4                            "St Gabriels Hospital"
    Tanzania      1                        "Newala district Hospital"
    Tanzania      2                              "Mkomaindo Hospital"
    Tanzania      3                                 "Ndanda Hospital"
    Tanzania      4                             "Nachingwea Hospital"
      Uganda      1                          "Bugiri General Hospital"
      Uganda      2                        "Iganga Referral Hospital"
      Uganda      3                         "Jinja Regional Hospital"
      Uganda      4                          "Kamuli Mission Hospitla"
end

* ssc install shuffle
* shuffle 1 2 3 4, num noisily

gen rand = .

* Set the seed to reproduce the randomization

set seed 20200617

local list = "Benin Malawi Tanzania Uganda"
qui foreach s of local list {
        local j = 1
        shuffle 1 2 3 4, num noisily
        local seq = r(list)
            foreach r of local seq {
                    replace rand = `r' if code == `j' & state == "`s'"
                    local j = `j' + 1
            }
}

list,  sepby(state)

export excel using "results_rand_200617", firstrow(variables) replace

exit
```

Figure 3b: Sequence of the ALERT Trial intervention

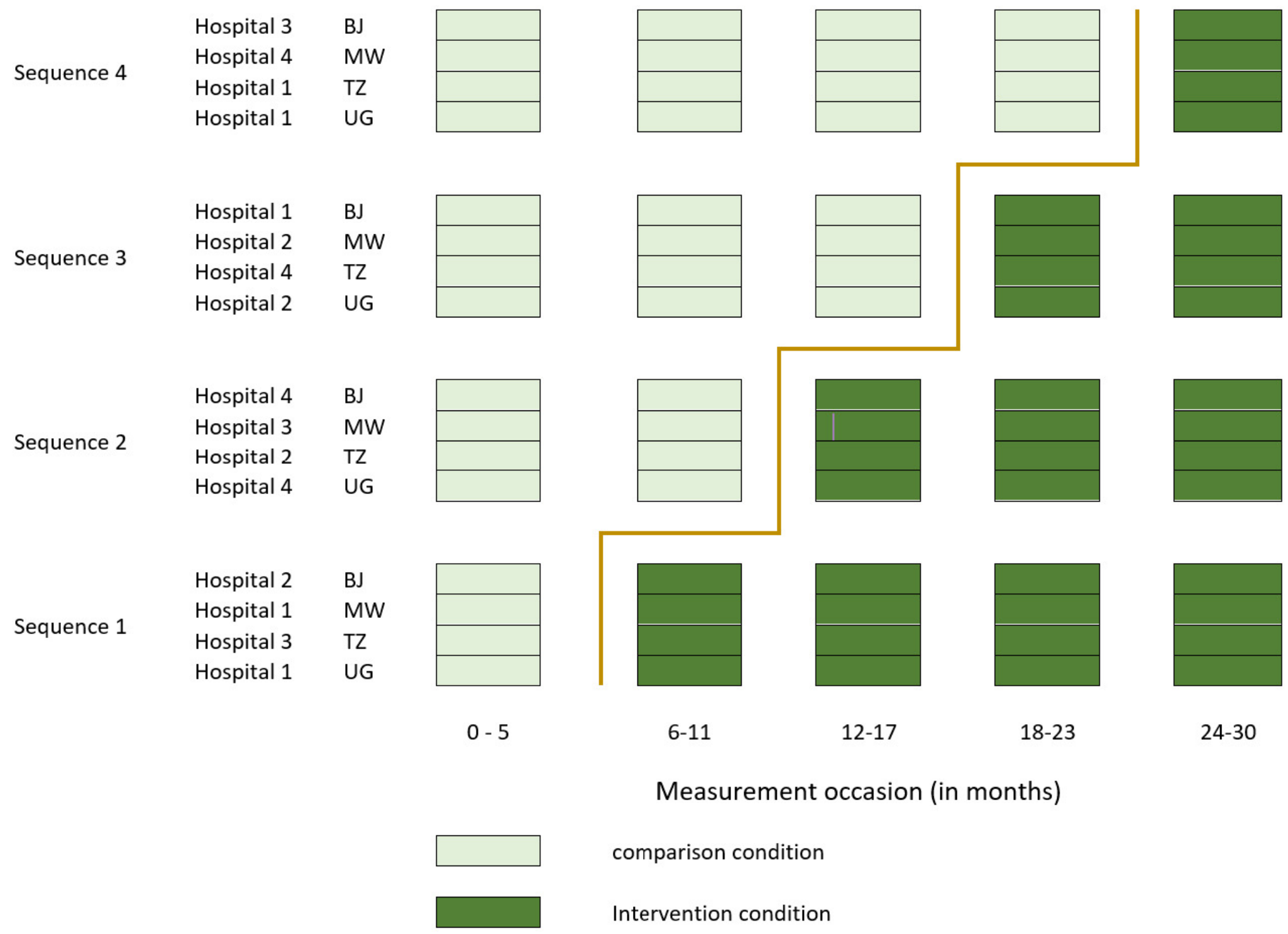

3.4 Study Assessments

To achieve objective one, we will conduct two assessments. Firstly, we will abstract trial data from hospital registries and enter it into a REDCap application. Secondly, we will conduct exit interviews with hospital clients to capture and evaluate their care-related experiences while in the hospitals.

4. STUDY POPULATIONS

4.1 Subject Disposition

Subject disposition in the ALERT trial refers to the movement of participants throughout the study, from screening to the completion of the intervention and follow-up. In the ALERT trial, subject disposition will be tracked for both hospital-level and patient-level participants.

At the hospital level, subject disposition will involve tracking the participation of the hospitals throughout the study. This includes monitoring their consent to participate, enrolment in the study, completion of the intervention, and follow-up data collection. Any dropout or non-compliance of the hospitals will also be recorded and reported.

At the patient level, subject disposition will involve tracking the participation of individual patients who are receiving care at the participating hospitals. This includes monitoring their enrolment in the study, completion of the intervention, and follow-up data collection. Any dropout or loss to follow-up of the patients will also be recorded and reported.

Subject disposition is important in clinical trials as it provides information on the success of the study in terms of recruitment and retention. It also allows for the assessment of potential biases and the

generalizability of the results. Therefore, in the ALERT trial, careful monitoring of subject disposition will be conducted to ensure the integrity and validity of the study findings.

4.2 Definition of Populations for Analysis

To ensure that the results of the ALERT trial are relevant to its intended audience, different populations will be used for analysis. The primary analysis will involve all women who gave birth in the 16 participating hospitals, regardless of whether they received the intervention or not. This will be used to assess the impact of the ALERT intervention on early perinatal mortality rates, the primary outcome of the trial.

The secondary analysis will focus on women who received the intervention during their hospital stay. This population will be used to assess the impact of the intervention on other outcomes such as maternal morbidity, hypoxic-ischaemic insults, and neonatal seizures. Additionally, subgroups based on specific covariates, such as birth weight, mode of delivery, time of delivery, and type of outcome, may be analysed to further explore the impact of the intervention on specific populations of interest.

By using multiple populations for analysis, the ALERT trial will provide a comprehensive understanding of the impact of the intervention on different outcomes and populations. This approach ensures that the results are applicable to a broad range of stakeholders in the field of maternal and child health.

4.3 Definition of Sub-Group Population in Different Analyses

In the ALERT trial, sub-group populations are defined based on specific covariates, such as birth weight, mode of delivery, time of delivery, and type of outcome, in different analyses. These sub-groups are used to investigate the impact of the ALERT intervention on specific populations of interest and to identify potential effect modifiers.

For example, in the primary analysis, the sub-group population will consist of all women who gave birth in the 16 participating hospitals during the study period, regardless of whether they received the ALERT intervention. This population will be used to assess the impact of the intervention on early perinatal mortality rates.

In the secondary analysis, the sub-group population will include all women who received the ALERT intervention during their hospital stay. This population will be used to evaluate the impact of the intervention on secondary outcomes such as maternal morbidity, hypoxic-ischaemic insults, and neonatal seizures.

Additional sub-group analyses may be conducted based on specific covariates of interest, such as birth weight or mode of delivery. These sub-groups will allow for a more detailed examination of the impact of the intervention on specific populations of interest and to identify potential effect modifiers that may impact the efficacy of the ALERT intervention.

Overall, the use of sub-group populations in different analyses will provide a more nuanced understanding of the impact of the ALERT intervention on different populations and outcomes, which can inform the development of targeted interventions for improved maternal and child health outcomes.

4.4 Efficacy Evaluable in the ALERT trial

In the ALERT trial, the efficacy evaluable population will be defined as all women/hospitals who received the ALERT intervention and completed the study as per protocol. This population will be used to evaluate the efficacy of the intervention in improving perinatal outcomes.

To be considered as efficacy evaluable, participants must have received the intervention in full, without any major deviations from the study protocol, and completed the study follow-up assessments. This approach will ensure that the population analysed reflects the impact of the intervention as intended.

The efficacy evaluable population will be used in the secondary analysis of the trial, which will evaluate the impact of the ALERT intervention on outcomes such as maternal morbidity, hypoxic-ischaemic insults, and neonatal seizures. This analysis will provide important information on the potential benefits of the intervention in improving maternal and neonatal health.

Overall, the efficacy evaluable population in the ALERT trial is critical in determining the effectiveness of the intervention and ensuring that the results of the trial are reliable and generalizable to the intended population.

4.5 Intent-to-treat (ITT)

The intent-to-treat (ITT) analysis is a statistical method used in clinical trials that evaluates the effectiveness of an intervention in real-world conditions by analyzing all randomized participants, regardless of their adherence to the protocol or treatment received. In the ALERT trial, the ITT analysis will be used to assess the impact of the ALERT intervention compared to standard care. This method provides a more realistic estimate of the intervention's effectiveness in a real-world setting, but may underestimate the treatment effect as it includes all randomized participants, including those who did not receive the intended treatment or dropped out of the study.

4.6. Study Sites and Populations

The ALERT trial, which began in April 2021, purposefully selected four hospitals in each country through consultations with the respective country's Ministry of Health (refer to Figure 4). All selected hospitals met the following criteria: (i) a minimum caseload of 2,500 births per year, based on trial sample size calculation; (ii) availability of caesarean section and blood transfusion services; (iii) preferably located in rural districts; and (iv) consisting of a mix of typical public but also private-not-for-profit (faith-based) hospitals. The inclusion of public and private-not-for-profit hospitals aimed to reflect the typical hospital landscape in sub-Saharan Africa and improve the generalizability of our results.

Figure 4: Map showing the ALERT trial countries and key indicators for the study hospitals

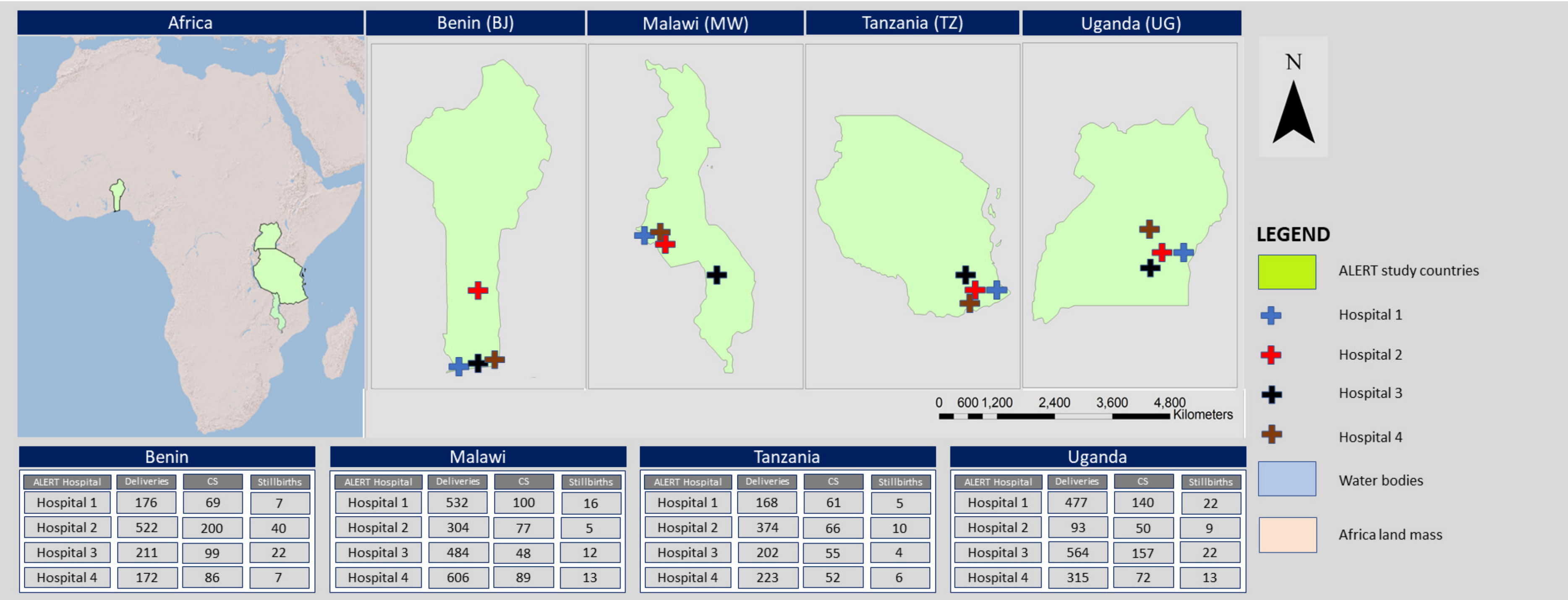

Benin

ALERT Hospital	Deliveries	CS	Stillbirths
Hospital 1	176	69	7
Hospital 2	522	200	40
Hospital 3	211	99	22
Hospital 4	172	86	7

Malawi

ALERT Hospital	Deliveries	CS	Stillbirths
Hospital 1	532	100	16
Hospital 2	304	77	5
Hospital 3	484	48	12
Hospital 4	606	89	13

Tanzania

ALERT Hospital	Deliveries	CS	Stillbirths
Hospital 1	168	61	5
Hospital 2	374	66	10
Hospital 3	202	55	4
Hospital 4	223	52	6

Uganda

ALERT Hospital	Deliveries	CS	Stillbirths
Hospital 1	477	140	22
Hospital 2	93	50	9
Hospital 3	564	157	22
Hospital 4	315	72	13

4.7 Major Protocol Deviations

In any clinical trial, it is essential to adhere to the study protocol to ensure the validity and reliability of the results. However, sometimes deviations from the protocol may occur due to unforeseen circumstances or human error. In the ALERT trial, major protocol deviations will be defined as any deviation that affects the primary outcome or the safety of the participants.

Examples of major protocol deviations may include but are not limited to the following:

- Administration of the ALERT intervention to a non-intervention hospital
- Failure to administer the ALERT intervention in an intervention hospital
- Early termination of the intervention period
- Failure to obtain informed consent from participants
- Major errors in data collection or analysis

Any major protocol deviations will be reported to the trial steering committee, and appropriate action will be taken to address the deviation and mitigate its impact on the trial results. The impact of major protocol deviations on the primary outcome and safety of the participants will be assessed through sensitivity analyses.

5. STATISTICAL ANALYSIS

5.1. Outcome measures

The outcomes of the ALERT trial will be measured for all admitted mothers who deliver in the study hospitals and their pregnancy outcomes (live births, stillbirths, neonatal deaths) that have recorded in the e-registry since March 2021 when the first hospitals

5.2. Analytical approaches

The Makerere University School of Public Health and Karolinska Institutet, in collaboration with the London School of Hygiene & Tropical Medicine, will conduct statistical analysis of primary and secondary outcomes using Stata Standard Edition 17 and R statistical and programming software. The analysis will follow an "intention-to-treat" approach, comparing ALERT intervention clusters (hospital maternity wards) with comparison clusters where care is provided according to national standards.

Descriptive statistics

We will report the number of records, completed records, admissions, and status, as well as mode of delivery and birth outcomes by hospital per time period (refer to dummy table 2).

In this study, we will employ interrupted time series analysis to assess trends in the perinatal registry data collected over a 30-month period. We will present maternal characteristics and outcomes for each hospital, grouped according to exposure (control vs ALERT intervention). While we anticipate no differences in the demographic characteristics of mothers admitted to each hospital during the study period, we expect changes in the number of admissions and outcomes recorded at each hospital as a result of the intervention. We hypothesize that the ALERT interventions will lead to a reduction in adverse pregnancy outcomes, as they are intended to enhance service provision and data quality at the hospitals.

As each hospital operates within a unique population mix, differences may arise within the hospital depending on when they were randomized to the intervention. To account for potential imbalances across hospitals and sequences, we will adjust demographic covariates, although we may not test for demographic differences between the control and intervention groups.

We will present categorical attributes as frequency and percentage, and continuous attributes as mean and standard deviation or median and interquartile range, based on their distribution (see dummy table 3). Additionally, we will present primary outcomes graphically by month (see dummy figure 5), and secondary outcomes will be presented as tables or figures.

Table 2: Example of how number of admissions and data completeness could be presented

Hospital	Total number of records	No. completed records	Referral status and state at admission				Mode of delivery							Birth outcomes			
			Not referred	Yes – not in labour	Yes – in labour	Yes – after birth	SVD	C/S	Vacuum	Forceps	Breech	Singleton	Multiple births	Live birth	Fresh stillbirth	Macerated stillbirth	Neonatal deaths
Benin<br>Hospital 1<br><br>Sequence 1<br>Sequence 2<br>Sequence 3<br>Sequence 4<br>Hospital 2<br><br>Sequence 1<br>Sequence 2<br>Sequence 3<br>Sequence 4<br>Etc.																	
Malawi<br>Hospital 1<br><br>Sequence 1<br>Sequence 2<br>Sequence 3<br>Sequence 4<br>Hospital 2<br><br>Sequence 1<br>Sequence 2<br>Sequence 3<br>Sequence 4<br>Etc.																	

Table 2

Hospital	Variable	Control Arm	Treatment Arm
Benin			
Hospital 1	• Number of records<br>• Age<br>• Status at admission<br>• 1st Stage of labour mgmt<br>• Mode of delivery<br>• PNC checks<br>• Initiation of breast feeding<br>• Length of stay in hospital<br>• N stillbirths<br>• N livebirths<br>• N neonatal deaths<br>Etc.		
Hospital 2			
Hospital 3			
Hospital 4			

Figure 5 – Example of graphical presentation of of monthly mortality rates for Jinja regional referral hospital

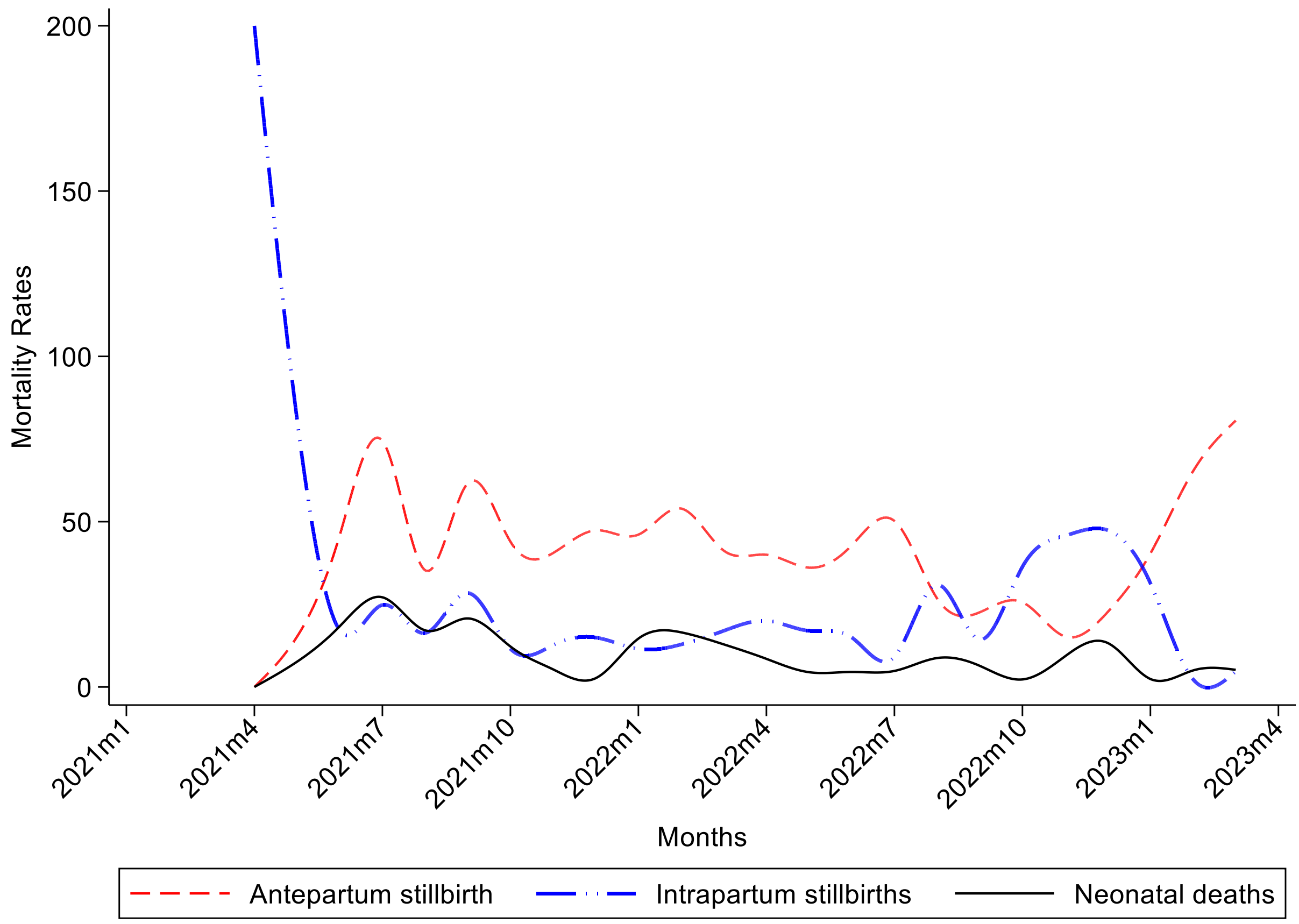

Multivariable analysis

The study will include an analysis of seasonal variations in birth weight and neonatal mortality rates. While stillbirths and early neonatal mortality have declined slowly in the past, we anticipate annual declines of at least 2%. We will examine secular trends across different strata, including countries and hospitals, and estimate the degree of variation in effects across clusters, as recommended by Hemming et al.

GEE is a statistical method that can be used to account for correlated data, such as repeated measures or clustered data. In the case of the ALERT trial, GEE may be a useful approach to account for the correlation between outcomes within the same hospital and to adjust for potential time-trends or other cluster-level factors that may impact the outcomes.

One potential challenge in the use of GEE is the limited number of clusters and sequences in the ALERT trial. To address this, the analysis will initially focus on within-period and within-cluster comparisons to account for the small sample size and clustering. This may involve comparing outcomes within the same hospital and period, as well as conducting subgroup analyses based on relevant covariates.

Once the within-cluster analyses have been completed, GEE can be used to model the relationship between the intervention and outcomes while adjusting for clustering, time-trends, and other relevant factors. This approach may improve the precision of the estimates and help to account for potential confounding factors, such as changes in hospital policies or staffing over time.

Model building using GEE -

The estimate of the mortality risk for the ALERT intervention effect (control vs intervention arms) with their 95% confidence intervals shall be presented for all models fitted.

Models shall be fitted using the following numerical notations

I clusters (i=1, 2, 3, 4, 5, 6, 7, 8, 9, 10, 11, 12, 13, 14, 15, 16)
M time periods (j=1, 2, 3, 4, 5)
N outcomes i.e., stillbirths (k=1, 2 …..N), sampled per cluster per time point (cross-sectional cohort)
Treatment indicator (T_{ij}), equals 1 if intervention present at cluster I at time J, else it is 0
A fixed treatment effect (ϑ)
Fixed time effect (γ_j) (one parameter if calendar time used as continuous variable, otherwise vector)
Fixed effects [β] for patient-level demographics
Patient-level adjustment variables [X_k]
Random cluster effect (α_i)
Residual noise (ε_{ijk})

Model 1 – Shall be fitted with an unadjusted before and after analysis of the effect of the intervention. For this model, the time effect shall be ignored.

$$Logit\ (Y_{ik}) = \vartheta * T_i + \alpha_i + \varepsilon_{ik} \quad (1)$$

Where Y_{ik} denotes the probability of the outcome for k^{th} individual in the ith cluster in the mth period

ϑ denotes the log odds for the treatment/intervention variable (1= treatment, 0=control) in i^{th} cluster

<u>Mode 2</u> – Shall be fitted with adjusted time period (step) to enable us to examine whether any potential ALERT intervention effect is related only to the intervention or also to an independent effect of calendar time

$$Logit(Y_{ijk}) = \vartheta^* T_{ij} + \gamma_j + \alpha_i + \varepsilon_{ijk} \quad (2)$$

Where γ denotes the log odds for the effect of Time (vector if effect not linear)

The use of calendar time as a variable in the analysis poses a risk of confounding, as there may be other factors or events (such as changes in hospital practices or updates to Ministry of Health guidelines) that could impact the outcome measure in both the control and exposed arms.

The impact of this phenomenon may vary from being non-existent or gradual (slowly developing trend) to sudden (rapid and immediate adoption of a new approach that produces a powerful effect). To account for these variations, the model will initially incorporate time as a categorical variable, followed by a linear variable, and the most suitable fitting will be selected accordingly.

<u>Model 3</u> - Shall be fitted with adjusted patient-level attributes

$$Logit(Y_{ijk}) = \vartheta^* T_{ij} + \gamma_j + [\beta]X_{ijk} + \alpha_i + \varepsilon_{ijk} \quad (3)$$

Where β denotes the log odds for matrix of X covariates for the kth episode in the ith hospital and the jth time.

In the statistical analysis, we plan to adjust for potential confounding factors by including covariates such as age at hospitalisation (which may be treated as a continuous variable or grouped by age-range as appropriate), gestational age at admission or birth, previous antenatal care history, mode of delivery, time of delivery, referral status, among others. This adjustment will help us to isolate the effect of the ALERT intervention from other factors that could influence maternal and neonatal outcomes. We will also explore potential effect modification by subgroup analyses based on relevant characteristics, such as maternal age and parity.

<u>Model 4</u> - Model 4 will incorporate time as a treatment effect modifier to assess how the impact of the ALERT intervention changes over time. This will enable us to determine the time required to observe a full-size effect of the intervention on the primary outcome and whether the size of the effect is maintained over time. We will fit the model using interrupted time series analysis, with time as a continuous variable measured as time since exposure to the intervention. In addition, we will include covariates to adjust for potential confounding factors that may affect the outcome.

$$Logit(Yijk) = \vartheta^* Tij + \gamma j + [\beta]Xijk + \omega Qij + \alpha i + \varepsilon ijk \quad (4)$$

This equation 4 represents a model for examining the interaction between time and treatment (variable Q) on the log odds of the outcome for a given hospital (I) at a specific time (J). The variable Q will be analyzed as a numerical variable, where 0 represents any control period and 1, 2, etc. represent the first, second, and subsequent exposure steps of the intervention. The variable ω represents the log odds of this interaction, and it will be fitted as both a continuous and categorical

variable to determine the most appropriate fitting. This model will help us understand how the impact of the intervention changes over time and whether the effect size is maintained over time

Sub-group analysis

Sub-group analysis will be conducted to investigate the effect of the intervention on specific sub-groups of interest, such as stratification by birth weight, mode of delivery, time of delivery, and type of outcomes. The decision to conduct sub-group analysis will depend on the power and sample size of the study, as well as the plausibility of the sub-groups being important for clinical or policy decision-making. These sub-group analyses will be conducted using appropriate statistical methods and will be interpreted cautiously, considering the potential for chance findings and the need for replication in future studies.

5.3 Pooling of Sites

Pooling of sites refers to the process of combining data from multiple study sites or clusters to increase the power and generalizability of the results. In the ALERT trial, pooling of sites will be used to increase the statistical power of the study and to obtain more precise estimates of treatment effects.

Pooling of sites will involve combining data from the 16 participating hospitals to create a larger sample size. This will improve the precision of the estimated treatment effect, reduce the likelihood of type II errors (false negative results), and increase the generalizability of the findings to a broader population of women and infants.

However, pooling of sites can also introduce heterogeneity across sites, which can affect the internal validity of the study. To address this, the ALERT trial will use appropriate statistical methods, such as random effects models, to account for the potential heterogeneity between sites.

In summary, pooling of sites in the ALERT trial will provide several benefits, including increased power and generalizability of the results. However, it is important to carefully consider and account for any potential heterogeneity between sites in the analysis to ensure the validity of the study results.

5.4 Interim Analyses

Interim analyses are pre-planned statistical evaluations of accumulating data during a clinical trial to assess the efficacy and safety of the intervention. In the ALERT trial, interim analyses will be conducted to evaluate the safety and efficacy of the ALERT intervention at predetermined time points. The interim analyses will be conducted by an independent Data and Safety Monitoring Board (DSMB), which is an independent group of experts responsible for monitoring the safety and efficacy of the trial.

The DSMB will review the accumulating data from the trial and assess whether there are any safety concerns or if there is enough evidence to demonstrate the effectiveness of the ALERT intervention. The interim analyses will also evaluate the potential benefits and harms of continuing or stopping the trial early.

The ALERT trial will conduct two interim analyses: one when approximately 50% of the total sample size is reached and another when approximately 75% of the total sample size is reached. The interim analyses will be conducted using pre-specified stopping rules and statistical criteria.

The use of interim analyses in the ALERT trial ensures that the trial is conducted in an ethical manner and that the safety and efficacy of the intervention are carefully monitored. If the intervention is found to be significantly effective, the trial may be stopped early to allow for earlier implementation of the intervention in clinical practice. Conversely, if the intervention is found to be harmful or ineffective, the trial may be stopped early to prevent further exposure of participants to the intervention.

5.5 Time-Points For Analysis

The ALERT trial will analyse outcomes at several time-points, including baseline, during intervention, post-intervention, and follow-up. Data will be collected at regular intervals to evaluate the impact of the intervention on primary and secondary outcomes such as perinatal mortality rates and maternal morbidity. The timing of data collection will vary depending on the specific outcome being evaluated. The aim of analysing outcomes at multiple time-points is to gain a comprehensive understanding of the short-term and long-term impact of the ALERT intervention on maternal and neonatal outcomes

5.6 Methods for Handling Missing Data

In the ALERT trial, missing data can occur due to various reasons, such as hospital withdrawal, or incomplete data collection. To address this issue, several methods will be employed to handle missing data, including:

1. **Complete case analysis**: In this method, only the data from participants with complete data at a given time-point will be included in the analysis. This method is simple but may lead to biased results if the missing data is not missing completely at random.
2. **Multiple imputation**: This method involves imputing missing data using statistical models based on the observed data. Multiple imputations are created, and the analysis is performed on each imputed dataset, and the results are combined. This method is more robust than complete case analysis and can handle missing data that is not missing completely at random.
3. **Last observation carried forward (LOCF):** In this method, missing data is imputed using the last observed value. This method is simple but may lead to biased results if the missing data is not missing at random.
4. **Sensitivity analysis:** Sensitivity analysis involves assessing the impact of missing data on the results by performing different analyses under different assumptions about the missing data. This method allows the researchers to evaluate the robustness of the study findings to missing data.

The choice of method for handling missing data will depend on the type and extent of missing data, the outcome of interest, and the assumptions about the missing data mechanism. By using multiple methods to handle missing data, the ALERT trial aims to ensure that the analysis is robust and that the results are not biased by missing data.

STATISTICAL ANALYSIS PLAN

ALERT

ACTION LEVERAGING EVIDENCE TO REDUCE PERINATAL MORTALITY AND MORBIDITY IN SUB-SAHARAN AFRICA (ALERT) TRIAL

The golf**First draft version**

Name	Signature	Date
Professor Claudia Hanson		20 Feb 2022
Professor Nicola Orsini		10th Feb 2022

DRAFT DEVELOPED BY:

Name	Signature	Date
Dr. Joseph Akuze		2022/02/01

FINAL VERSION APPROVED BY:

Name	Signature	Date
Professor Claudia Hanson		10th Nov 2025
Professor Nicola Orsini		10th Nov 2025

DRAFT DEVELOPED BY:

Name	Signature	Date
Dr. Joseph Akuze		2025/11/03

**VISION HISTORY CHRONOLOGY:**		
**VERSION NUMBER**	**EFFECTIVE DATE**	**REVIEWED BY (NAME)**
V1	2022/02/01	Claudia Hanson, Peter Waiswa, Joseph Akuze and DSMB
V2	2023/03/23	Claudia Hanson, Peter Waiswa, Joseph Akuze and DSMB
V3	2023/06/01	Claudia Hanson, Peter Waiswa, Joseph Akuze and DSMB
V4	2023/09/01	Claudia Hanson, Peter Waiswa, Joseph Akuze and DSMB
V5<br>(without extension)	2024/05/01	Claudia Hanson, Peter Waiswa, Joseph Akuze and DSMB<br>**New: Alternative Time Specifications**: Multiple time period definitions will be tested including monthly and quarterly groupings
V6	2025/07/01	Claudia Hanson, Peter Waiswa, Joseph Akuze and DSMB

Table of Contents

1. STUDY BACKGROUND 1
2. STUDY OBJECTIVES 1
2.1 Specific objective 1
2.2 Assessment of objectives 1
3. STUDY DESIGN 4
3.1 Overview 4
3.2 Sample Size 4
3.3 Randomization and blinding 4
Figure 3a: Randomization syntax file 5
3.4 Study assessments 6
4. STUDY POPULATIONS 6
4.1 Definition of populations for analysis 6
4.2 Intent-to-treat (ITT) 6
4.3. Study sites and populations 7
4.4 Major protocol deviations 9
5. STATISTICAL ANALYSIS 9
5.1. Study outcomes and indicators 9
5.2. Analytical approaches 10
Data preparation 10
Descriptive analysis approach 10
Hospital-specific mixed-effects analysis 14
5.3 Statistical Inference 18
5.4 Missing data management 18
5.5 Implementation 18
Annex: DSMB composition and tasks 19

1. STUDY BACKGROUND

The Action Leveraging Evidence to reduce perinatal mortality and morbidity in Sub-Saharan Africa (ALERT) trial was set to develop and evaluate a comprehensive and multilevel intervention covering intrapartum care and midwifery. The ALERT trial and interventions targeted hospital maternity units and included i) end-user participation of women, families, and midwifery providers to co-design the intervention; ii) in-service midwifery competency-based training; iii) empowerment and leadership mentoring of maternity unit leaders, and iv) quality improvement in the maternity ward, supported by district-based bi-annual coordination and accountability meetings (Fig. 1).

Figure 1: ALERT Conceptual Framework

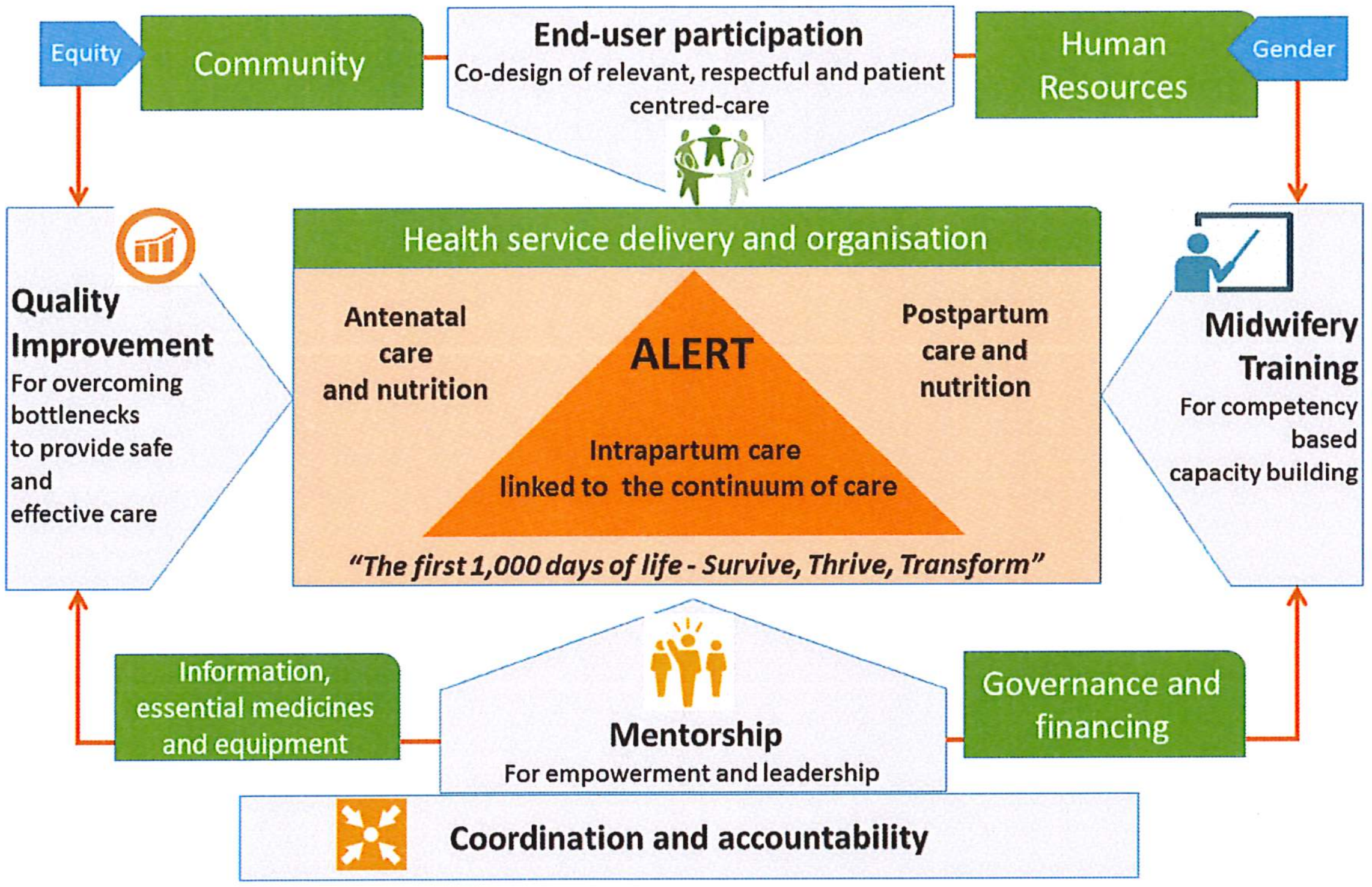

2. STUDY OBJECTIVES

2.1 Specific objective

The ALERT trial had three specific study objectives:

1. To assess the ALERT intervention's impact in hospital maternity units on perinatal and maternal health outcomes, including women's experience of care.
2. To evaluate the process of implementation of the intervention to understand what works for whom and under what circumstances.
3. To conduct a cost-effectiveness analysis of the ALERT intervention.

2.2 Assessment of objectives

A summary of our approach to answering the objectives is given in Table 1. In this Analysis Plan, we give details for only objective one (details about other study objectives are given elsewhere).

Table 1: Methods and data sources by study objective

Objective	Methods	Data Source	Notes and links
Obj 1. To assess the ALERT intervention's impact in hospital maternity units on perinatal and maternal health outcomes, including women's experience of care.	Trial profile<br>Assessment of recruitment rate, engagement into the planned intervention e.g hospital engagement score and records completeness/incompleteness<br>- Number of records entered the REDCap database in comparison to expected number of records (i.e. HMIS)<br>- Number of incomplete records<br>Descriptive statistics (counts, proportions, mean, median, SD, max, min)<br>– Study outcomes split by intervention arm<br>– Additionally, we will summarize the primary outcome split by both site and by intervention condition<br>Regression models fitted for the primary, secondary and process outcomes.<br>– Hospital-specific mixed-effects logistic regression models fitted separately for each hospital with random intercepts for randomization groups, followed by random-effects meta-analysis<br>– Sensitivity analyses conducted by excluding outlier hospitals with data quality concerns<br>Women's experiences of care<br>- Descriptive statistics for each hospital, country (means and proportions)<br>- Distribution of the of optimal care and number of interventions received.<br>- Regression models to predict factors that determine optimal women's care	Perinatal e-registry<br>Exit interviews	Our co-primary outcomes are Fresh stillbirth rate and in-facility early perinatal mortality (composite outcome).<br><br>In-facility early perinatal mortality is defined as in-facility (fresh) stillbirth and 24-hour neonatal mortality

Objective	Methods	Data Source	Notes and links
	- Spatial analysis? – Health seeking behaviors vis-a-viz care seeking		
Obj 2. To evaluate the process of implementation of the intervention to understand what works for whom and under what situation.	Trial profile<br>- Table1: fidelity and implementation strength (from the REDCap fidelity data base)<br>- Table2: Implementation in the hospital – to be developed and will include the improvement topics and change ideas by hospital.<br>- Realist evaluation: Strengthening capacity in hospitals to reduce perinatal morbidity and mortality through a codesigned intervention package: protocol for a realist evaluation as part of a stepped-wedge trial of the Action Leveraging Evidence to Reduce perinatal morTality and morbidity (ALERT) in sub-Saharan Africa project - PubMed	WP7 – [WP7 published separate protocol]	
Obj 3. To conduct a cost-effectiveness analysis of the ALERT intervention.	Cost effectiveness analysis<br>- Provider's perspectives<br>- Effects per outcomes<br>- Incremental cost-effectiveness ratio (ICER)<br>- Sensitivity analysis	Costing data	

3. STUDY DESIGN

3.1 Overview

A stepped-wedge cluster-randomized trial (SWCRT) design with a nested process evaluation based on realist evaluation was employed in the ALERT trial. This design was selected as the interventions were administered at the hospital level, and a stepped-wedge design was chosen to mirror scale-up for policy buy-in and for statistical efficiency as larger cluster-level differences are anticipated.

3.2 Sample Size

The ALERT trial was carried out in 16 hospitals, with four hospitals selected in each of the four countries: Benin, Malawi, Tanzania, and Uganda. The selection criteria for the hospitals required them to have a minimum of 2500 births per year, which provided sufficient statistical power (75%-80%) to detect a 25% reduction in the in-facility early perinatal mortality rates with a 95% confidence interval. This also allowed for the assessment of other secondary outcomes such as maternal morbidity, hypoxic-ischemic insults, neonatal seizures, and more (see Figure 2).

Figure 2: Sample size and Power calculations for ALERT trial outcomes

	Baseline estimates	expected change	expected value	ICC	Power
**Perinatal e-registry (Cluster size 1500 births)**					
Stillbirth	1.0%	30%	0.7%	0.0024	83%
Stillbirth	1.0%	30%	0.7%	0.024	82%
Perinatal mortality	2.0%	25%	1.5%	0.0024	93%
Perinatal mortality	2.0%	25%	1.5%	0.024	92%
Perinatal mortality	1.4%	25%	1.1%	0.0024	70%
Hypoxic-ischaemic insults	4.0%	30%	2.8%	0.0024	100%
Hypoxic-ischaemic insults (sub-group)	4.0%	30%	2.8%	0.0024	79%
Neonatal seizures	0.5%	50%	0.3%	0.0024	78%
Ceasarean section	15.0%	20%	18.0%	0.01	100%
Maternal morbidity	3.0%	30%	2.1%	0.5	99%
**Interviews post-partum (Cluster size 50 interviews)**					
Responsiveness	70.0%	15%	80.0%	0.1	97%
Mistreatment	4.0%	75%	1.0%	0.1	77%
Breastfeeding	60.0%	20%	71.0%	0.1	98%

Source: *Additional file 4 - **Action** leveraging evidence to reduce perinatal mortality and morbidity (ALERT): study protocol for a stepped-wedge cluster-randomised trial in Benin, Malawi, Tanzania and Uganda*

3.3 Randomization and blinding

The ALERT trial employed randomization, which was carried out by an independent statistician after obtaining consent from the participating hospitals. Stratification by country was used to enable random selection of four hospitals in each country (refer to randomization syntax in Figure 3a). Participants (hospitals) will not be blinded to the intervention, as with all training and quality improvement interventions. However, the women and their families receiving care at these hospitals will not be aware of the exact implementation stage of ALERT. The final sequence of ALERT intervention and data collection is illustrated in Figure 3b.

Figure 3a: Randomization syntax file

```
/*

This do-file is showing how to create a file with the randomized sequence
Date: 20200617
Author: Orsini N
*/

* Enter the country and hospital

clear
input   str10 state  code str50  name
        Benin       1         "Centre National Hospitalier Hubert Maga"
        Benin       2    "Hopital de Zone Come-Grand Popo Houeyogbe Bopa"
        Benin       3                       "Policlinique Bon Samaritan"
        Benin       4         "Centre Hospitalier et Dépt Zou-Collines"
       Malawi       1                                "Mchinji Hospital"
       Malawi       2                          "Mitundu Rural Hospital"
       Malawi       3                               "Ntcheu D Hospital"
       Malawi       4                            "St Gabriels Hospital"
     Tanzania       1                        "Newala district Hospital"
     Tanzania       2                              "Mkomaindo Hospital"
     Tanzania       3                                 "Ndanda Hospital"
     Tanzania       4                             "Nachingwea Hospital"
       Uganda       1                         "Bugiri General Hospital"
       Uganda       2                        "Iganga Referral Hospital"
       Uganda       3                         "Jinja Regional Hospital"
       Uganda       4                         "Kamuli Mission Hospitla"
end

ssc install shuffle
gen rand = .

* Set the seed to reproduce the randomization
set seed 20200617

local list = "Benin Malawi Tanzania Uganda"
qui foreach s of local list {
        local j = 1
        shuffle 1 2 3 4, num noisily
        local seq = r(list)
            foreach r of local seq {
                    replace rand = `r' if code == `j' & state == "`s'"
                    local j = `j' + 1
            }
}
export excel using "results_rand_200617", firstrow(variables) replace
```

Figure 3b: Sequence of the ALERT trial intervention

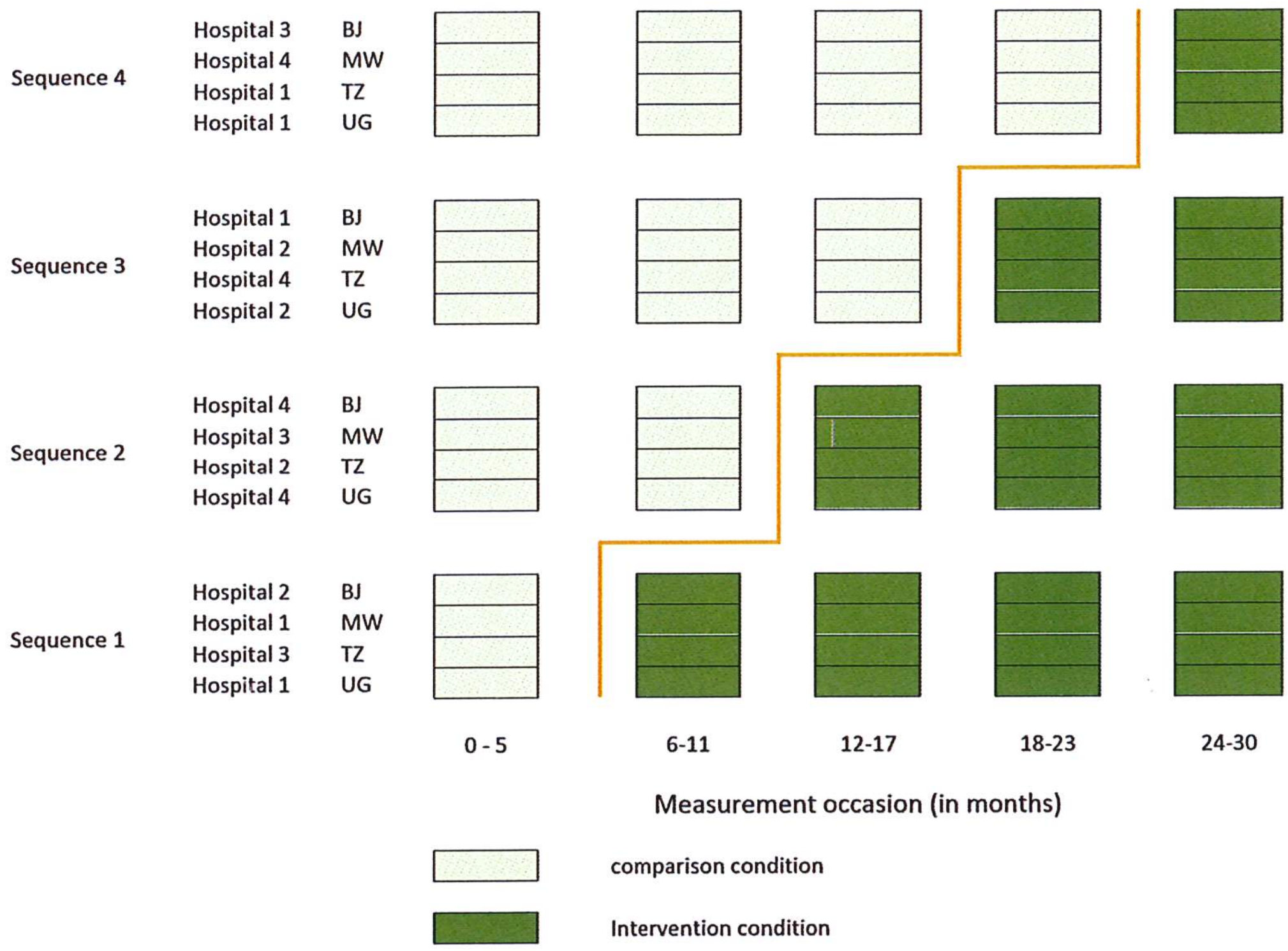

3.4 Study assessments

To achieve objective one, we will conduct two assessments. Firstly, we will abstract trial data from hospital registries and enter it into a REDCap application. Secondly, we will conduct exit interviews with hospital clients to capture and evaluate their care-related experiences while in the hospitals.

4. STUDY POPULATIONS

4.1 Definition of populations for analysis

To ensure that the results of the ALERT trial are relevant to its intended audience, different populations will be used for analysis. The primary analysis will involve all women who gave birth in the 16 participating hospitals, regardless of whether they received the intervention or not. This will be used to assess the impact of the ALERT intervention on the primary and secondary outcomes and the process.

4.2 Intent-to-treat (ITT)

The intent-to-treat (ITT) analysis is a statistical method used in clinical trials that evaluates the effectiveness of an intervention in real-world conditions by analyzing all randomized participants, regardless of their adherence to the protocol or treatment received. In the ALERT trial, the ITT analysis will be used to assess the impact of the ALERT intervention compared to standard care. This method provides a more realistic estimate of the intervention's effectiveness in a real-world setting, but may underestimate the treatment effect as it includes all randomized participants, including those who did not receive the intended treatment, or received late or dropped out of the study.

4.3. Study sites and populations

The ALERT trial, which began its preparatory work in 2020, purposefully selected four hospitals in each country through consultations with the respective country's Ministry of Health (refer to Figure 4). All selected hospitals met the following criteria: (i) a minimum caseload of 2,500 births per year, based on trial sample size calculation; (ii) availability of cesarean section and blood transfusion services; (iii) preferably located in rural districts; and (iv) consisting of a mix of typical public but also private-not-for-profit (faith-based) hospitals. The inclusion of public and private-not-for-profit hospitals aimed to reflect the typical hospital landscape in sub-Saharan Africa and improve the generalizability of our results.

Figure 4: Map showing the ALERT trial countries and key baseline indicators for the study hospitals

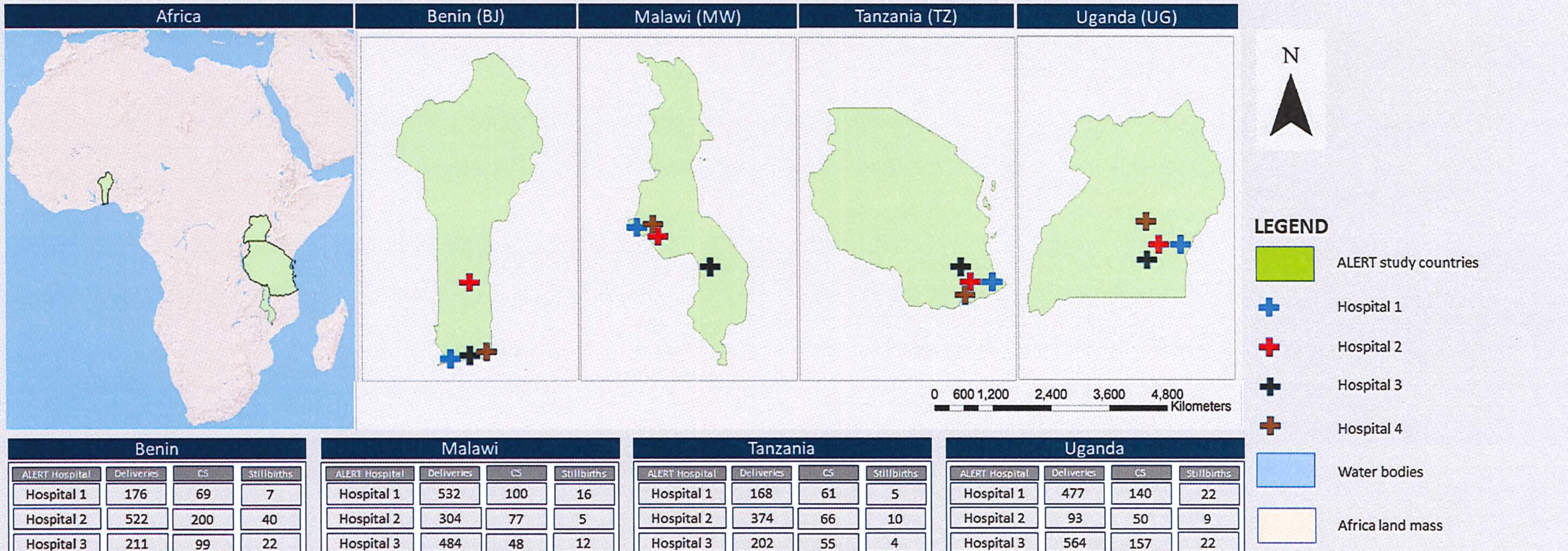

Benin

ALERT Hospital	Deliveries	CS	Stillbirths
Hospital 1	176	69	7
Hospital 2	522	200	40
Hospital 3	211	99	22
Hospital 4	172	86	7

Malawi

ALERT Hospital	Deliveries	CS	Stillbirths
Hospital 1	532	100	16
Hospital 2	304	77	5
Hospital 3	484	48	12
Hospital 4	606	89	13

Tanzania

ALERT Hospital	Deliveries	CS	Stillbirths
Hospital 1	168	61	5
Hospital 2	374	66	10
Hospital 3	202	55	4
Hospital 4	223	52	6

Uganda

ALERT Hospital	Deliveries	CS	Stillbirths
Hospital 1	477	140	22
Hospital 2	93	50	9
Hospital 3	564	157	22
Hospital 4	315	72	13

4.4 Major protocol deviations

In any clinical trial, it is essential to adhere to the study protocol to ensure the validity and reliability of the results. However, sometimes deviations from the protocol may occur due to unforeseen circumstances or human error. In the ALERT trial, major protocol deviations will be defined as any deviation that affects the primary outcome or the safety of the participants.

Examples of major protocol deviations may include but are not limited to the following:

- Administration of the ALERT intervention to a non-intervention hospital
- Failure to administer the ALERT intervention in an intervention hospital
- Early termination of the intervention period
- Failure to obtain informed consent from participants
- Major errors in data collection or analysis

Any major protocol deviations will be reported to the trial steering committee, and appropriate action will be taken to address the deviation and mitigate its impact on the trial results. The impact of major protocol deviations on the primary outcome and safety of the participants will be assessed through sensitivity analyses.

5. STATISTICAL ANALYSIS

5.1. Study outcomes and indicators

The outcomes (primary, secondary and process) of the ALERT trial will be measured for all admitted mothers who deliver in the study hospitals and their pregnancy outcomes (livebirths, stillbirths, neonatal deaths) have been recorded in the e-registry from March 2021 to December 2023. The main study outcomes include;

Primary outcomes

1. **Fresh stillbirth rate**: Number of fresh stillbirths per 1,000 live and stillbirths
2. **Early perinatal mortality rate:** Number of fresh stillbirths and 24-hour neonatal deaths per 1,000 live and stillbirths (composite outcome)

Secondary outcomes

3. **Hypoxic-ischemic events:** Number of neonates with APGAR score less than 7 at 5 minutes per 1,000 live- and stillbirths
4. **Neonatal seizures:** Number of neonates diagnosed with seizures per 1,000 live- and stillbirths
5. **Cesarean section rate:** Number of cesarean sections per 100 live- and stillbirths
6. **Severe maternal morbidity:** Number of women with severe morbidities per 100 live- and stillbirths
7. **Responsiveness score:** Validated percentage score from exit interviews
8. **Respectful treatment score:** Validated percentage score measuring absence of mistreatment from exit interviews

Process indicators

9. **Fetal heart check on admission:** Percentage of babies with fetal check completed

10. **Intrapartum monitoring:** Fetal monitoring documented in first stage of labor
11. **Resuscitation for babies with APGAR < 7:** Percentage resuscitated
12. **Companion presence**: Companion present during labor or birth
13. **Breastfeeding within 1 hour**: Breastfeeding initiation within one hour.

5.2. Analytical approaches

The Makerere University School of Public Health and Karolinska Institutet, in collaboration with the London School of Hygiene & Tropical Medicine, will conduct statistical analysis of primary and secondary outcomes using Stata Standard Edition 18. The analysis will follow an "intention-to-treat" approach, comparing ALERT intervention clusters (hospital maternity wards) with comparison clusters where care is provided according to national standards. This approach will account for the complex clustering inherent in our design.

Data preparation

The 30-month study period will be divided into five discrete six-month intervals. Time periods will be defined as:

Stata

```
egen time3 = cut(month), group(5) label
recode month (min/12= 0 "1-6 Months") (13/18=1 "7-12 Months") ///
(19/24=2 "13-18 Months") (25/30=3 "19-24 Months") ///
(31/36=4 "25-30 Months"), gen(time2) label(period)
```

Hospitals will be assigned anonymized identifiers using standardized Country-Hospital-Number format and sequential numerical coding from 1 to 16 for analytical purposes.

Treatment exposure will be constructed to reflect the stepped-wedge design where hospitals transition permanently from control to intervention status:

Stata

```
generate byte has_been_treated = 0
replace has_been_treated = 1 if time3 ==1 & group ==1 ///
| time3 ==2 & inlist(group, 1,2) | time3 ==3 & inlist(group,1,2,3) |
time3 ==4
```

Baby-level analyses will require data reshaping from wide to long format to accommodate multiple births per woman.

Descriptive analysis approach

Baseline characteristics will be presented as frequencies and percentages for categorical variables, means with standard deviations for continuous variables with symmetric and bell-shaped distributions, and medians with interquartile ranges for very asymmetric distributions. Temporal trends will be visualized through monthly outcome rates by hospital to identify intervention effects and secular changes.

Sample Table 1: Distribution of Number maternal records collected from the e-registry

Clinics	Steps (2-month periods) 1	2	3	4	5	6	7	8	9	10	11	Total
1	1886	1280	1041	1249	1208	1336	1223	1181	945	1179	1340	13868
2	3653	2150	2250	2504	2846	2701	2604	2390	2204	2580	2404	28286
3	1360	769	830	664	799	930	872	803	765	731	768	9291
4	3138	2063	2055	1859	1683	2061	2227	2392	1888	1617	1841	22824
5	2775	1928	968	2258	2070	2015	2482	2366	2214	2034	2224	23334
6	2524	1624	1445	1602	1803	1772	1663	1595	1504	1569	1613	18714
7	1516	1293	1321	1310	1382	1476	1406	1343	1265	1357	1378	15047
8	3511	2307	1798	2179	2459	2693	2618	2593	2347	2220	2275	27000
9	2977	1816	1823	1529	1691	1728	1984	2006	1895	1535	1711	20695
10	3148	3634	2569	2730	3638	3652	3974	3983	3772	3941	4177	39218
Total	26488	18864	16100	17884	19579	20364	21053	20652	18799	18763	19731	218277

Control study period   Intervention study period

Table 2: Example of how number of admissions and data completeness could be presented

Hospital	Total number of records	No. completed records	Referral status and state at admission				Mode of delivery							Birth outcomes			
			Not referred	Yes – not in labor	Yes – in labor	Yes – after birth	SVD	C/S	Vacuum	Forceps	Breech	Singleton	Multiple births	Live birth	Fresh stillbirth	Macerated stillbirth	Neonatal deaths
Benin<br>Hospital 1<br><br>Sequence 1<br>Sequence 2<br>Sequence 3<br>Sequence 4<br>Hospital 2<br><br>Sequence 1<br>Sequence 2<br>Sequence 3<br>Sequence 4<br>Etc.																	
Malawi<br>Hospital 1<br><br>Sequence 1<br>Sequence 2<br>Sequence 3<br>Sequence 4<br>Hospital 2<br><br>Sequence 1<br>Sequence 2<br>Sequence 3<br>Sequence 4<br>Etc.																	

Table 2

Hospital	Variable	Control Arm	Treatment Arm
Benin			
Hospital 1	• Number of records<br>• Age<br>• Status at admission<br>• 1st Stage of labor mgmt<br>• Mode of delivery<br>• PNC checks<br>• Initiation of breast feeding<br>• Length of stay in hospital<br>• N stillbirths<br>• N livebirths<br>• N neonatal deaths<br>Etc.		
Hospital 2			
Hospital 3			
Hospital 4			

Figure 5 – Example of graphical presentation of monthly mortality rates for Jinja regional referral hospital

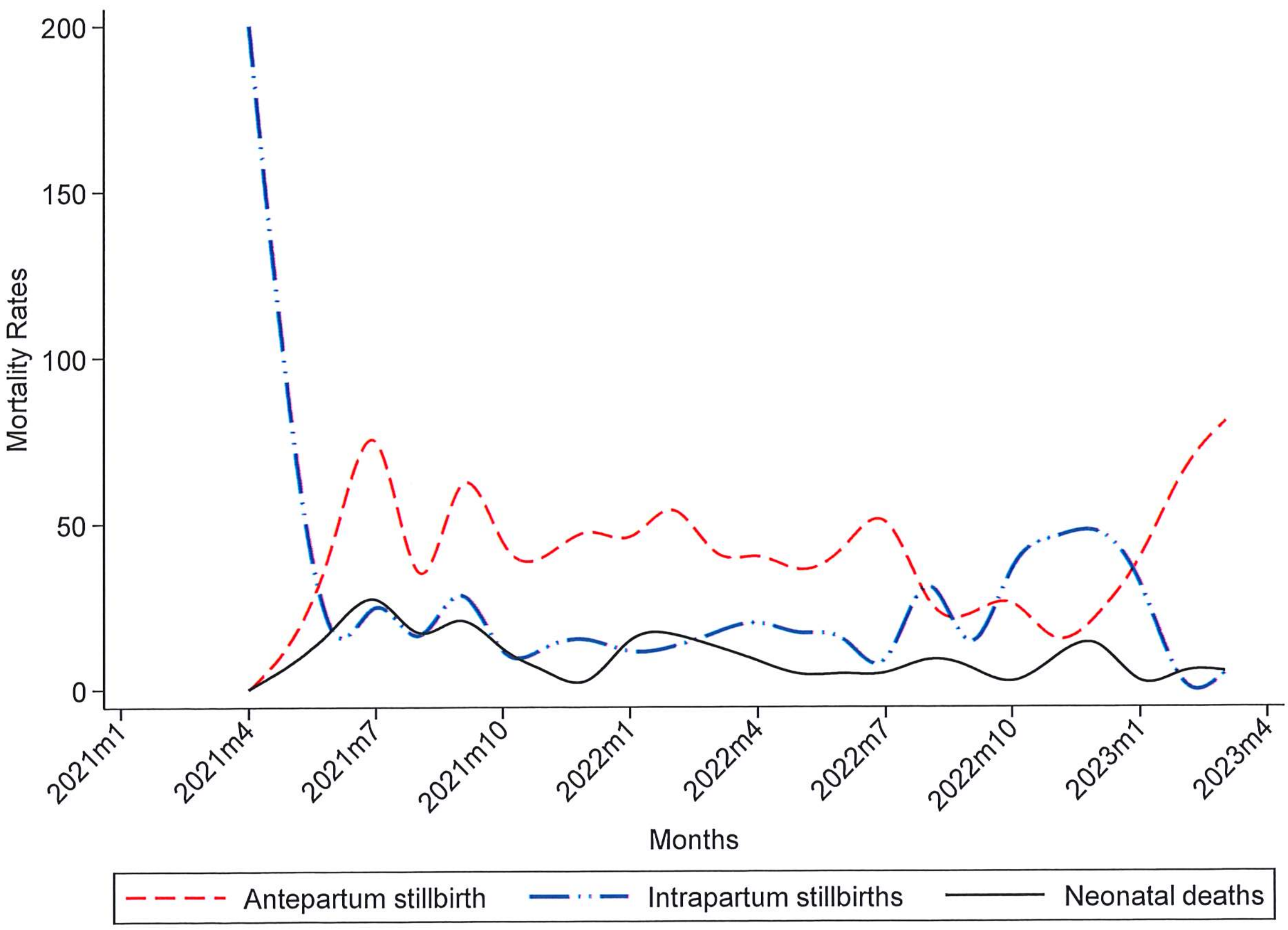

Hospital-specific mixed-effects analysis

The primary analytical approach will employ hospital-specific mixed-effects regression models to account for substantial heterogeneity across hospitals and healthcare contexts. We have chosen hospital-specific mixed-effects modelling over traditional pooled analyses to reflect the substantial heterogeneity between hospitals. We anticipate that hospitals operating in diverse healthcare contexts across four countries. While pooled approaches offer statistical efficiency, they risk masking important effect modification that could inform future implementation strategies.

Our modelling strategy recognizes that intervention effects may vary substantially across settings due to differences in baseline quality, staffing patterns, patient populations, and organizational cultures. By fitting separate models for each hospital, we preserve these nuances while enabling subsequent meta-analysis for overall effect estimation.

Box 1: Changes in methodological approaches

**BOX 1: METHODOLOGICAL CHANGE FROM PREVIOUS VERSIONS (V1-V4)**
**Background:** Statistical analysis plans versions 1-4 (February 2021 - September 2023) specified Generalized Estimating Equations (GEE) as the primary analytical method for accounting for correlated data and clustering.
**Rationale for change to mixed-effects models:** During preliminary analyses conducted in early 2024, GEE models encountered substantial convergence failures and excessive computation times due to the complexity of the ALERT trial's data structure (16 hospitals, 4 countries, stepped-wedge design with varying implementation sequences).
**Decision process:** At the Data Safety and Monitoring Board meeting on May 28, 2024, the analytical team presented these computational challenges. Following discussion and review of methodological literature (particularly Hemming et al.'s guidance on cluster randomized stepped-wedge trials), the DSMB agreed to adopt mixed-effects regression models as the primary analytical approach.
**Advantages of mixed-effects models:** Enhanced computational tractability with reliable convergence, greater granularity in modeling hospital-level variation, appropriate handling of the nested data structure, and maintained interpretability of treatment effects while accounting for clustering and time trends.

Binary Outcome Specification

For binary outcomes such as stillbirths, seizures, and cesarean sections, we will employ logistic mixed-effects models within each hospital. The mathematical specification follows:

<u>Model 1 – Logistic Mixed-Effects</u>

$\text{logit}(P(Y\_ijk = 1)) = \beta_0 + \beta_1 T\_ij + \beta_2 Time\_ij + \beta_3 Group\_i + \alpha\_i$

Where:

- Y_ijk = binary outcome for individual k in hospital i at time j
- T_ij = treatment indicator (1 = intervention, 0 = control)
- $Time\_ij$ = time period effect
- $Group\_i$ = randomization group fixed effect
- α_i = random intercept for randomization group

The Stata implementation will follow this syntax:

Stata

```
melogit outcome i.treat i.time i.group if hospn==j || group:, or
```

Continuous outcome specification

Patient experience scores from exit interviews require different modelling approaches given their continuous nature. We will employ linear mixed-effects models with the following specification:

Model 2 - Linear mixed-effects:

$Y\_ijk = \beta_0 + \beta_1 T\_ij + \beta_2 Time\_ij + \beta_3 Group\_i + \alpha\_i + \varepsilon\_ijk$

Where:

- Y_ijk = continuous outcome for individual k in hospital i at time j
- T_ij = treatment indicator (1 = intervention, 0 = control)
- $Time\_ij$ = time period effect
- $Group\_i$ = randomization group fixed effect
- α_i = random intercept for randomization group
- ε_ijk = individual-level error term

Implementation:

Stata

```
mixed outcome i.treat i.round i.group if hospnam==j || group:, cov(ex)
```

Random effects considerations

Our random effects structure acknowledges the stepped-wedge design's inherent clustering. Random intercepts for randomization groups account for the possibility that hospitals randomized to similar implementation sequences may share unmeasured characteristics influencing outcomes. This approach provides more appropriate inference than fixed-effects alternatives while maintaining computational tractability.

Systematic results management

Managing results across 16 hospitals and multiple outcomes requires systematic data handling. We will employ Stata's postfile functionality to create comprehensive results databases. This approach

will ensure consistent results' capture while handling the convergence issues may that arise in complex mixed-effects models.

Stata

```
tempname memhold
postfile `memhold' hospn treat_beta treat_se pvalue using
"results.dta", replace

forval j=1/16 {

    capture noisily melogit outcome i.treat i.time i.group if hp==`j'
|| group:, or

    if _rc == 0 {

        matrix b = e(b)

        local beta = b[1,2]

        matrix v = e(V)

        local see = sqrt(v[2,2])

        local z = `beta' / `see'

        local pvalue = 2 * (1 - normal(abs(`z')))

        post `memhold' (`j') (`beta') (`see') (`pvalue')

    }

}

postclose `memhold'
```

Meta-analysis

Hospital-specific results will be combined using random-effects meta-analysis techniques employing the DerSimonian-Laird method:

$\hat{\theta}$_pooled = $(\Sigma w_i \hat{\theta}_i) / (\Sigma w_i)$

where $w_i = 1/(\sigma^2_i + \tau^2)$, incorporating within-hospital sampling variance (σ^2_i) and between-hospital heterogeneity (τ^2).

Forest plots will display individual hospital effects alongside pooled estimates. I-squared statistics will quantify heterogeneity, with values above 50% indicating substantial variation requiring investigation.

Sensitivity analyses

Hospital exclusion: Outlier Hospitals identified to have data quality concerns will be excluded to assess result robustness: After preliminary analysis, reviews with country teams will be held to contextualize unexpected patterns in hospital-specific results. Specific issues like governance crises and infrastructure deficits shall be probed and discussed. Decisions about exclusions for hospitals identified as outliers shall not solely be based on statistical outliers but also on contextual issues identified by country implementation teams.

Stata

```
melogit outcome i.treat i.time3 i.group if hospn==j & hospn!=23 ||
group:, or
```

Alternative Time Specifications: Multiple time period definitions will be tested including monthly and quarterly groupings.

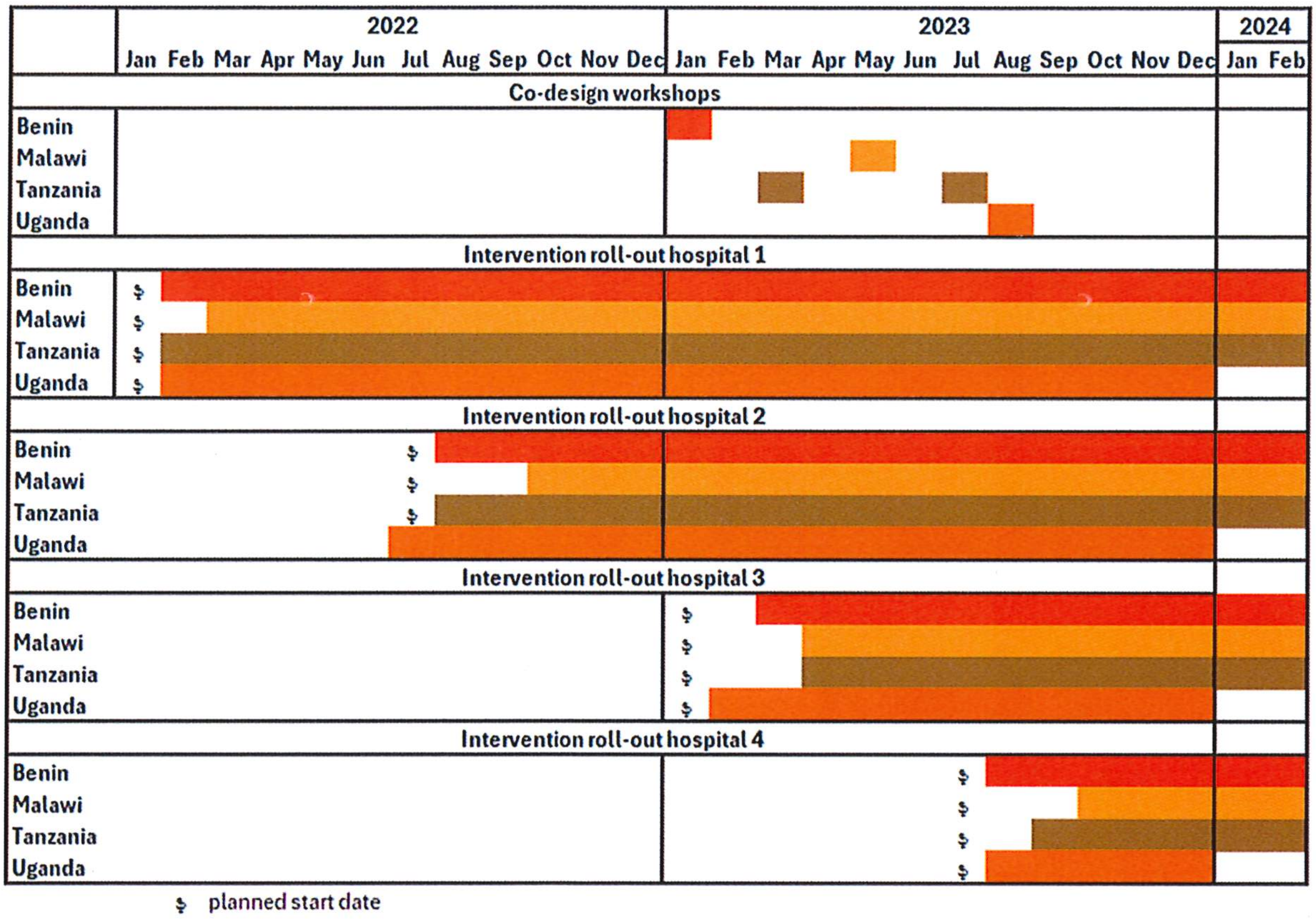

We have accommodated an alternative timeline in line with the actual implementation. All countries faced delays in engaging the hospitals, Uganda comparatively less. Our Per-Protocol analysis used the actual implementation. For per protocol analysis, a new variable reflecting the protocol implementation time shall be used instead of the "time" variable used for the intention to treat analysis (main analysis). Meaning the analysis syntax will essentially remain unchanged for the different outcome types.

Missing Data: Complete case analysis will serve as the primary approach. Multiple imputation will be employed where missingness exceeds 10%:

Stata

```
mi set wide

mi register imputed [varlist]

mi impute chained (logit) binary_vars (regress) continuous_vars =
predictors, add(10)
```

5.3 Statistical Inference

Two-sided hypothesis testing will be employed with $\alpha = 0.05$. Results will be interpreted considering patterns across related outcomes rather than individual p-values given multiple testing considerations.

Effect sizes will be presented as odds ratios with 95% confidence intervals for binary outcomes and mean differences with 95% confidence intervals for continuous outcomes. Forest plots will serve as primary results presentation, displaying individual hospital effects alongside pooled estimates and heterogeneity measures.

5.4 Missing data management

Electronic registry data will have minimal missing data for primary outcomes, warranting complete case analysis. Exit interview data may have higher missing rates requiring multiple imputation for key indicators with substantial missingness. Missing data patterns will be characterized systematically, with sensitivity analyses comparing complete case results with multiple imputation alternatives where missingness exceeds 10%.

5.5 Implementation

All analyses will employ Stata Standard Edition 18 with comprehensive documentation ensuring reproducibility. Results generation will use programmatic approaches with the table1 command for descriptive tables and custom programs for meta-analysis visualizations.

The analytical workflow will follow this structure:

Stata

```
// Data preparation

use allcountries16Apr24clean.dta, clear

[data management procedures]


// Hospital-specific analysis

forval j=1/16 {

    [individual hospital analysis]

}


// Meta-analysis

metan treat_beta treat_se, eform random [options]
```

Annex: DSMB composition and tasks

We recruited the following members with a good distribution of experience (1 Obs&Gyn with trial experience, 1 statistician, 1 perinatal epidemiologists)

Zahida Qureshi zahida@qureshi.co.ke; https://profiles.uonbi.ac.ke/qureshi/

Erik Lampa erik.lampa@medsci.uu.se ; https://katalog.uu.se/empinfo/?id=N11-1203

Jennifer Hall jennifer.hall@ucl.ac.uk; https://iris.ucl.ac.uk/research/personal?upi=JAHAL68

Rational for DSMB

ALERT is a stepped-wedge cluster (hospital) randomised trial to evaluate a public health intervention composed of four components of end-user participation, midwifery training, quality improvement and mentoring for the head of maternity (find the summary attached). It is funded by Horizon 2020 and will be implemented in Benin, Malawi, Tanzania and Uganda. The intervention aims to reduce perinatal mortality and improve intrapartum care including user responsiveness.

ALERT is not a clinical trial. ALERT will implement and evaluate the effect of non-clinical interventions such as training and quality improvement. However, also non-clinical intervention through changes in practises may have clinical consequences. For example, changes in clinical practice may increase in Caesarean section rates, those clinically justified but potentially also some without clear indication. This is an unintended consequence and as Caesarean section bear risks for mother and their babies, Caesarean section rate will need to be monitored. Moreover, the main study outcomes include stillbirth and early neonatal mortality rate. Thus, the establishment of a DSMB is indicated.

The DSMB should have the following tasks

- To review the trial procedure including randomization and attainment of outcomes (April 2021)
- To review protocol compliance and enrolment at month 12 of the trial / June 2022 (Interim review of protocol compliance)
- To review the effects of the intervention at month 12 and months 24 of the trial, including a check on futility, safety and interim impact to advise on continuation (June 2022 & June 2023,)
- To advise the PI (Claudia Hanson) and the ALERT Trial Management Team

The ALERT trial management team will include the statistician Nicola Orsini (Karolinska Institutet) Joseph Akuze (Makerere University), Peter Waiswa (PI Uganda, Makerere University), Jean-Paul Dossou (PI Benin, CERHHUD), Effie Chipata (PI Malawi, College of Medicine) and Andrea Pembe (PI Tanzania, MUHAS).

April 2021: The ALERT trial management team will prepare a full trial protocol. We will invite for a meeting either in-person or using teleconferencing to discuss the protocol and establish the further working modalities. During this meeting stopping rules and procedures for severe adverse events will also be discussed as well as the forms for sharing unexpected findings and events.

June 2022: We will prepare a report on protocol compliance and share data together with an analysis do-file to enable the DSMB to review the enrolment and safety.

June 2023: We will prepare an updated report on protocol compliance and share data together with an analysis do-file to enable the DSMB to review the enrolment and safety. The interim and the final analysis will be blinded.

We expect the team to be available for the DSMB at the indicated timepoints and expect at each timepoint about 8 hours to be spend on this assignment including reading and commenting on the report and joining the virtual or actual meetings. Potential travel expenses will be taken care of by the project. We have not foreseen any renumeration.
