# Supplementary material for: A multi-faceted hospital-based intervention for intrapartum care in sub-Saharan Africa: a stepped-wedge cluster-randomized trial
Source: Nat Med. 2026 Aug 26;32(10):3832–41. doi: 10.1038/s41591-026-04599-w (PMC13668680; doi:10.1038/s41591-026-04599-w)
Supplement: Supplementary file 2 [file 41591_2026_4599_MOESM2_ESM.pdf]

nature portfolio

Corresponding author(s): Claudia Hanson

Last updated by author(s): Jul 6, 2026

Reporting Summary

Nature Portfolio wishes to improve the reproducibility of the work that we publish. This form provides structure for consistency and transparency in reporting. For further information on Nature Portfolio policies, see our Editorial Policies and the Editorial Policy Checklist.

Statistics

For all statistical analyses, confirm that the following items are present in the figure legend, table legend, main text, or Methods section.

n/a	Confirmed	
☐	☒	The exact sample size (*n*) for each experimental group/condition, given as a discrete number and unit of measurement
☐	☒	A statement on whether measurements were taken from distinct samples or whether the same sample was measured repeatedly
☐	☒	The statistical test(s) used AND whether they are one- or two-sided<br>*Only common tests should be described solely by name; describe more complex techniques in the Methods section.*
☐	☒	A description of all covariates tested
☐	☒	A description of any assumptions or corrections, such as tests of normality and adjustment for multiple comparisons
☐	☒	A full description of the statistical parameters including central tendency (e.g. means) or other basic estimates (e.g. regression coefficient) AND variation (e.g. standard deviation) or associated estimates of uncertainty (e.g. confidence intervals)
☐	☒	For null hypothesis testing, the test statistic (e.g. *F*, *t*, *r*) with confidence intervals, effect sizes, degrees of freedom and *P* value noted<br>*Give P values as exact values whenever suitable.*
☒	☐	For Bayesian analysis, information on the choice of priors and Markov chain Monte Carlo settings
☐	☒	For hierarchical and complex designs, identification of the appropriate level for tests and full reporting of outcomes
☒	☐	Estimates of effect sizes (e.g. Cohen's *d*, Pearson's *r*), indicating how they were calculated

Our web collection on statistics for biologists contains articles on many of the points above.

Software and code

Policy information about availability of computer code

Data collection	Research Electronic Data Capture REDCap, v13
Data analysis	Stata, version 18, https://zenodo.org/records/20671445

For manuscripts utilizing custom algorithms or software that are central to the research but not yet described in published literature, software must be made available to editors and reviewers. We strongly encourage code deposition in a community repository (e.g. GitHub). See the Nature Portfolio guidelines for submitting code & software for further information.

Data

Policy information about availability of data

All manuscripts must include a data availability statement. This statement should provide the following information, where applicable:

- Accession codes, unique identifiers, or web links for publicly available datasets
- A description of any restrictions on data availability
- For clinical datasets or third party data, please ensure that the statement adheres to our policy

Data from the perinatal e-registry will be made available from 31st of December 2027, which is 3 years following the finalization of the trial, in accordance with the ALERT data management plan and publication policy. This restriction was instituted to allow country researchers from Benin, Malawi, Tanzania and Uganda, particularly early career researchers and PhD students, time to conduct analyses and publish findings. Prior to this date, data can be made available after consideration by the ALERT steering committee, which is led by the corresponding author, Claudia Hanson.

The ALERT data dictionary and supporting documents are available at https://cordis.europa.eu/project/id/847824/results. Researchers from any country with an affiliation to a recognized university or research institution are eligible to apply for access to the ALERT dataset and applicants should submit a detailed project proposal (no more than 5 pages) to the corresponding author at claudia.hanson@ki.se, outlining the project aims, methodology, requested variables, planned statistical analyses, evidence of ethics approval and benefit to local countries. The criteria used to evaluate the requests are (i) scientific merit, (ii) feasibility, (iii) benefit for local countries and (iv) ethical considerations. The ALERT steering committee will respond to all data requests within 1 month and, if approved, the data will be released shortly after approval. Access to data may be restricted if members of the ALERT team are planning or conducting similar analyses.

Research involving human participants, their data, or biological material

Policy information about studies with human participants or human data. See also policy information about sex, gender (identity/presentation), and sexual orientation and race, ethnicity and racism.

Reporting on sex and gender	Our data are from pregnant women.
Reporting on race, ethnicity, or other socially relevant groupings	no data available. All data are from low and middle income countries in sub-Saharan Africa as reported.
Population characteristics	We detailed the setting (Supplementaty table 1) and the characteristics of the study population (table 1)
Recruitment	We included all mother-baby pairs admitted for childbirth in any of the hospitals between 1st July 2021 and 31st December 2023. We excluded mother-baby pairs who were referred to the hospitals after giving birth.
Ethics oversight	Karolinska Institutet, Sweden (Etikprövningsmyndigheten—Dnr 2020–01587). Uganda National Council for Science and Technology (UNCST)— (HS1324ES). Muhimbili University of Health And Allied Sciences (MUHAS) Research and Ethics Committee, Tanzania (MUHAS-REC-04-2020-118) and The Aga Khan University Ethical Review Committee, Tanzania (AKU/2019/044/fb). College of Medicine Research and Ethics Committee (COMREC), Malawi—(COMREC P.04/20/3038). Comité National d'Ethique pour la Recherche en Santé, Cotonou, Bénin—(83/MS/DC/SGM/CNERS/ST). The Institutional Review Board at the Institute of Tropical Medicine Antwerp and The Ethics Committee at the University Hospital Antwerp, Belgium—(ITG 1375/20. B3002020000116).

Note that full information on the approval of the study protocol must also be provided in the manuscript.

Field-specific reporting

Please select the one below that is the best fit for your research. If you are not sure, read the appropriate sections before making your selection.

☒ Life sciences ☐ Behavioural & social sciences ☐ Ecological, evolutionary & environmental sciences

For a reference copy of the document with all sections, see nature.com/documents/nr-reporting-summary-flat.pdf

Life sciences study design

All studies must disclose on these points even when the disclosure is negative.

Sample size	The sample size was determined by the main trial. See https://bmchealthservres.biomedcentral.com/articles/10.1186/s12913-021-07155-z We used all data available.
Data exclusions	We included all trial data. We excluded the data from Feb 2021 to June 2021 of the piloting phase of the perinatal e-registry
Replication	The analysis code is published on https://zenodo.org/records/20671445
Randomization	Randomization using Stata and a pre-prepared code was carried out on 17th June 2020 by an independent statistician after obtaining consent from the participating hospitals (Supplementary statistical plan). Stratification by country was used to enable random allocation of the four hospitals.
Blinding	no blinding. As with all public health interventions neither the team nor those implementing hospital could be blinded as they were the implementers

Reporting for specific materials, systems and methods

We require information from authors about some types of materials, experimental systems and methods used in many studies. Here, indicate whether each material, system or method listed is relevant to your study. If you are not sure if a list item applies to your research, read the appropriate section before selecting a response.

Materials & experimental systems

n/a	Involved in the study
☒	☐ Antibodies
☒	☐ Eukaryotic cell lines
☒	☐ Palaeontology and archaeology
☒	☐ Animals and other organisms
☐	☒ Clinical data
☒	☐ Dual use research of concern
☒	☐ Plants

Methods

n/a	Involved in the study
☒	☐ ChIP-seq
☒	☐ Flow cytometry
☒	☐ MRI-based neuroimaging

Clinical data

Policy information about clinical studies
All manuscripts should comply with the ICMJE guidelines for publication of clinical research and a completed CONSORT checklist must be included with all submissions.

Clinical trial registration: ALERT is registered (June 17, 2020) in the Pan African Clinical Trial Registry at 202006793783148

Study protocol: https://bmchealthservres.biomedcentral.com/articles/10.1186/s12913-021-07155-z

Data collection: This study included singleton births from a prospective observational study in 16 hospitals in Benin, Malawi, Tanzania and Uganda collected as part of the Action Leveraging Evidence to Reduce perinatal morTality and morbidity (ALERT) study.50 Benin, Malawi, Tanzania and Uganda are low- and lower middle-income countries, facing a large perinatal mortality burden.1 The countries are categorised as low or lower-middle income countries with Real Gross Domestic Product per capita of between 1,500 (Malawi) to 3,300 (Benin) US dollar. Uptake to antenatal and childbirth care in facilities is improving but health systems are weak. Childbirth care faces larger out-of-pocket expenses, particular in Benin and Uganda, restricting preventive uptake of hospital care.

In each of the countries, four large to medium-size hospitals were included with more than 2500 births per annum. Hospitals were typically district or regional public and private-for-nonprofit (faith-based) facilities, although the national referral hospital in Cotonou, Benin also took part in the study. In this analysis, we included all mother-baby pairs admitted for childbirth in any of the hospitals between 1st July 2021 and 31th December 2023. We excluded mother-baby pairs who were referred to the hospitals after giving birth.

Outcomes: The trial's primary outcomes were in-facility (fresh) stillbirths defined as the number of fresh stillbirths of at least 1000g per 1000 live- and stillbirth and a composite early perinatal mortality indicator defined as in-facility (fresh) stillbirths and 24-hour neonatal mortality defined as death of a neonate within 24-hour after birth per 1000 live- and stillbirths, both assessed using our perinatal e-registry 32. Secondary outcomes were hypoxic-ischemic events defined as a neonate with Apgar < 7 at 5 min, neonatal seizures, cesarean sections, severe maternal morbidities defined as women with hysterectomy, laparotomy, blood transfusion, admission to intensive care unit or referral to higher level facility, all expressed per 1000 live- and stillbirths using the perinatal e-registry. Our respectful care focused secondary outcomes were responsiveness and respectful treatment (1 minus mistreatment). They were assessed using exit interview with women after having given birth. Process measures were not pre-specified due to the co-design of the intervention leading to adapted training and intervention components in the development phase. We report on fetal heart rate check at admission, fetal monitoring during the 1st stage of labour, detection of fetal distress, resuscitation of babies, companionship and breastfeeding within one hour of birth.

Plants

Seed stocks: *Report on the source of all seed stocks or other plant material used. If applicable, state the seed stock centre and catalogue number. If plant specimens were collected from the field, describe the collection location, date and sampling procedures.*

Novel plant genotypes: *Describe the methods by which all novel plant genotypes were produced. This includes those generated by transgenic approaches, gene editing, chemical/radiation-based mutagenesis and hybridization. For transgenic lines, describe the transformation method, the number of independent lines analyzed and the generation upon which experiments were performed. For gene-edited lines, describe the editor used, the endogenous sequence targeted for editing, the targeting guide RNA sequence (if applicable) and how the editor was applied.*

Authentication: *Describe any authentication procedures for each seed stock used or novel genotype generated. Describe any experiments used to assess the effect of a mutation and, where applicable, how potential secondary effects (e.g. second site T-DNA insertions, mosiacism, off-target gene editing) were examined.*
