# Supplementary material for: A multi-faceted hospital-based intervention for intrapartum care in sub-Saharan Africa: a stepped-wedge cluster-randomized trial
Source: Nat Med. 2026 Aug 26;32(10):3832–41. doi: 10.1038/s41591-026-04599-w (PMC13668680; doi:10.1038/s41591-026-04599-w)
Supplement: Supplementary file 3 [file 41591_2026_4599_MOESM3_ESM.pdf]

nature portfolio

Peer Review File

A multi-faceted hospital-based intervention for intrapartum care in Sub-Saharan Africa: a step-wedge, cluster-randomised trial

Corresponding Author: Professor Claudia Hanson

This file contains all reviewer reports in order by version, followed by all author rebuttals in order by version.

Version 0:

Reviewer comments:

Reviewer #2

(Remarks to the Author)
Summary of the key results
This stepped-wedge cluster-randomized trial evaluated the ALERT intervention four-component package targeting intrapartum care in 16 hospitals across four SSA countries. Among 134,630 women and 139,300 neonates, the intervention was associated with a 22% reduction in the composite outcome of early perinatal mortality (fresh stillbirth plus 24-hour neonatal mortality; adjusted OR 0.78, 95% CI 0.65–0.94) and a 19% reduction in hypoxic-ischemic events (Apgar <7; adjusted OR 0.81, 95% CI 0.69–0.95). CS rates increased by 14% (adjusted OR 1.14, 95% CI 1.01–1.27). No statistically significant effects were observed on responsiveness or respectful care measures. Process indicators showed substantial improvements in fetal heart rate monitoring on admission (OR 25.05) and during labor (OR 2.05).

Originality and significance
The study addresses an important gap in improving routine intrapartum care quality in sub-Saharan African hospital settings. The integration of co-design with implementation strategies represents a novel approach to context-adapted quality improvement. However, the incremental novelty beyond previously published multi-component QI interventions (e.g., Preterm Birth Initiative, Nepal Perinatal Quality Improvement Project) could be more explicitly articulated. The focus on routine rather than exclusively emergency obstetric care is a valuable contribution aligned with EWENE priorities.

Data & methodology:
Strengths:
• Robust stepped-wedge design appropriate for phased implementation
• Innovative e-registry with rigorous quality assurance procedures
• Hospital-specific mixed-effects models appropriately acknowledging heterogeneity (I^2 >50% for most outcomes)
• Comprehensive sensitivity analyses (per-protocol, exclusion of implementation outliers)

Concerns:

There are no specification of what proportion of providers received training, how many QI cycles were completed per hospital, and how consistently mentorship was delivered. Unable to assess whether the intervention was implemented as intended. Please elaborate on this.

1. Numerous secondary outcomes and process indicators are examined without explicit adjustment for multiple testing, increasing Type I error risk. While the consistency of effects across mortality/morbidity outcomes is reassuring, this limitation should be acknowledged.

2. With 16 clusters, the DerSimonian-Laird estimator can underestimate between-study variance. Are there any alternative approaches (e.g., Hartung-Knapp adjustment) that were considered for sensitivity?

3. The sensitivity analysis excluding three hospitals identified post-hoc as having "implementation barriers" (Tanzania Hospitals 3 and 4, Malawi Hospital 3) is concerning. Doesn't this post-hoc exclusion risks bias? though the consistency of results with and without exclusion is reassuring. Please clarify whether these hospitals were identified based on statistical criteria, contextual knowledge, or both.
4. The protocol mentions a 2-week transition period for intervention adoption. Please clarify how observations during this period were handled analytically.
5. The dramatic improvement in "fetal heart check on admission" is presented. Please elaborate on the method of monitoring (intermittent auscultation vs. electronic monitoring) or how these indicators were operationally defined and extracted. With baseline coverage already at 89%, clarify whether the improvement represents a meaningful clinical change or primarily a documentation improvement.
6. The APGAR scoring method has several limitations including; subjectivity and inter-rater variability in busy, understaffed settings, its not really specific for hypoxic insult (low APGAR can result from prematurity, infection, maternal sedation, congenital anomalies) an its unclear whether the 5-minute score represents the only assessment or the final score after resuscitation.

The statement that 96% of low-Apgar neonates had elevated lactate "confirming reasonable sensitivity and specificity" overstates the evidence. Lactate >5.5 mmol/L is itself a surrogate, not a diagnostic gold standard for HIE. Consider reframing this outcome.
7. The study reports "12% lower odds of fresh stillbirth (AOR 0.88, 95% CI 0.68-1.13)". Fresh stillbirth result is not statistically significant, and this should be explicitly noted when first presented to avoid overinterpretation.

Conclusions: Robustness, Validity, Reliability
Conclusions are generally well-supported by the data.

Suggested improvements:

8. Specify criteria for identifying the three excluded hospitals in sensitivity analysis. Present results both including and excluding them transparently.

9. If available, provide data on indications, maternal morbidity, or neonatal outcomes stratified by delivery mode to support the interpretation that increased rates reflect improved care rather than overuse. If unavailable, strengthen the discussion of this limitation.

10. The realist process evaluation is mentioned but not integrated. Preliminary qualitative findings could illuminate why the intervention worked in some hospitals but not others, and why respectful care measures did not improve.

References: appropriate credit to previous work?
Yes. The manuscript appropriately cites key prior work, including:

Clarity and context: lucidity of abstract/summary, appropriateness of abstract, introduction and conclusions
Abstract: Clear.

Introduction: Effectively sets the context (high perinatal mortality in SSA, increased facility births, quality of care gaps) and justifies the need for multicomponent interventions. Well-articulated.

Conclusions: The final sentence appropriately emphasizes the potential of strengthening routine midwifery care.

(Remarks on code availability)

Reviewer #3

(Remarks to the Author)
I commend the authors on the work they presented---- very important to find pragmatic, scalable interventions in this field. The study evaluates a multi faceted, co designed intrapartum care intervention—combining training, quality improvement, and leadership mentoring—implemented in 16 hospitals across Benin, Malawi, Tanzania, and Uganda. It finds that the

intervention reduced early perinatal mortality and hypoxic ischemic events, alongside improvements in key clinical processes such as fetal monitoring.

1. The abstract should briefly describe the key components of the intervention so that readers can understand what constitutes the “multi-faceted” approach employed in this trial.
2. The manuscript states two primary outcomes; the authors should clarify how these were defined a priori and interpret the differing widths and statistical significance of their confidence intervals in relation to trial power, event rates, and potential heterogeneity.
3. The sample size calculation is currently referenced indirectly—this should be included in the main text to allow readers to assess whether the study was adequately powered for both primary outcomes.
4. Additional methodological detail is needed on the construction, scoring, and interpretation of the responsiveness and respectful care indices, including how “high” versus “low” scores were defined and validated.
5. The authors should clarify whether secondary and process outcomes were adjusted in the same manner as the primary outcomes.
6. Given the hypothesized mechanisms of effect, an exploratory analysis examining whether increased fetal heart rate monitoring and/or higher Cesarean section rates mediated the observed reductions in mortality would strengthen causal interpretation.
7. Several estimates have wide or imprecise confidence intervals, and the discussion should reflect the resulting uncertainty and caution when drawing inferences.
8. The null or attenuated effect on fresh stillbirth warrants further explanation; the authors should outline potential hypotheses, including misclassification, reporting variations, or differences in pathophysiology relative to early neonatal mortality.
9. The scalability of this multi-component intervention requires elaboration, particularly whether the co-design element necessitates hospital-specific tailoring or whether a regionally standardized model could be feasible.
10. The discussion references an economic analysis, but results are not presented; including at least a brief summary or directing readers to where these findings will appear would improve completeness.

(Remarks on code availability)

Reviewer #4

(Remarks to the Author)
Methodological review: Authors present a stepped-wedge cluster trial of a complex intervention to reduce perinatal mortality. This is an impressive piece of work and it can be published, but there are some points which should be considered – potentially addressable with discussion.

• The manuscript describes two outcomes (fresh stillbirth and a composite) as primary outcomes. As far as I can see, no strategy has been adopted to control Type 1 error rate given that we have two primary outcomes. Inference has been made at a nominal 5% significance level for both. The discussion mentions “no adjustment for multiple testing despite examining numerous secondary outcomes” but does not mention the impact of having two primary outcomes.
• Deviation from the originally planned analysis has been explained in the text and in the SAP, and makes sense.
• The SAP speaks of two interim analyses with pre-specified stopping rules and statistical criteria (without stating the criteria, unless I missed it). I don’t think this is described in the manuscript – what was done, what rules were applied, and how was the analysis adjusted to account for this?
• The SAP describes graphical presentation of outcomes trends over time in each hospital. I don’t think this has been included, but might have been a useful addition.
• The SAP suggested that certain subgroup analyses but was noncommittal about this – and none appear to have been reported. For the authors to confirm that none were performed.
• If I understand correctly, neonates delivered as multiples were treated as independent observations (no allowance for clustering within mother). Okay, perhaps it would have been difficult to incorporate this in practice, but I suppose this is another factor that may impact type 1 error.
• Evidence suggests that outcomes became worse under intervention in one site (Tanzania 3, and the estimates in this site looks pretty definitive). Did any understanding of this come out of interviews. I’m not sure that it is discussed.
• The language used to describe the fresh stillbirth result, which refers only to the point estimate, is potentially misleading when the CI is compatible with benefit or slight harm “the intervention was associated with 12% lower odds of fresh stillbirth (adjusted odds ratio AOR 0.88, 95% CI 0.68-1.13)”.
As mentioned above, it is an impressive trial in many respects and these are complex studies, so some allowance for messiness has to be made. Some discussion of the points mentioned above and implications for type 1 error control in the study appear to be warranted however.

[redacted]

(Remarks on code availability)

I have eyeballed, but have not rerun the code (I am not really a Stata user).

Version 1:

Reviewer comments:

Reviewer #2

(Remarks to the Author)
A: This stepped-wedge cluster-randomized trial evaluated the ALERT intervention—a multi-faceted package comprising co-design, competency-based training, quality improvement cycles, and leadership mentoring—across 16 hospitals in four sub-Saharan African countries. Among 134,630 women and 139,300 neonates, the intervention was associated with a 22% reduction in early perinatal mortality (fresh stillbirth + 24-hour neonatal mortality; aOR 0.78, 95% CI 0.65–0.94) but no statistically significant reduction in fresh stillbirth alone (aOR 0.88, 95% CI 0.68–1.13). Hypoxic-ischemic events (Apgar <7) decreased by 19% (aOR 0.81, 95% CI 0.69–0.95), while Cesarean section rates increased by 14% (aOR 1.14, 95% CI 1.01–1.27). No significant effects were observed on responsiveness or respectful care measures. Process indicators showed substantial improvements in fetal heart rate monitoring on admission (OR 25.05) and during labor (OR 2.05). The intervention appeared safe, with no adverse events attributed to the trial procedures.

B:The study addresses a critical gap in improving routine intrapartum care in SSA. The integration of co-design as a foundational step—rather than an adjunct—represents a genuine methodological advance.
Novel and significant. The pragmatic design and four-country generalizability offer high potential for scalability if cost-effectiveness holds.

C:
Strengths:
Robust stepped-wedge design appropriate for phased implementation and policy buy-in.
Innovative e-registry with rigorous QA.
Missing data <1% for primary outcomes.
Hospital-specific mixed-effects models appropriately acknowledge substantial heterogeneity (I^2 >50% for most outcomes).
Comprehensive sensitivity analyses consistently support main findings.
All other concerns were addressed in rebuttal.

D: Appropriate use of statistics and treatment of uncertainties. Statistical approach is robust and limitations are transparently discussed.

E: The primary conclusion—that the ALERT intervention reduced early perinatal mortality—is robust.
F: No additional experiments or major data revisions are required. The authors have thoroughly addressed reviewer concerns.

G:The manuscript appropriately cites key prority trials.

H:The study is methodologically sound, the data support the primary conclusion, and the authors have responded comprehensively to concerns

(Remarks on code availability)

Reviewer #3

(Remarks to the Author)
Thank you for addressing my comments. I have nothing further to add.

(Remarks on code availability)

Reviewer #4

(Remarks to the Author)
As noted in the previous review, my view of the trial is largely positive. Authors have addressed most of the points, but appear t have accidentally missed one key point. Beneath the comment from the previous review:
"• The manuscript describes two outcomes (fresh stillbirth and a composite) as primary outcomes. As far as I can see, no strategy has been adopted to control Type 1 error rate given that we have two primary outcomes. Inference has been made at a nominal 5% significance level for both. The discussion mentions "no adjustment for multiple testing despite examining numerous secondary outcomes" but does not mention the impact of having two primary outcomes."
Authors have written:
"Response: We thank the reviewer for the methodological overview. The key analytical choices (mixed-effects specification, multiplicity handling, exclusion criterion, transition-window treatment, interim-analysis stance, and handling of multiple births) are addressed point-by point below. No changes to the pre-specified primary analysis were made after unblinding."

And then do not address the issue of having two primary outcomes without any strategy to control Type 1 error at all. I presume this is a mistake in the preparation of the response document. It needs some comment. Even if the solution is to add a statement to the manuscript saying “there was no control for Type 1 error due to having two primary outcomes” , that’s okay.

• In the response authors have written “Our SAP did not pre-specify interim analyses with formal statistical stopping boundaries. Safety oversight was provided by the independent Data Safety Monitoring Board under its pre-defined terms of reference; no alpha-spending or sequential testing rules were applied and no adjustment for sequential analysis is required. We have clarified the SAP wording to remove ambiguity.”

Here is the text in the SAP to which I referred (5.4 Interim Analyses page 16):

“The ALERT trial will conduct two interim analyses: one when approximately 50% of the total sample size is reached and another when approximately 75% of the total sample size is reached. The interim analyses will be conducted using pre-specified stopping rules and statistical criteria.”
This contradicts the response of the authors, and isn’t particularly ambiguous! If the authors have retroactively revised the SAP to include this…I don’t think that’s the best solution.
If this shouldn’t have been in the SAP, and wasn’t done, then okay! These things happen, reality is messy, life is hard etc. But the key is to be transparent about all of this.

These are the two outstanding points to be addressed – I probably don’t need to see it again, but I think some comment on the se aspects in the manuscript should be required before acceptance.
Congratulations on the impressive work, I’m happy to sign the review for transparency

[redacted]

(Remarks on code availability)
Not reviewed

Open Access This Peer Review File is licensed under a Creative Commons Attribution 4.0 International License, which permits use, sharing, adaptation, distribution and reproduction in any medium or format, as long as you give appropriate credit to the original author(s) and the source, provide a link to the Creative Commons license, and indicate if changes were made.
In cases where reviewers are anonymous, credit should be given to 'Anonymous Referee' and the source.
The images or other third party material in this Peer Review File are included in the article’s Creative Commons license, unless indicated otherwise in a credit line to the material. If material is not included in the article’s Creative Commons license and your intended use is not permitted by statutory regulation or exceeds the permitted use, you will need to obtain permission directly from the copyright holder.

To view a copy of this license, visit https://creativecommons.org/licenses/by/4.0/

Response to referees

Manuscript ID NMED-A149287 titled "Effects of a multi-faceted intervention to improve intrapartum care: A stepped-wedge, cluster-randomized trial in 16 hospitals in Benin, Malawi, Tanzania and Uganda"

A list of the numbered detailed responses is given below with the following format:

Normal: a copy of the review comment

Blue: our response

Italic: copy of changed text in manuscript where appropriate

Reviewers' Comments to Author:

Editorial response

The reviewers find the study is of high interest and importance to women's health and implementation science. However, they have also highlighted some concerns around the statistical analyses and methodological or technical approach which would be important to clarify.

Response: Thank you very much for your positive response.

It is also important that you provide more information on facility recruitment, including the dates in which the first and last facilities were recruited into the trial, as well as formal start and end dates of the trial.

Response: Thank you. We agree that the description of the selection of hospitals was not clear and have added the following:

Hospitals were purposely selected in March 2020 drawing from a national facility list to identify high-caseload hospitals. Criteria included i) minimum caseload of 2,500 births per year; ii) availability of Cesarean section and blood transfusion; iii) preferably located in rural districts; and iv) consisting of a mix of typical public and (faith-based) hospitals and v) interest in collaboration and no other ongoing initiative focusing on intrapartum care.[1] The final selection was done in consultation with the Ministry of Health and took into account feasibility concerns in terms of travel time between the hospitals to ensure operational efficiency.

Editorial points to be addressed. Please note that not all of these might apply to your manuscript, in which case please respond with "not applicable." Structural changes to re-organise the manuscript do not need to be tracked.

* Abstract should be no more than 200 words and for trials should adhere to the CONSORT framework. You must state if the primary outcome was or was not met and provide the effect size and relevant uncertainty estimate. The conclusion of the study must focus only on the primary outcome and safety/tolerability. You must report either all or none of the secondary outcomes, given the space limitation, I would suggest removing all mention of secondary outcomes from the abstract.

Response: Thank you, we have amended this and believe that our abstract meets the requirements.

* Please include the trial registration number at the end of the Abstract.

Response: Thank you, this is provided.

"We developed the facility-based, multi-faceted Action Leveraging Evidence to reduce perinatal Mortality and Morbidity (ALERT) intervention, which included i) co-design, ii) training, iii) quality improvement and iv) leadership mentoring of maternity units' heads to address high perinatal mortality and morbidity in Benin, Malawi, Tanzania and Uganda. ALERT promoted essential practices of intrapartum childbirth and it was evaluated using a stepped-wedge cluster-randomized trial with five six-month periods across 16 hospitals. Primary outcomes were in-facility fresh stillbirths and the composite indicator of early perinatal mortality (fresh stillbirth and 24-hour neonatal mortality). Secondary outcomes were hypoxic-ischemic events (Apgar score <7), Cesarean section rate, responsiveness, and respectful care. We estimated separate hospital-specific mixed-effects regression models for binary outcomes and continuous variables to account for heterogeneity across hospitals and countries. All models included treatments status, time period, and random intercepts for randomization groups. We combined effects across hospitals using random-effects meta-analysis with the DerSimonian-Laird estimator. Among 134,630 women (139,300 neonates), the intervention was associated with 22% lower odds of the composite outcome early perinatal mortality (AOR 0.78, 95% CI 0.65-0.94), but not with fresh stillbirth (adjusted odds ratio AOR 0.88, 95% CI 0.68-1.13). The odds of hypoxic-ischemic events were reduced by 19% (AOR 0.81, 95% CI 0.69-0.95). Cesarean section rates increased (AOR 1.14 95% CI 1.01-1.27). We saw no statistically significant effects on our responsiveness or respectfulness measurements. While our results are derived from only 16 hospitals, the variety of settings support the importance of observed reduction in early perinatal mortality and morbidity implementing the multi-faceted interventions supporting context-specific strategies to build maternity provider capacities. "

(Funded through European Union No 847824, PACTR registration number 202006793783148)

* The Introduction should be written for a broad, non-specialist medical reader and provide sufficient context for the work.

Response: Thank you, we believe we have done so, and we integrated all reviewer comments (see below)

* Please provide details of your cohort in the first paragraph of the Results. This includes number of individuals screened for enrolment, as well as exact dates of first and last patient enrolment. The CONSORT patient disposition diagram and the baseline characteristics must be included as main non-text items (for which there is a strict limit of 6 tables and/or figures).

Response: Thank you, this has been adhered to.

* The Results should be structured as followed:
- Patient disposition
- Primary outcome(s)
- Secondary outcomes
- Safety
- Exploratory outcomes
- Sensitivity analyses
- Post-hoc analyses

Response: Thank you, we reviewed carefully and added a line on safety and marked clearly the post-hoc analysis. Thus we believe this has been adhered to.

Safety

Our adverse event monitoring as part of the process evaluation indicated no safety concerns in regard to the training and quality improvement. The 14% increase in the odds of Cesarean sections was within expected ranges supporting the safety of the intervention.

* Please remove any subheadings from the Discussion.

* Please ensure all results are presented in the Results section, no new data should be introduced in the Discussion.

* You must include explicit paragraphs of study limitations in the Discussion.

* The overarching conclusion of the study must be based only on the primary outcome and safety data.

Response: Thank you very much. We have carefully reviewed and amended and believe we adhere with the requirements.

* The Methods should include a full description of the inclusion and exclusion criteria, as well as study procedures and statistical analyses (including a power calculation).

Response: Thank you. We amended the sample size and power calculation, which is also published in our protocol paper. In particular, we added on stillbirth mortality as this is also important to evaluate the results.

The study was sufficiently powered (75-80%) to detect a 25% reduction in the outcome perinatal mortality with baseline rates between 1.4% to 2.0% and 30% reduction in intrapartum stillbirth assuming a baseline rate of 1% and 95% confidence intervals (95%CI). Details are available in the supplementary file of the protocol paper.[1]

*Please upload the protocol and SAP with the revision materials, so that reviewers and editors have access to them.

Response: Thank you, we have uploaded these.

* You must ensure that contributions from all individuals in the author list are available in the Author Contributions statement.

Response: Thank you. We are embarrassed, but we forgot this. We have now included the following Author Contributions statement:

CH, LB, MMG, EC, PW, HK, JPD, and ABP conceived the study and obtained the funding. The trial management was done by CH, JA, NO, EC, PW, HK, JPD, and ABP. CH, JA, KSA, HMA, EC, PW, HK, JPD, and ABP led and supervised the study, the data collection and the processing of the data. All ALERT team members supported the trial and the data collection into the perinatal e-registry. CH, KSA, SS, MS curated the data. CH, JA and NO conducted the analysis of the data. All authors contributed to the critical interpretation of the data, critically revised the manuscript and approved the final version.

* Please move all funding sources to the Acknowledgements, including a statement on the role of the funder.

Response: Thank you, we have moved all funding sources to the Acknowledgements and included a statement on the role of the funder.

This project was supported by an implementation science grant (European Commission, Horizon 2020, No 847824). Data collection in Uganda was in addition supported by a grant from the Swedish Research Council (2019-01906_VR). The funding bodies had no role in the study design, data collection, data analysis, interpretation of data, manuscript writing or decision to publish. Our deepest gratitude goes to the women, babies and families as well as the maternity care providers, managers and policy makers in the 16 hospitals and four countries involved in the ALERT study. We thank our data safety and monitoring board (Jennifer Hall, Erik Lampa, & Zahida Qureshi) and the European Commission reviewers France Donnay and Jaan Tulviste.

* Please ensure that all potential competing interests are detailed for all authors. For any authors with no competing interests, this must also be stated.

Response: Thank you, we have included the following statement:

Competing interests

The authors declare no competing interests.

* Please see our guidelines for the Data and Code Availability Statements. "Available on request" is not acceptable, you must provide details of any restrictions to data and code availability. https://www.nature.com/nature-portfolio/editorial-policies/reporting-standards#availability-of-data

Response: Thank you, we amended

Data availability

Data from the perinatal e-registry will be made available from 31st of December 2027, which is 3 years following the finalization of the trial, in accordance with the ALERT data management plan and publication policy. Prior to this date, the data is available upon reasonable request to the ALERT steering committee, which is led by the corresponding author, Claudia Hanson. The ALERT steering committee will respond to all data requests within 1 month and, if approved, the data will be released shortly after approval. Access to data may be restricted if members of the ALERT team are planning or conducting similar analyses.

Code availability statement

The code is made available together with this paper.

* The article file must only contain these items in this order:
• Title
• Author List and affiliations
• Abstract
• Introduction
• Results (with Subheadings)
• Discussion
• Acknowledgements
• Author Contributions
• Competing Interests Statement
• References (for main text only)
• Figure legends (for main text only)
• Tables (note: tables should be pasted into Word files as editable tables, not as images)
• Methods
• Data Availability Statement
• Code Availability Statement
• Methods-only References

Response: Thank you, we believe all requirements and declarations are now included.

Response to Reviewer 2

Summary of the key results
This stepped-wedge cluster-randomized trial evaluated the ALERT intervention four-component package targeting intrapartum care in 16 hospitals across four SSA countries. Among 134,630 women and 139,300 neonates, the intervention was associated with a 22% reduction in the composite outcome of early perinatal mortality (fresh stillbirth plus 24-hour neonatal mortality; adjusted OR 0.78, 95% CI 0.65–0.94) and a 19% reduction in hypoxic-ischemic events (Apgar <7; adjusted OR 0.81, 95% CI 0.69–0.95). CS rates increased by 14% (adjusted OR 1.14, 95% CI 1.01–1.27). No statistically significant effects were observed on responsiveness or respectful care measures. Process indicators showed substantial improvements in fetal heart rate monitoring on admission (OR 25.05) and during labor (OR 2.05).

Originality and significance
The study addresses an important gap in improving routine intrapartum care quality in sub-Saharan African hospital settings. The integration of co-design with implementation strategies represents a novel approach to context-adapted quality improvement.

Response: Thank you very much, we appreciate the positive comment.

However, the incremental novelty beyond previously published multi-component QI interventions (e.g., Preterm Birth Initiative, Nepal Perinatal Quality Improvement Project) could be more explicitly articulated. The focus on routine rather than exclusively emergency obstetric care is a valuable contribution aligned with EWENE priorities.

Response: Thank you. We appreciate the comment. We have detailed more explicitly where our work adds beyond what has been published to date.

"For example, the Preterm Birth Initiative focused on an intervention package addressing specifically prematurity related intra- and immediate postnatal care, including administration of corticosteroids, resuscitation, and kangaroo care.[2] The Nepal quality improvement package equally focused on resuscitation as an important component of immediate postpartum care.[3] In contrast, no training program operationalizing the WHO 2018 intrapartum care guideline has been developed, beyond trials assessing distinct tools, such as the Safe Childbirth Checklist[4] or the new WHO partograph.[5] Intrapartum care, however, is complex and demands a multitude of skills and decisions within multi-professional maternity care teams including readiness to apply the packages to address complications. The lack of attention to routine care, however, hinders programming for the Every Women Every Newborn, Everywhere (EWENE) global initiative, midwifery care models as well as more efficient management when complication occurs.[6]

Data & methodology:
Strengths:
• Robust stepped-wedge design appropriate for phased implementation
• Innovative e-registry with rigorous quality assurance procedures
• Hospital-specific mixed-effects models appropriately acknowledging heterogeneity (I^2 >50% for most outcomes)
• Comprehensive sensitivity analyses (per-protocol, exclusion of implementation outliers)

Response: Thank you, we appreciate the recognition of the strength of this paper.

Concerns:

There are no specification of what proportion of providers received training, how many QI cycles were completed per hospital, and how consistently mentorship was delivered. Unable to assess whether the intervention was implemented as intended. Please elaborate on this.

Response: Thank you for this important point. We indeed monitored the intervention strength in the last year and added a paragraph:

Intervention and reach

The intervention was initiated with competency-based in-facility training delivered to each hospital per country in accordance with the trial design and randomization (Supplementary Fig S5). Training was delivered as short, flexible sessions timed around shift changes with reinforcement sessions on subsequent days. A total of 484 maternity providers, QI focal persons and administrative staff received the initial training, exceeding the 414 staff members originally registered as working in the trial hospitals due to staff rotation demanding re-trainings. (36) A detailed fidelity assessment carried out in the final year of the trial showed that the ALERT team conducted 7, 12, 1 and 9 mentoring visits to hospitals in Benin, Malawi, Tanzania and Uganda respectively. With the exception of Tanzania, the number of visits exceeded the six quarterly on-site mentoring visits specified in the protocol. A total of 112, 284, 175 and 83 hours of mentoring were delivered on-site and via WhatsApp in Benin, Malawi, Tanzania and Uganda, respectively, corresponding to an average of 56 hours of mentoring per quarter per hospital.

1. Numerous secondary outcomes and process indicators are examined without explicit adjustment for multiple testing, increasing Type I error risk. While the consistency of effects across mortality/morbidity outcomes is reassuring, this limitation should be acknowledged.

Response: Our Statistical Analysis Plan (Section 5.3) and protocol paper pre-specified only two primary outcomes and stated that secondary endpoints would be interpreted on consistency of direction and magnitude across related outcomes rather than on individual p-values; we did not therefore apply a formal multiplicity correction. The consistent direction across the mortality and morbidity indicators supports a causal rather than chance interpretation. We now acknowledge this as a limitation in the Discussion and have added a cautionary note at the first mention of secondary outcomes in the Results.

2. With 16 clusters, the DerSimonian-Laird estimator can underestimate between-study variance. Are there any alternative approaches (e.g., Hartung-Knapp adjustment) that were considered for sensitivity?

Response: DerSimonian-Laird pooling was pre-specified in the Statistical Analysis Plan (Section 5.2), but we agree that with 16 clusters, it can under-estimate between-study variance. We therefore added the Hartung-Knapp-Sidik-Jonkman (HKSJ) adjustment as a pre-specified sensitivity analysis for both primary outcomes. HKSJ combines a REML estimate of between-hospital variance with a t-distribution on k−1 degrees of freedom and is the recommended default when the number of clusters is small.

Pooled point estimates are unchanged to two decimal places and confidence intervals are only modestly wider: early perinatal mortality AOR 0.79 (95% CI 0.63–0.98), fresh stillbirth 0.88 (0.66–1.18), hypoxic-ischemic events 0.80 (0.63–1.02), Cesarean section 1.14 (0.99–1.30). Inference is unaffected.

It is also worth noting that the forest plot is a summary display — the primary analysis is the hospital-specific mixed-effects model, which explicitly retains between-hospital heterogeneity ($I^2 > 50\%$ for most outcomes). The HKSJ pool is now shown in Supplementary Table S7, described in Supplementary Methods, and cited with a pointer sentence in the main-text Methods and Results.

We included:

Three pre-specified sensitivity analyses requested during peer review (Hartung-Knapp-Sidik-Jonkman (HKSJ) pooling, per-protocol restricted to hospital-periods with documented full implementation, and country-stratified random-effects meta-analysis) gave results consistent with the primary analysis. For the primary outcome of early perinatal mortality, there was no evidence of between-country heterogeneity of effect ($Q^b(3) = 1.61$, $p = 0.66$). Full descriptions and outputs are provided in Supplementary Methods and Supplementary Tables S7–S9.

The primary intention-to-treat analysis retained all 16 hospitals. The three-hospital exclusion was post-hoc, triggered by pre-specified data-quality monitoring indicators combined with structured contextual review with country teams in December 2024 (criteria in Table S4).

3. The sensitivity analysis excluding three hospitals identified post-hoc as having "implementation barriers" (Tanzania Hospitals 3 and 4, Malawi Hospital 3) is concerning. Doesn't this post-hoc exclusion risks bias? though the consistency of results with and without exclusion is reassuring. Please clarify whether these hospitals were identified based on statistical criteria, contextual knowledge, or both.

Response: Thank you for raising this important point. To clarify, the three hospitals were not excluded from the primary intention-to-treat analysis, which retained all 16 hospitals. Exclusion applied in a post-hoc sensitivity analysis was triggered by pre-specified data-quality monitoring indicators combined with structured contextual review with country teams in December 2024. Criteria are detailed in Table S4. We present the main analysis (Figure 3) and the exclusion sensitivity (Fig S2) side-by-side; inferences are unchanged. We have made explicit in the Methods that the exclusion was post-hoc and informed after outcome data were available.

4. The protocol mentions a 2-week transition period for intervention adoption. Please clarify how observations during this period were handled analytically.

Response: Observations during the 2-week transition window were retained in the intervention arm in the primary analysis, consistent with the permanent hospital-level transition to intervention status specified in the protocol and SAP. A pre-specified per-protocol sensitivity analysis used a more restrictive definition of the intervention period, excluding the transition window to require full implementation fidelity; results were consistent with the primary analysis (Fig S3).

5. The dramatic improvement in "fetal heart check on admission" is presented. Please elaborate on the method of monitoring (intermittent auscultation vs. electronic monitoring) or how these indicators were operationally defined and extracted. With baseline coverage already at 89%, clarify whether the improvement represents a meaningful clinical change or primarily a documentation improvement.

Response: Thank you. We assume that our training on adhering to admission standards including fetal monitoring, and the quality improvement work preparing checklists and trolleys with all needed materials did indeed improve the monitoring and not only the documentation. Indeed, using an odds ratio if proportions are high can suggest a dramatic increase to the

reader, when it is in fact a consolidating improvement. Monitoring is typically done with the Pinard stethoscope. We added this.

6. The APGAR scoring method has several limitations including; subjectivity and inter-rater variability in busy, understaffed settings, its not really specific for hypoxic insult (low APGAR can result from prematurity, infection, maternal sedation, congenital anomalies) an its unclear whether the 5-minute score represents the only assessment or the final score after resuscitation.
The statement that 96% of low-Apgar neonates had elevated lactate "confirming reasonable sensitivity and specificity" overstates the evidence. Lactate >5.5 mmol/L is itself a surrogate, not a diagnostic gold standard for HIE. Consider reframing this outcome.

Response: We appreciate that the Apgar score is a clinical measurement and not a clinical diagnosis. Still, it is listed in major works on perinatal outcomes, including the ICD-11, as a key outcome. We are indeed aware that this is not equal to HIE, and we also do not claim this. We were surprised how well the Apgar score and lactate correlated in the paper we are citing, and also that infection did not change this correlation in earlier work, also cited. But by no means do we want to say that this is an indication of HIE, which would need clinical examination beyond the first 5 minutes. We agree that the 5-min Apgar score includes those with and without resuscitation, but the 1-minute Apgar score is not widely conducted in this setting. Without saying that Apgar score or high lactate have strong predictive values, we still believe it should be used as a measure at birth.

We amended the manuscript:

We were not able to implement lactate testing as a measure independent from clinical judgment to indicate hypoxic-ischemic events for operational and financial reasons. However, we included lactate testing using the Nova lactate point-of-care metre for 437 cases of Apgar <7 cases in two hospitals in Uganda. A total of 420 of 437 (96%) neonates born with an Apgar <7 had a lactate level above 5.5 mmol/l confirming earlier work indicating reasonable sensitivity and specificity of the Apgar score in this setting.[8,9]

7. The study reports "12% lower odds of fresh stillbirth (AOR 0.88, 95% CI 0.68-1.13)". Fresh stillbirth result is not statistically significant, and this should be explicitly noted when first presented to avoid overinterpretation.

Response: Thank you. We agree, this may lead to a wrong impression. We changed the text throughout.

Among 134,630 women (139,300 neonates), the intervention was associated with 22% lower odds of the composite outcome early perinatal mortality (AOR 0.78, 95% CI 0.65-0.94) but not with fresh stillbirth (adjusted odds ratio AOR 0.88, 95% CI 0.68-1.13).

Fresh stillbirths showed a non-significant effect (adjusted OR 0.88; 95% CI 0.68-1.13).

Conclusions: Robustness, Validity, Reliability
Conclusions are generally well-supported by the data.

Suggested improvements:

8. Specify criteria for identifying the three excluded hospitals in sensitivity analysis. Present results both including and excluding them transparently.

Response: The three hospitals (Tanzania Hospitals 3 and 4, Malawi Hospital 3) were identified *a priori* at country-team review meetings in December 2024, before the main analytical code was finalized, using three pre-specified contextual criteria: (i) documented absence of core intervention components — for example, no functional fetal monitoring equipment at Malawi Hospital 3; (ii) implementation barriers recorded in site-level process-evaluation notes — low staff engagement and data-fidelity concerns at Tanzania Hospital 3 following an external audit, and very low case volume with substantial missing outcome data at Tanzania Hospital 4; and (iii) hospital-period cells where the intervention package was not delivered in full, captured by the trial's per protocol indicator. These are listed in Supplementary Table S4.

Following the reviewer's request, we now present both analyses side-by-side in the new Supplementary Table S8. The per-protocol results are broadly consistent with the main analysis: early perinatal mortality attenuates to AOR 0.96 (95% CI 0.76–1.21), fresh stillbirth is unchanged at 0.89 (0.66–1.18), and between-hospital heterogeneity falls markedly — I^2 drops from 58% to 34% for early perinatal mortality, 70% to 40% for fresh stillbirth, and 88% to 64% for Cesarean section. Rationale and full output are in the new Supplementary Methods and Table S8.

9. If available, provide data on indications, maternal morbidity, or neonatal outcomes stratified by delivery mode to support the interpretation that increased rates reflect improved care rather than overuse. If unavailable, strengthen the discussion of this limitation.

Response: Thank you for this comment. A delivery-mode stratified analysis was not pre-specified, and individual-level indications for Cesarean section were not systematically captured in the e-registry — a limitation we now state explicitly in the Discussion. That said, three lines of evidence support the interpretation that the 14% increase in Cesarean odds reflects appropriate rather than over-use:

(i) Maternal safety. Severe maternal morbidity did not rise alongside the increase of Cesarean sections (adjusted OR 0.83, 95% CI 0.61–1.13) — the opposite of what would be expected under over-use.

(ii) Concurrent perinatal benefit. The reduction in early perinatal mortality co-occurred with the Cesarean section increase; under an over-use interpretation, one would not expect a concurrent mortality decline.

(iii) Country-level pattern. A country-stratified random-effects pool (new Supplementary Table S9) shows that the Cesarean section increase is driven principally by Tanzania (AOR 1.48, 95% CI 1.29–1.69), with essentially null effects in Benin (1.13, 0.86–1.49), Malawi (0.95, 0.78–1.16) and Uganda (1.07, 0.87–1.32); the between-country Q^b test is significant ($Q^b(3) = 15.87$, $p = 0.001$). Importantly, Tanzania also showed concurrent country-level reductions in early perinatal mortality, hypoxic-ischemic events and severe maternal morbidity — a pattern inconsistent with a purely documentation-driven rise.

(iv) External validation. A companion analysis of Cesarean deliveries for stillborn babies across the four ALERT countries found consistent clinical indication (Alsina et al., Bull World Health Organ 2025).

We have strengthened the Discussion accordingly, noted the absence of individual-level indication data as a limitation, and cite Supplementary Table S9 for the country-stratified output.

10. The realist process evaluation is mentioned but not integrated. Preliminary qualitative

findings could illuminate why the intervention worked in some hospitals but not others, and why respectful care measures did not improve.

Response: We did not report on the process evaluation as this merits a full paper on its own, which is in progress. However, we added some high-level findings, which are already published on the EU CORDIS homepage.

"The process evaluation confirmed the synergistic effects across the four intervention components. Co-design was reported as having an empowering effect, to enhance the relevance of the training, to reveal previously untapped resources and to improve coordination among the different actors involved in hospital QI activities. Competency-based training was considered valuable both for refreshing clinical skills and for prompting reflection on team interaction and communication. Quality-improvement and leadership mentoring supported the identification of local solutions to a broader range of barriers to implementing evidence-based interventions.[61] Taken together, these findings suggest that the intervention has potential for scalability. It was perceived as relevant and contextually aligned, demonstrated effectiveness and incurred limited costs.[62] Our approach to use the co-design only in selected facilities and provide a mentoring follow-up using WhatsApp further increased feasibility and cost-effectiveness. "

References: appropriate credit to previous work?
Yes. The manuscript appropriately cites key prior work, including:

Clarity and context: lucidity of abstract/summary, appropriateness of abstract, introduction and conclusions
Abstract: Clear.

Introduction: Effectively sets the context (high perinatal mortality in SSA, increased facility births, quality of care gaps) and justifies the need for multicomponent interventions. Well-articulated.

Conclusions: The final sentence appropriately emphasizes the potential of strengthening routine midwifery care.

Response: Thank you, we appreciate this positive assessment and hope we addressed the points appropriately.

Response to Reviewer 3

I commend the authors on the work they presented---- very important to find pragmatic, scalable interventions in this field.
The study evaluates a multi faceted, co designed intrapartum care intervention—combining training, quality improvement, and leadership mentoring—implemented in 16 hospitals across Benin, Malawi, Tanzania, and Uganda. It finds that the intervention reduced early perinatal mortality and hypoxic ischemic events, alongside improvements in key clinical processes such as fetal monitoring.

1. The abstract should briefly describe the key components of the intervention so that readers can understand what constitutes the "multi-faceted" approach employed in this trial.

Response: Thank you. We agree that the components were easily missed and have amended:

We developed the facility-based, multi-faceted Action Leveraging Evidence to reduce perinatal Mortality and Morbidity (ALERT) intervention including i) co-design, ii) training, iii) quality improvement and iv) leadership mentoring of maternity units' heads addressing high perinatal mortality and morbidity in Benin, Malawi, Tanzania and Uganda.

2. The manuscript states two primary outcomes; the authors should clarify how these were defined a priori and interpret the differing widths and statistical significance of their confidence intervals in relation to trial power, event rates, and potential heterogeneity.

Response: Thank you, we used standard measures of assessing intrapartum care.

Both primary outcomes were pre-specified as co-primary in the published protocol (Akuze et al. 2021) and SAP, with no hierarchical testing order. The trial was powered (75–80%) to detect a 25% relative reduction in the composite early perinatal mortality (baseline 1.4–2.0%) and a 30% relative reduction in fresh stillbirth (baseline ≈ 1%). The wider confidence interval for fresh stillbirth reflects its lower event rate and lower a priori power; the composite was pre-specified as the principal efficacy endpoint to stabilize estimation for rare events. Methods and Discussion have been amended.

"Our study was not powered to detect a 20%, but only a 30% reduction of the fresh stillbirth rate which led to imprecise estimates."

3. The sample size calculation is currently referenced indirectly—this should be included in the main text to allow readers to assess whether the study was adequately powered for both primary outcomes.

Response: Thank you, we have added on the sample size in the method section.

"The trial's primary outcomes were in-facility (fresh) stillbirths and a composite early perinatal mortality indicator defined as in-facility (fresh) stillbirths and 24-hour neonatal mortality.[1] Secondary outcomes and process indicators are listed in Table S6. We used a sample size formula for stepped-wedge trials.[1,10] The study was sufficiently powered (75-80%) to detect a 25% reduction in the outcome perinatal mortality with baseline rates between 1.4% to 2.0% and 30% reduction in intrapartum stillbirth assuming a baseline rate of 1% and 95% confidence intervals (95%CI). Details are available in the supplementary file of the protocol.[1] "

4. Additional methodological detail is needed on the construction, scoring, and interpretation of the responsiveness and respectful care indices, including how "high" versus "low" scores were defined and validated.

Response: Thank you. To assess experience of care, we conducted exit interviews using a questionnaire adapted from established responsiveness and mistreatment questionnaires.[11,12]

We added:

We performed an exploratory and confirmatory factor analysis, which identified six factors: "Communication & supportive care", "Hospital environment", "Maintained respect & dignity", "Social support", "Maintained privacy & confidentiality" and "Lack of physical & verbal abuse". Findings were summarized in two summative scores to construct a responsiveness and respectful care score.[13]

5. The authors should clarify whether secondary and process outcomes were adjusted in the same manner as the primary outcomes.

Response: Thank you, indeed, this was not clear. Yes, we used the same methods. Binary secondary and process indicators were analysed with the identical logistic mixed-effects

framework as the primary outcomes: random intercept for randomization group (hospital cluster), fixed effects for intervention status and for calendar time period (categorical). Continuous responsiveness and respectful treatment scores used linear mixed-effects models with the same covariate structure. Hospital-specific estimates were pooled using DerSimonian-Laird random-effects meta-analysis. We clarified:

For binary outcomes (primary and composite outcome) as well as the binary secondary outcomes, we used logistic mixed-effects models with random intercepts for intervention periods and presented odds ratios (95% CI).

6. Given the hypothesized mechanisms of effect, an exploratory analysis examining whether increased fetal heart rate monitoring and/or higher Cesarean section rates mediated the observed reductions in mortality would strengthen causal interpretation.

Response: A formal causal mediation analysis was not pre-specified and we caution against post-hoc mediation in a cluster-randomized design with aggregated hospital-level exposures. Descriptive evidence is consistent with the hypothesised pathway: fetal heart monitoring on admission improved substantially (OR 25.05; 95% CI 9.20–68.23, reflecting near-complete coverage from an already high baseline), the Cesarean section rate rose 14% without a rise in severe maternal morbidity, and hospital-level temporal trends in process and primary outcomes co-moved (Fig S4). A formal mediation analysis requires individual-level, time-stamped linkage between exposures and outcomes and will be explored in the companion realist process evaluation and economic evaluation papers. An interpretive caveat has been added to the Discussion.

7. Several estimates have wide or imprecise confidence intervals, and the discussion should reflect the resulting uncertainty and caution when drawing inferences.

8. The null or attenuated effect on fresh stillbirth warrants further explanation; the authors should outline potential hypotheses, including misclassification, reporting variations, or differences in pathophysiology relative to early neonatal mortality.

Response: Thank you, we appreciate this comment and added:

Our study was not powered to find a 20%, but only a 30% reduction of the fresh stillbirth rate which led to imprecise estimates. Similarly, other estimates had large confidence intervals pointing to the fact that the trial had limited power as adverse perinatal events are fortunately rare.

9. The scalability of this multi-component intervention requires elaboration, particularly whether the co-design element necessitates hospital-specific tailoring or whether a regionally standardized model could be feasible.

10. The discussion references an economic analysis, but results are not presented; including at least a brief summary or directing readers to where these findings will appear would improve completeness.

Response: Thank you, we added a paragraph summarizing results from the economic and realist process evaluation to support the potential scalability. We cite here preliminary results published on the EU Cordis platform while we are preparing full papers as well.

Our multi-centered and multi-country stepped-wedge, cluster-randomized trial suggests that the multi-faceted ALERT intervention reduced perinatal mortality and morbidity. The effects were strongest for early perinatal mortality, but consistent across the indicators of morbidity and mortality. Our economic analysis estimated incremental costs per perinatal death averted of US$805, US$2,651, US$3,188 and US$760 in Benin, Malawi, Tanzania and Uganda,

respectively based on post-implementation perinatal mortality rates with travel costs representing the largest cost component.[41]

The process evaluation confirmed the synergistic effects across the four intervention components. Co-design was reported as having an empowering effect, to enhance the relevance of the training, to reveal previously untapped resources and to improve coordination among the different actors involved in hospital QI activities. Competency-based training was considered valuable both for refreshing clinical skills and for prompting reflection on team interaction and communication. Quality-improvement and leadership mentoring supported the identification of local solutions to a broader range of barriers to implementing evidence-based interventions.[61] Take together, these findings suggest that the intervention has potential for scalability. It was perceived as relevant and contextually aligned, demonstrated effectiveness and incurred limited costs.[62] Our approach to use the co-design only in selected facilities and provide a mentoring follow-up using WhatsApp further increased feasibility and cost-effectiveness.

In summary, the findings highlight the potential of strengthening routine intrapartum care, thus preventive and promotive midwifery care. Our model, including co-design and strengthening midwifery competences and leadership may serve as a model to complement the ongoing work large-scale work on neonatal care within EWENE. Our trial confirms the relevance of combining interventions with a synergistic effect.

Response to Reviewer 4

Methodological review: Authors present a stepped-wedge cluster trial of a complex intervention to reduce perinatal mortality. This is an impressive piece of work and it can be published, but there are some points which should be considered – potentially addressable with discussion.

• The manuscript describes two outcomes (fresh stillbirth and a composite) as primary outcomes. As far as I can see, no strategy has been adopted to control Type 1 error rate given that we have two primary outcomes. Inference has been made at a nominal 5% significance level for both. The discussion mentions “no adjustment for multiple testing despite examining numerous secondary outcomes” but does not mention the impact of having two primary outcomes.

Response: We thank the reviewer for the methodological overview. The key analytical choices (mixed-effects specification, multiplicity handling, exclusion criterion, transition-window treatment, interim-analysis stance, and handling of multiple births) are addressed point-by-point below. No changes to the pre-specified primary analysis were made after unblinding.

• Deviation from the originally planned analysis has been explained in the text and in the SAP, and makes sense.

• The SAP speaks of two interim analyses with pre-specified stopping rules and statistical criteria (without stating the criteria, unless I missed it). I don’t think this is described in the manuscript – what was done, what rules were applied, and how was the analysis adjusted to account for this?

Response: Our SAP did not pre-specify interim analyses with formal statistical stopping boundaries. Safety oversight was provided by the independent Data Safety Monitoring Board under its pre-defined terms of reference; no alpha-spending or sequential testing rules were

applied and no adjustment for sequential analysis is required. We have clarified the SAP wording to remove ambiguity.

• The SAP describes graphical presentation of outcomes trends over time in each hospital. I don't think this has been included, but might have been a useful addition.

Response: Thank you, this information is included as supplementary material.

• The SAP suggested that certain subgroup analyses but was noncommittal about this – and none appear to have been reported. For the authors to confirm that none were performed.

Response: We confirm that no confirmatory subgroup analyses were performed. The hospital-specific forest plots (Figure 3, Fig S2) serve as the most informative display of between-setting variation, and country-level patterns are summarized descriptively in the Results.

• If I understand correctly, neonates delivered as multiples were treated as independent observations (no allowance for clustering within mother). Okay, perhaps it would have been difficult to incorporate this in practice, but I suppose this is another factor that may impact type 1 error.

Response: Thank you for this thoughtful methodological point. Among the 134,630 women in the trial, 5,409 delivered multiples (4.4% in the comparison arm and 3.7% in the intervention arm; 4.0% overall), yielding 139,300 neonates (Table 1). We therefore treated each neonate as an independent observation at the outcome-level analysis. We share the reviewer's intuition that intra-mother clustering between multiples could inflate Type I error, but in the specific setting of this trial, the expected magnitude of that inflation is small for three reasons. (i) The proportion of babies in a multiple-birth pair is 4%, so only this small fraction of the outcome vector carries any intra-mother correlation. (ii) The randomization unit is the hospital, not the mother; the dominant source of non-independence is therefore between-hospital clustering, which is already captured both by the hospital-level random intercept in the mixed-effects logistic model and by the random-effects meta-analysis across the 16 hospital-specific effects. (iii) The peer-reviewed literature on twin clustering in obstetric trials (for example Yelland et al., Archives of Disease in Childhood - Fetal & Neonatal Edition 2025;110) suggests that the additional variance inflation from ignoring within-mother clustering is typically < 5% when the twin prevalence is below 5%. Given these three reasons, and in view of the very consistent direction of effect shown by the three pre-specified sensitivity analyses reported in new Supplementary Tables S7-S9, we have retained the neonate-level analysis in the main text. We have added a sentence to the Limitations paragraph of the main manuscript acknowledging that within-mother clustering among multiples was not explicitly modelled, and a brief pointer in the Methods directing the reader to the new Supplementary Methods and Supplementary Tables S7-S9.

• Evidence suggests that outcomes became worse under intervention in one site (Tanzania 3, and the estimates in this site looks pretty definitive). Did any understanding of this come out of interviews. I'm not sure that it is discussed.

Response: Thank you. We expanded a bit more on the exclusion in the main text. Indeed, these were reflections by the team during the implementation and then also when we checked the main results. We expanded as follows:

For example, hospital 3 in Malawi was a community hospital with severe resource constraints. Hospital 3 in Tanzania was part of another project where teams had to report during birth on process of labor which we believe led to underreporting during the pre-intervention period and limited engagement into the trial because of fear of blame and disciplinary consequences. Details are found in the annex (Tab S4). The analysis excluding these hospitals indicated results consistent with main analysis (Fig S3).

• The language used to describe the fresh stillbirth result, which refers only to the point estimate, is potentially misleading when the CI is compatible with benefit or slight harm "the intervention was associated with 12% lower odds of fresh stillbirth (adjusted odds ratio AOR 0.88, 95% CI 0.68-1.13)".

Response: Thank you, we have changed as also reviewer 2 (point 7) advised and we are also happy to turn down the misleading tone. However, we believe that results are clearly consistent from a clinical standpoint.

As mentioned above, it is an impressive trial in many respects and these are complex studies, so some allowance for messiness has to be made. Some discussion of the points mentioned above and implications for type 1 error control in the study appear to be warranted however.

References

1. Akuze J, Annerstedt KS, Benova L, et al. Action leveraging evidence to reduce perinatal mortality and morbidity (ALERT): study protocol for a stepped-wedge cluster-randomised trial in Benin, Malawi, Tanzania and Uganda. *BMC Health Serv Res* 2021; **21**(1): 1324.
2. Walker D, Otieno P, Butrick E, et al. Effect of a quality improvement package for intrapartum and immediate newborn care on fresh stillbirth and neonatal mortality among preterm and low-birthweight babies in Kenya and Uganda: a cluster-randomised facility-based trial. *Lancet Glob Health* 2020; **8**(8): e1061-e70.
3. Kc A, Ewald U, Basnet O, et al. Effect of a scaled-up neonatal resuscitation quality improvement package on intrapartum-related mortality in Nepal: A stepped-wedge cluster randomized controlled trial. *PLoS Med* 2019; **16**(9): e1002900.
4. Semrau KEA, Hirschhorn LR, Marx Delaney M, et al. Outcomes of a Coaching-Based WHO Safe Childbirth Checklist Program in India. *New England Journal of Medicine* 2017; **377**(24): 2313-24.
5. Vogel JP, Pujar Y, Vernekar SS, et al. Effects of the WHO Labour Care Guide on cesarean section in India: a pragmatic, stepped-wedge, cluster-randomized pilot trial. *Nature Medicine* 2024; **30**(2): 463-9.
6. World Health Organization, UNICEF, UNFPA. Every Women, Every Newborn, Everywhere. 2025. https://ewene.org/ (accessed 8 Feb 2026).
7. Moller AB, Welsh J, Agossou C, et al. Midwifery care providers' childbirth and immediate newborn care competencies: A cross-sectional study in Benin, Malawi, Tanzania and Uganda. *PLOS Glob Public Health* 2023; **3**(6): e0001399.
8. Wanduru P, Straneo M, Sadoo S, et al. Prevalence and risk of adverse intrapartum-related outcomes in Uganda: a cross-sectional study with nested case-control. *BMJ Open* 2025; **15**(10): e099256.

9. Ayebare E, Tumwine JK, Nankunda J, et al. Evaluating predictive values of umbilical cord arterial lactate for adverse newborn outcomes among term-births in northern Uganda: A cross sectional analytical study. *Int J Gynaecol Obstet* 2025; **169**(1): 408-20.
10. Hemming K, Girling A. A menu-driven facility for power and detectable-difference calculation in stepped-wedge cluster-randomized trials. 2014; **The Stata Journal**(14): 2.
11. Afulani PA, Diamond-Smith N, Golub G, Sudhinaraset M. Development of a tool to measure person-centered maternity care in developing settings: validation in a rural and urban Kenyan population. *Reproductive Health* 2017; **14**(1): 118.
12. Bohren MA, Mehrtash H, Fawole B, et al. How women are treated during facility-based childbirth in four countries: a cross-sectional study with labour observations and community-based surveys. *The Lancet* 2019; **394**(10210): 1750-63.
13. Morris Z, Halabi SE, Hanson C, et al. Measuring responsiveness and respectful treatment in maternity care in sub-Saharan Africa: a questionnaire validation and development of a score. *BMC Pregnancy Childbirth* 2025; **25**(1): 329.
14. Snijders R. Final Economic evaluation report. Belgium: European Commission, 2024.
15. Castellano Pleguezuelo V, Kendeya B, Akpi E, et al. Final synthesis report of the realist process evaluation. CORDIS: European Commission, 2024.
16. Zamboni K, Schellenberg J, Hanson C, Betran AP, Dumont A. Assessing scalability of an intervention: why, how and who? *Health Policy Plan* 2019; **34**(7): 544-52.

Response to referees

Manuscript ID NMED-A149287 titled "Effects of a multi-faceted intervention to improve intrapartum care: A stepped-wedge, cluster-randomized trial in 16 hospitals in Benin, Malawi, Tanzania and Uganda"

A list of the numbered detailed responses is given below with the following format:

Normal: a copy of the review comment

Blue: our response

Italic: copy of changed text in manuscript where appropriate

Response to Reviewer 2

A: This stepped-wedge cluster-randomized trial evaluated the ALERT intervention—a multi-faceted package comprising co-design, competency-based training, quality improvement cycles, and leadership mentoring—across 16 hospitals in four sub-Saharan African countries. Among 134,630 women and 139,300 neonates, the intervention was associated with a 22% reduction in early perinatal mortality (fresh stillbirth + 24-hour neonatal mortality; aOR 0.78, 95% CI 0.65–0.94) but no statistically significant reduction in fresh stillbirth alone (aOR 0.88, 95% CI 0.68–1.13). Hypoxic-ischemic events (Apgar <7) decreased by 19% (aOR 0.81, 95% CI 0.69–0.95), while Cesarean section rates increased by 14% (aOR 1.14, 95% CI 1.01–1.27). No significant effects were observed on responsiveness or respectful care measures. Process indicators showed substantial improvements in fetal heart rate monitoring on admission (OR 25.05) and during labor (OR 2.05). The intervention appeared safe, with no adverse events attributed to the trial procedures.

B:The study addresses a critical gap in improving routine intrapartum care in SSA. The integration of co-design as a foundational step—rather than an adjunct—represents a genuine methodological advance.
Novel and significant. The pragmatic design and four-country generalizability offer high potential for scalability if cost-effectiveness holds.

C:
Strengths:
Robust stepped-wedge design appropriate for phased implementation and policy buy-in.
Innovative e-registry with rigorous QA.
Missing data <1% for primary outcomes.
Hospital-specific mixed-effects models appropriately acknowledge substantial heterogeneity (I^2 >50% for most outcomes).
Comprehensive sensitivity analyses consistently support main findings.
All other concerns were addressed in rebuttal.

D: Appropriate use of statistics and treatment of uncertainties. Statistical approach is robust and limitations are transparently discussed.

E: The primary conclusion—that the ALERT intervention reduced early perinatal mortality—is robust.
F: No additional experiments or major data revisions are required. The authors have

thoroughly addressed reviewer concerns.

G:The manuscript appropriately cites key prority trials.

H:The study is methodologically sound, the data support the primary conclusion, and the authors have responded comprehensively to concerns.

Response: Thank you for your kind comments.

Response to Reviewer 3

Thank you for addressing my comments. I have nothing further to add.

Response: Thank you for your kind comments.

Response to Reviewer 4

As noted in the previous review, my view of the trial is largely positive. Authors have addressed most of the points, but appear to have accidentally missed one key point. Beneath the comment from the previous review:
"• The manuscript describes two outcomes (fresh stillbirth and a composite) as primary outcomes. As far as I can see, no strategy has been adopted to control Type 1 error rate given that we have two primary outcomes. Inference has been made at a nominal 5% significance level for both. The discussion mentions "no adjustment for multiple testing despite examining numerous secondary outcomes" but does not mention the impact of having two primary outcomes."
Authors have written:
"Response: We thank the reviewer for the methodological overview. The key analytical choices (mixed-effects specification, multiplicity handling, exclusion criterion, transition-window treatment, interim-analysis stance, and handling of multiple births) are addressed point-by point below. No changes to the pre-specified primary analysis were made after unblinding."
And then do not address the issue of having two primary outcomes without any strategy to control Type 1 error at all. I presume this is a mistake in the preparation of the response document. It needs some comment. Even if the solution is to add a statement to the manuscript saying "there was no control for Type 1 error due to having two primary outcomes" , that's okay.

Response: We thank the reviewer for highlighting this point and apologize for not addressing it explicitly in our previous response.
The trial pre-specified two primary outcomes: fresh stillbirth and the composite perinatal mortality outcome. No formal multiplicity adjustment was planned or applied. This approach was specified before unblinding and reflected the clinical importance of evaluating both outcomes separately.
We note that the two primary outcomes are closely related, with fresh stillbirth contributing directly to the composite outcome. Consequently, the corresponding statistical tests are positively correlated rather than independent, and any inflation of the overall Type I error rate is expected to be smaller than would occur with two unrelated endpoints. To improve transparency, we have added a statement to the manuscript clarifying that no multiplicity adjustment was pre-specified or performed for the two primary outcomes and that the reported confidence intervals and p-values should be interpreted in that context.

Statistical analysis section:

Two primary outcomes were pre-specified. No formal adjustment for multiplicity was planned or applied because both outcomes were considered clinically important and the analyses followed the pre-specified statistical analysis plan. The two outcomes are closely related, with fresh stillbirth forming part of the composite perinatal mortality outcome.

In the response authors have written "Our SAP did not pre-specify interim analyses with formal statistical stopping boundaries. Safety oversight was provided by the independent Data Safety Monitoring Board under its pre-defined terms of reference; no alpha-spending or sequential testing rules were applied and no adjustment for sequential analysis is required. We have clarified the SAP wording to remove ambiguity."

Here is the text in the SAP to which I referred (5.4 Interim Analyses page 16):

"The ALERT trial will conduct two interim analyses: one when approximately 50% of the total sample size is reached and another when approximately 75% of the total sample size is reached. The interim analyses will be conducted using pre-specified stopping rules and statistical criteria."
This contradicts the response of the authors, and isn't particularly ambiguous! If the authors have retroactively revised the SAP to include this…I don't think that's the best solution.
If this shouldn't have been in the SAP, and wasn't done, then okay! These things happen, reality is messy, life is hard etc. But the key is to be transparent about all of this.

These are the two outstanding points to be addressed – I probably don't need to see it again, but I think some comment on the se aspects in the manuscript should be required before acceptance.
Congratulations on the impressive work, I'm happy to sign the review for transparency

Response: We thank the reviewer for highlighting this inconsistency. The wording in the first SAP stated that two interim analyses would be conducted using pre-specified stopping rules and statistical criteria. In practice, although trial progress and safety data were reviewed by the independent Data Safety Monitoring Board, no formal interim efficacy analyses using statistical stopping boundaries, alpha-spending functions, or sequential testing procedures were undertaken, and no trial decisions were based on such criteria. This is because of the nature of the intervention and the stepped-wedge design.
We agree that our previous response did not adequately explain the discrepancy. Please note that the final SAP does not mention stopping rules. We appreciate the reviewer's emphasis on transparency and have revised the manuscript accordingly.

Statistical analysis section:
Safety oversight was provided by the independent Data Safety Monitoring Board. The preliminary Statistical Analysis Plan stated the development of clear stopping rules at approximately 50% and 75% of the planned sample size using pre-specified stopping criteria. However, in consultation with the Data Safety Monitoring Board we agreed that clear stopping rule are difficult to apply for a non-clinical intervention like training and quality improvement which is universally perceived as valuable why we only monitored the Caesarean section rates.

Limitations:
We analyzed the Cesarean section rate at two time points together with our data safety and monitoring board as a strong increase would have pointed to a negative effect but had no

other pre-defined stopping rules in view of the type of the intervention. No alpha-spending procedures were used and no trial decisions were based on interim statistical testing.
